# Clinical Effectiveness and Cost-Effectiveness of Supported Mindfulness-Based Cognitive Therapy Self-help Compared With Supported Cognitive Behavioral Therapy Self-help for Adults Experiencing Depression: The Low-Intensity Guided Help Through Mindfulness (LIGHTMind) Randomized Clinical Trial

**DOI:** 10.1001/jamapsychiatry.2023.0222

**Published:** 2023-03-22

**Authors:** Clara Strauss, Anna-Marie Bibby-Jones, Fergal Jones, Sarah Byford, Margaret Heslin, Glenys Parry, Michael Barkham, Laura Lea, Rebecca Crane, Richard de Visser, Amy Arbon, Claire Rosten, Kate Cavanagh

**Affiliations:** 1 School of Psychology, University of Sussex, Falmer, United Kingdom; 2 R&D Department, Sussex Education Centre, Sussex Partnership NHS Foundation Trust, Hove, United Kingdom; 3 School of Health Sciences, University of Brighton, Brighton, United Kingdom; 4 Salmons Institute for Applied Psychology, Canterbury Christ Church University, Tunbridge Wells, United Kingdom; 5 Sussex Partnership Foundation NHS Trust, Worthing, United Kingdom; 6 King’s Health Economics, Institute of Psychiatry, Psychology & Neuroscience, King’s College London, London, United Kingdom; 7 School of Health and Related Research, University of Sheffield, Sheffield, United Kingdom; 8 Clinical and Applied Psychology Unit, Department of Psychology, University of Sheffield, Sheffield, United Kingdom; 9 Centre for Mindfulness Research and Practice, School of Psychology, Bangor University, Bangor, United Kingdom; 10 University Hospitals Sussex NHS Trust, Royal Sussex County Hospital, Brighton, United Kingdom; 11 Brighton & Sussex Clinical Trials Unit, Watson Building, University of Brighton, Brighton, United Kingdom; 12 School of Sport and Health Sciences, University of Brighton, Brighton, United Kingdom

## Abstract

**Importance:**

Depression is prevalent. Treatment guidelines recommend practitioner-supported cognitive behavioral therapy self-help (CBT-SH) for mild to moderate depression in adults; however, dropout rates are high. Alternative approaches are required.

**Objective:**

To determine if practitioner-supported mindfulness-based cognitive therapy self-help (MBCT-SH) is superior to practitioner-supported CBT-SH at reducing depressive symptom severity at 16 weeks postrandomization among patients with mild to moderate depression and secondarily to examine if practitioner-supported MBCT-SH is cost-effective compared with practitioner-supported CBT-SH.

**Design, Setting, and Participants:**

This was an assessor- and participant-blinded superiority randomized clinical trial with 1:1 automated online allocation stratified by center and depression severity comparing practitioner-supported MBCT-SH with practitioner-supported CBT-SH for adults experiencing mild to moderate depression. Recruitment took place between November 24, 2017, and January 31, 2020. The study took place in 10 publicly funded psychological therapy services in England (Improving Access to Psychological Therapies [IAPT]). A total of 600 clients attending IAPT services were assessed for eligibility, and 410 were enrolled. Participants met diagnostic criteria for mild to moderate depression. Data were analyzed from January to October 2021.

**Interventions:**

Participants received a copy of either an MBCT-SH or CBT-SH workbook and were offered 6 support sessions with a trained practitioner.

**Main Outcomes and Measures:**

The preregistered primary outcome was Patient Health Questionnaire (PHQ-9) score at 16 weeks postrandomization. The primary analysis was intention-to-treat with treatment arms masked.

**Results:**

Of 410 randomized participants, 255 (62.2%) were female, and the median (IQR) age was 32 (25-45) years. At 16 weeks postrandomization, practitioner-supported MBCT-SH (n = 204; mean [SD] PHQ-9 score, 7.2 [4.8]) led to significantly greater reductions in depression symptom severity compared with practitioner-supported CBT-SH (n = 206; mean [SD] PHQ-9 score, 8.6 [5.5]), with a between-group difference of −1.5 PHQ-9 points (95% CI, −2.6 to −0.4; *P* = .009; *d* = −0.36). The probability of MBCT-SH being cost-effective compared with CBT-SH exceeded 95%. However, although between-group effects on secondary outcomes were in the hypothesized direction, they were mostly nonsignificant. Three serious adverse events were reported, all deemed not study related.

**Conclusions and Relevance:**

In this randomized clinical trial, practitioner-supported MBCT-SH was superior to standard recommended treatment (ie, practitioner-supported CBT-SH) for mild to moderate depression in terms of both clinical effectiveness and cost-effectiveness. Findings suggest that MBCT-SH for mild to moderate depression should be routinely offered to adults in primary care services.

**Trial Registration:**

isrctn.org Identifier: ISRCTN13495752

## Key Points

**Question:**

Is practitioner-supported mindfulness-based cognitive therapy self-help (MBCT-SH) clinically effective and cost-effective compared with practitioner-supported cognitive behavioral therapy self-help (CBT-SH) for adults experiencing mild to moderate depression?

**Findings:**

In this randomized clinical trial of 410 participants with mild to moderate depression, practitioner-supported MBCT-SH led to significantly greater reductions in depressive symptom severity at 16 weeks postrandomization compared with practitioner-supported CBT-SH. The probability of MBCT-SH being cost-effective compared with CBT-SH exceeded 95%.

**Meaning:**

Practitioner-supported MBCT-SH for mild to moderate depression was clinically effective and cost-effective compared with currently recommended practitioner-supported CBT-SH and should be made routinely available to adults experiencing mild to moderate depression.

## Teaser

This randomized clinical trial evaluates whether practitioner-supported mindfulness-based cognitive therapy self-help is superior to practitioner-supported cognitive behavioral therapy self-help at reducing depressive symptom severity at 16 weeks postrandomization among patients with mild to moderate depression.

## Introduction

Depression has a lifetime prevalence of 10.8% worldwide.^1^ It is often recurrent^2^ and has significant personal and economic consequences.^3^ Despite this, there is a well-established treatment gap whereby most people with depression do not have access to evidence-based treatments.^4^ Effective and scalable interventions are therefore needed.

To widen access, cognitive behavioral therapy self-help (CBT-SH) supported by a trained practitioner is recommended in the treatment of mild to moderate depression in national treatment guidelines.^5,6^ CBT for depression explores and evaluates the interrelationships between thoughts, feelings, physical sensations, and behaviors in the maintenance of depression, along with the historical antecedents of unhelpful thinking patterns. The effectiveness of specialist CBT for depression offered by highly trained CBT therapists is well established^7,8^; however, CBT-SH appears less acceptable, with higher rates of dropout.^9^ Alternatives to supported CBT-SH for depression are therefore needed.

Mindfulness-based cognitive therapy (MBCT) is an in-person group program recommended in national treatment guidelines for depression.^6,10^ Mindfulness involves deliberately bringing nonjudgmental awareness to present-moment experiences (eg, thoughts, feelings, physical sensations, behavioral urges), and this skill can be cultivated through mindfulness practice. In MBCT, daily mindfulness practice supported by verbal guidance and therapist-led discussion is combined with CBT for depression. Mindfulness practice is thought to facilitate greater awareness of the depressive maintenance cycle while also fostering a nonjudgmental attitude toward present-moment experiences and a recognition of thoughts as mental events, thereby potentially enhancing and broadening mechanisms targeted in CBT for depression. Meta-analyses of RCTs show MBCT is effective for depression, both in terms of reducing the relative risk of relapse for people with a history of recurrent depression^11^ and in terms of reducing the severity of symptoms for people experiencing a current episode of depression.^12^ However, as with all specialist psychological therapies, MBCT is not widely available in publicly funded health services,^13^ and the fixed group session times and locations mean that, even when available, it is not universally accessible (eg, for shift workers or those with caring responsibilities).

Practitioner-supported MBCT-SH is one solution to widen access. MBCT-SH overcomes the barrier of fixed group session times, enabling people with inflexible work or domestic commitments to engage at a convenient time and place. Evidence is emerging supporting the potential of MBCT-SH for depression. A randomized clinical trial (RCT) with students found that unsupported-MBCT-SH was superior compared with waitlist in reducing depression symptom severity,^14^ and another RCT found practitioner-supported MBCT-SH superior in reducing depression symptom severity compared with usual care for adults experiencing residual symptoms of depression.^15^ However, research to date does not answer the critical question of whether MBCT-SH for depression is clinically effective and cost-effective compared with the currently recommended practitioner-supported CBT-SH. If MBCT-SH is clinically effective and cost-effective in comparison, this would have significant implications for widening access to effective help.

We report findings from an RCT examining the clinical and cost-effectiveness of practitioner-supported MBCT-SH compared with practitioner-supported CBT-SH for adults experiencing mild to moderate depression. The prespecified primary hypothesis^16^ was that practitioner-supported MBCT-SH, in comparison with practitioner-supported CBT-SH, would lead to greater reductions in depressive symptom severity from baseline to 16 weeks postrandomization. It was also hypothesized that MBCT-SH would be cost-effective in comparison with CBT-SH at follow-up and that MBCT-SH will be a safe alternative to CBT-SH, with a similarly low incidence of serious adverse events and lasting negative effects.

## Method

### Design

This was a multicenter, pragmatic, assessor- and participant-blinded (participants were unaware of which intervention was hypothesized to be superior), parallel, superiority RCT with 1:1 allocation comparing practitioner-supported MBCT-SH with CBT-SH for adults experiencing mild to moderate depression in 10 Improving Access to Psychological Therapies (IAPT) services in England. The trial was preregistered, and the trial protocol was published.^16^ The trial protocol can be found in Supplement 1, and the statistical analysis plan can be found in Supplement 2. Ethical approval was given by the Health Research Authority London-Surrey Research Ethics Committee. Participants completed measures at baseline, 16 weeks postrandomization (postintervention), and 42 weeks postrandomization (follow-up). For further details of secondary hypotheses, see the eMethods in Supplement 3. This study followed the Consolidated Standards of Reporting Trials (CONSORT) reporting guideline.

### Participants

Inclusion criteria were that participants (1) were 18 years or older; (2) met criteria on the Clinical Interview Schedule–Revised (CIS-R)^17^ for a primary diagnosis of a depressive episode, mixed anxiety and depression, or nonspecified mild neurotic disorder^17^; (3) scored 10 or more points (clinical cutoff) on the Patient Health Questionnaire (PHQ-9) for depression^18^ at initial IAPT assessment; and (4) self-reported sufficient literacy skills to use English-language self-help materials. Exclusion criteria were that participants: (1) scored 20 or more points (severe range) on the PHQ-9; (2) scored 4 on the CIS-R suicidality scale; and (3) expressed a strong preference for one intervention over the other such that, if randomized to the nonpreferred intervention, they would likely decline treatment. Race and ethnicity data were collected by using a questionnaire with the following options as recommended by the Office for National Statistics in the UK^19^: Asian or Asian British (including Bangladeshi, Chinese, Indian, Pakistani, or any other); Black, African, Caribbean, or Black British (including African, Caribbean, or any other); mixed/multiple ethnic groups (including White and Asian, White and Black African, White and Black Caribbean, and any other); White (including English, Welsh, Scottish, Northern Irish, British, Gypsy or Irish Traveler, Irish, or any other), other ethnic group (including Arab or any other), and prefer not to say.

### Key Outcomes

#### Diagnostic Status

The CIS-R^17^ was conducted at baseline by the study research assistants. This diagnostic tool is routinely used in primary care mental health research^20^ and has been validated for synchronous telephone completion.^21^

#### Primary Outcome

Depression symptom severity (PHQ-9)^18^ at 16 weeks, with measurements also at baseline and 42 weeks.

#### Safety

Serious adverse events were recorded in line with Health Research Authority (England) guidelines and judged by an independent monitor as study related or study unrelated. An adapted version of the Lasting Negative Effects Questionnaire was completed at 42 weeks.^22^

#### Health Economic Evaluation

Health-related quality of life was assessed at baseline, 16 weeks, and 42 weeks using the recently developed 5-level version EuroQoL (EQ-5D-5L) to maximize sensitivity.^23^ Service use data on all health and social care services were collected using an adapted online version of the Adult Service Use Schedule (AD-SUS) developed in previous research for use with people with common mental health problems.^24,25^ The AD-SUS covered the previous 3 months at baseline (with research assistant support) and the period since last time point at 16 weeks and 42 weeks (self-completed; research assistant support available if requested).

#### Secondary Outcomes

Generalized anxiety, well-being, functioning, and mindfulness were measured at baseline, 16 weeks and 42 weeks. See the eMethods in Supplement 3 for details.

### Procedure

Referrals were from the IAPT program. People experiencing depression were also invited through general practitioners and social media to self-refer to IAPT and, if eligible for self-help treatment in IAPT, were assessed for eligibility. Potential participants had a copy of the participant information sheet and the opportunity to ask questions before giving informed consent.

Participants completed measures online (with postal option). Participants completed baseline measures with a research assistant present in person or by phone. At the end of baseline, eligible participants were randomized. Participants were then sent their allocated self-help workbook and asked to guide themselves through with 6 Psychological Well-being Practitioner (PWP) 30-minute to 45-minute support sessions. Up to 16 weeks was given to complete each intervention to allow for breaks and holidays. The PWP support sessions were offered by phone or face-to-face, depending on service practice and, where services offered a choice, participant preference.

Participants had the option to complete assessments at 16 weeks and 42 weeks with a research assistant present in person or by phone or on their own. Where 16-week and 42-week assessments were not completed, weekly reminders were sent for up to 1 month.

### Randomization and Masking

Participants were randomly assigned using Sealed Envelope^26^ stratified by IAPT service and initial PHQ-9 category (mild depression, 10-14 points; moderate depression, 15-19 points) using random permuted blocks of size 2, 4, or 6. The system was tested by the trial statistician (A.-M. B.-J.), who had no further involvement. Randomization was completed at the end of baseline, and participants were informed of their allocation by a research assistant. Assessors were typically not present at 16-week or 42-week online assessments, minimizing risk of bias. Where research assistants were present at 16 and 42 weeks, they were blind to allocation. If research assistants became unblinded, they were replaced by another blinded research assistant. Participants were not told the direction of hypotheses in relation to their randomized arm, only that the study was comparing 2 types of cognitive therapy. Analyses were carried out blind to study arm. Study PWPs were not blind to allocation.

### Interventions

*The Mindful Way Workbook: An 8-Week Program to Free Yourself from Depression and Emotional Distress*,^27^ written by the pioneers of MBCT, presents the MBCT course as a self-help workbook. *Overcoming Depression and Low Mood, 3rd Edition: A Five Areas Approach*^28^ has evidence demonstrating its effectiveness in reducing depression symptom severity^29^ and is widely used in IAPT. Use of the self-help books was supported by 6 structured PWPs sessions.

### Supervision and Monitoring

In IAPT, PWPs are graduates (in any discipline) who complete a year-long CBT-SH training. The 69 study PWPs received training to support MBCT-SH for this study. Training in the study involved qualified PWPs completing an MBCT course in person or using the workbook and then attending a 2-day MBCT-SH training. As is standard in MBCT, PWPs were encouraged to cultivate their own mindfulness practice.

The same PWPs delivered both interventions to account for therapist effects.^30^ To minimize therapeutic drift and therapy contamination, detailed PWP session-by-session protocols were provided, and PWPs were offered fortnightly telephone group supervision to cover both approaches with supervision led by clinical psychologists with doctoral training and trained in both CBT and MBCT with IAPT experience (C. S. and F. J.). Supervisors received monthly supervision of their supervision as additional quality assurance (R. C.).

### Lived-Experience Involvement

People with lived experience of depression and of CBT and mindfulness were involved in study development. This included contributing to the project proposal, recruitment materials, and qualitative data collection and analysis. A Lived Experience Advisory Panel of 6 members was led by the lived experience coapplicant who also helped train PWPs. See eAppendix 2 in Supplement 3 for further details.

### Statistical Analysis

The study was powered to detect a between-group standardized effect of 0.36 at 16 weeks on the primary outcome. This was based on the difference between the reported between-group effect of CBT-SH (0.42)^31^ and the between-group effect of MBCT-SH (0.78)^32^ on depressive symptom severity. The prespecified sample size of 410 provided 90% power to detect this effect with a 2-sided 5% alpha, allowing for 20% attrition. A data monitoring and ethics committee and a Trial Steering Committee provided trial oversight.

#### Clinical Outcomes

The primary analysis of clinical outcomes was an intention-to-treat (ITT) between-group comparison of MBCT-SH to CBT-SH at 16 weeks and 42 weeks using a linear mixed model with treatment group, time (16 weeks or 42 weeks), and a treatment group × time interaction as fixed factors and site and baseline PHQ-9 score as covariates. Participants were included as random effects. The ITT sample was composed of all participants and observed data, and individuals were analyzed as per randomization allocation. Secondary outcomes were estimated using linear mixed models and contrasts. Secondary per-protocol analyses were conducted for intervention completers (attending 3 or more PWP sessions). As a sensitivity check, missing data were assumed to be missing at random and dealt with using multiple imputation by chained equations and compared with observed-cases findings. Multilevel logistic regression compared treatment completion between groups controlling for center and baseline PHQ-9 score and are presented as adjusted odds ratios. Mediation analyses tested whether treatment completion mediated depressive symptom severity outcomes. Unplanned subgroup analyses were conducted to estimate PHQ-9 treatment effects at 16 weeks by sex, race and ethnicity, and medication use. Statistical analysis was conducted using Stata version 16 (StataCorp).

#### Health Economic Analysis

The UK National Health Service or personal social services perspective preferred by the National Institute for Health and Care Excellence^33^ was taken. The unit costs of the 2 interventions were estimated using a microcosting approach.^34^ Nationally applicable published unit costs were applied to all other services (eAppendix 1 in Supplement 3).

Cost-effectiveness was assessed through the calculation of incremental cost-effectiveness ratios explored in terms of quality-adjusted life-years (QALYs) calculated from the EQ-5D-5L.^35^ Uncertainty was explored using cost-effectiveness planes and cost-effectiveness acceptability curves based on the net-benefit approach.^36^ Sensitivity analyses explored the impact of missing data and outliers/influential outliers (eAppendix 1 in Supplement 3).

## Results

**Figure 1.**
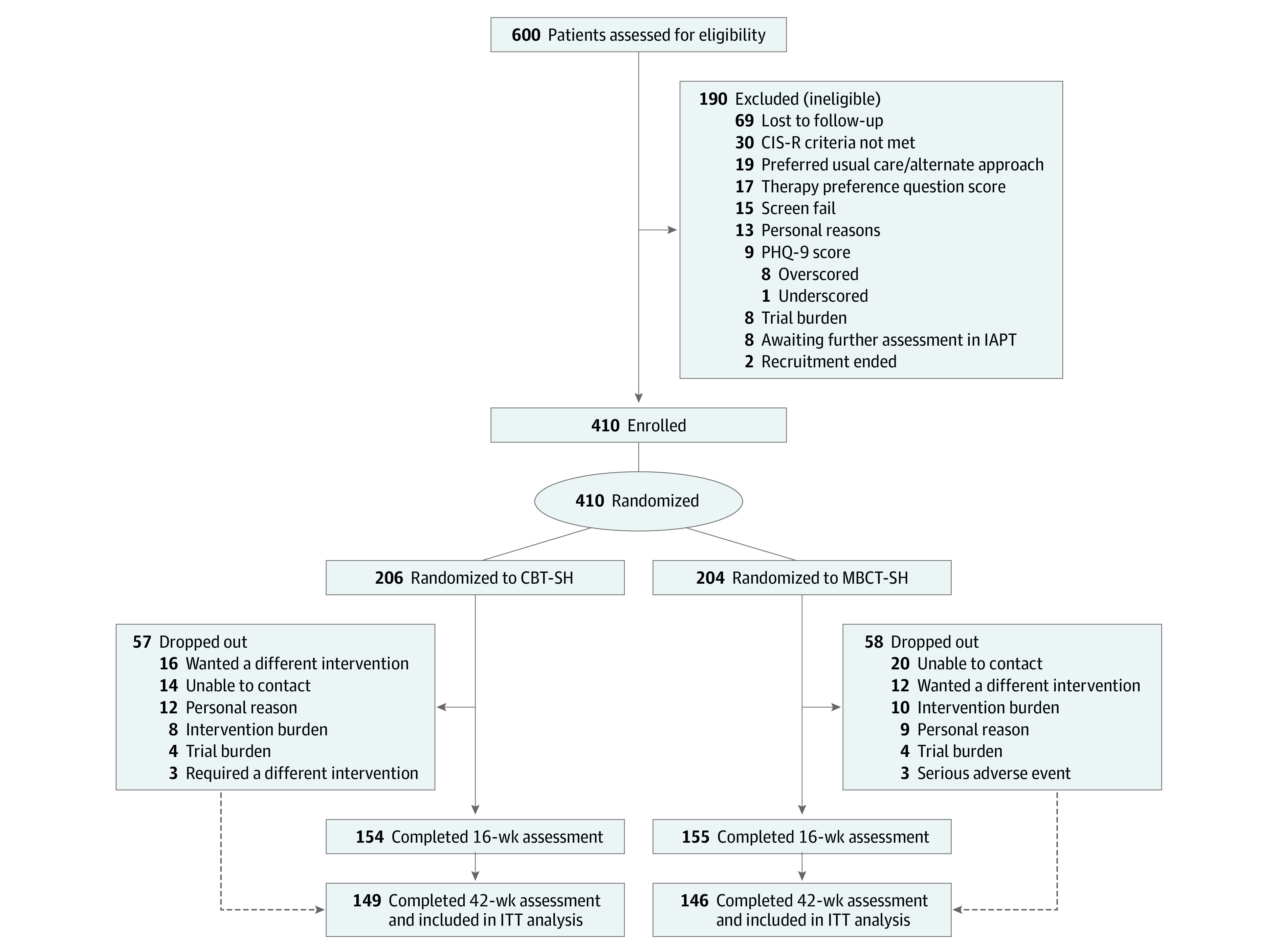
CONSORT Trial Profile CBT-SH indicates cognitive behavioral therapy self-help; CIS-R, Clinical Interview Schedule–Revised; IAPT, Improving Access to Psychological Therapies; ITT, intention to treat; MBCT-SH, mindfulness-based cognitive therapy self-help; PHQ-9, Patient Health Questionnaire.

In total, 600 people were assessed for eligibility. Of 410 randomized participants, 255 (62.2%) were female, and the median (IQR) age was 32 (25-45) years. Of the 410 participants, 204 were allocated to MBCT-SH and 206 to CBT-SH between November 24, 2017, and January 31, 2020, with final follow-up on December 15, 2020. A total of 17 participants (4.1%) were Asian or Asian British; 15 (3.7%) were Black, African, Caribbean, or Black British; 20 (4.9%) were mixed/multiple ethnic groups; 351 (85.6%) were White British or White Irish; 5 (1.2%) were other ethnic groups; and 2 (0.5%) preferred not to say. Primary outcome data were available for 155 of 204 MBCT-SH participants (76.0%) and 154 of 206 CBT-SH participants (74.8%). At 42 weeks, 146 MBCT-SH participants (71.6%) and 149 CBT-SH participants (72.3%) provided data. See Figure 1 for details. There was baseline balance on all participant characteristics (Table 1).

**Table 1.** Descriptive Summary for Demographic Variables

Characteristic	No. (%)	
	MBCT-SH (n = 204)	CBT-SH (n = 206)
Sex		
Female	125 (61)	130 (62)
Male	79 (39)	76 (37)
Gender identity matches assignment at birth		
No	0	0
Yes	204 (100)	206 (100)
Age, median (IQR), y	35 (26-45.5)	32 (25-45)
Racial and ethnic group^a^		
Asian or Asian British	9 (4)	8 (4)
Black, African, Caribbean, or Black British	7 (3)	8 (4)
Mixed/multiple ethnic groups	9 (4)	11 (5)
White British or White Irish	176 (86)	175 (85)
Other	1 (0.5)	4 (2)
Prefer not to say	2 (1)	0
Sexual orientation		
Bisexual	10 (5)	8 (4)
Gay	3 (2)	13 (6)
Heterosexual	179 (88)	177 (86)
Lesbian	3 (2)	2 (1)
Identify as another term	2 (1)	3 (2)
Prefer not to say	7 (3)	3 (2)
Marital status		
Single	78 (38)	90 (44)
Married, cohabiting, or civil partnership	108 (53)	100 (49)
Separated, divorced, or widowed	16 (8)	15 (7)
Prefer not to say	2 (1)	1 (0.5)
Employment status		
Employed	154 (75)	154 (75)
Not looking for work (unemployed, retired, carer, or volunteer)	24 (12)	16 (8)
Student	15 (7)	19 (9)
Unemployed and looking for work	11 (5)	17 (8)
Highest education level		
No educational qualification	5 (2)	3 (1)
GCSE or equivalent (qualifications taken at 16 y)	37 (18)	45 (22)
A Levels or equivalent (qualifications taken at 18 y)	54 (26)	62 (30)
University-level education	107 (52)	93 (45)
Prefer not to say	1 (0.5)	3 (1)
Symptom severity, PHQ-9		
Mild	95 (47)	101 (49)
Moderate	109 (53)	105 (51)
Site		
Site 1	30 (15)	32 (16)
Site 2	12(6)	14 (7)
Site 3	10 (5)	9 (4)
Site 4	4 (2)	4 (2)
Site 5	19 (9)	19 (9)
Site 6	13 (6)	14 (7)
Site 7	25 (12)	24 (12)
Site 8	52 (26)	51 (25)
Site 9	3 (2)	2 (1)
Site 10	36 (18)	37 (18)

Abbreviations: CBT-SH, cognitive behavioral therapy self-help; GCSE, General Certificate of Secondary Education; MBCT-SH, mindfulness-based cognitive therapy self-help; PHQ, Patient Health Questionnaire.

^a^ Participants were invited to select from the following options: Asian or Asian British (including Bangladeshi, Chinese, Indian, Pakistani, or any other); Black, African, Caribbean, or Black British (including African, Caribbean, or any other); mixed/multiple ethnic groups (including White and Asian, White and Black African, White and Black Caribbean, and any other); White (including English, Welsh, Scottish, Northern Irish, British, Gypsy or Irish Traveler, Irish, or any other), other ethnic group (including Arab or any other), and prefer not to say.

Table 2 gives a descriptive summary of clinical outcomes, effect estimates, and effect sizes for the prespecified primary ITT analysis on clinical outcomes, and the eFigure in Supplement 3 shows effects of PHQ-9 by trial arm and time point. The prespecified primary outcome (depression symptom severity) at the prespecified primary end point (16 weeks) showed statistically significant superiority in favor of MBCT-SH (mean [SD] PHQ-9 score, 7.2 [4.8] points vs 8.6 [5.5] points; between-group difference, −1.5 points; 95% CI, −2.6 to −0.4; *P* = .009; *d* = −0.36).

**Table 2.** Descriptive Summary of Clinical Outcomes, Effect Estimates, and Effect Sizes With Observed-Cases Intention-to-Treat Analysis

Group	Baseline		16 wk		42 wk		Between-group difference (95% CI)		Cohen *d*		*P* value^a^		*P* value ^b^	
	Total, No.	Mean (SD)	Total, No.	Mean (SD)	Total, No.	Mean (SD)	16 wk	42 wk	16 wk	42 wk	16 wk	42 wk	16 wk	42 wk
**PHQ-9**														
MBCT-SH	204	14.50 (4.09)	155	7.15 (4.82)	146	6.49 (4.87)	−1.46 (−2.55 to −0.36)	−0.94 (−2.05 to 0.17)	−0.36	−0.23	.009	.10	.02	.10
CBT-SH	205	14.90 (4.03)	154	8.56 (5.49)	148	7.41 (5.28)								
**GAD-7**														
MBCT-SH	204	10.30 (4.4)	155	5.85 (4.38)	146	5.18 (4.27)	−0.95 (−1.88 to −0.01)	−0.79 (−1.74 to 0.15)	−0.23	−0.19	.047	.10	.08	.13
CBT-SH	205	11.10 (3.94)	154	6.96 (4.68)	149	6.24 (4.71)								
**SWEMWS**														
MBCT-SH	204	10.40 (3.55)	155	15.70 (4.89)	146	16.4 (4.94)	0.97 (−0.05 to 1.99)	0.70 (−0.33 to 1.73)	0.29	0.21	.06	.18	.16	.17
CBT-SH	205	10.70 (3.1)	154	15.10 (4.87)	149	15.80 (4.75)								
**WSAS**														
MBCT-SH	204	21.00 (6.54)	155	11.80 (8.12)	146	11.20 (8.17)	−1.39 (−3.05 to 0.28)	−1.00 (−2.69 to 0.69)	−0.21	−0.15	.10	.25	.18	.25
CBT-SH	204	19.50 (6.79)	154	12.70 (8.00)	148	12.00 (7.94)								
**FFMQ-15 Observe**														
MBCT-SH	204	8.44 (2.87)	155	9.76 (2.42)	146	9.95 (2.33)	0.27 (−0.20 to 0.75)	0.16 (−0.32 to 0.65)	0.10	0.06	.26	.51	.63	.64
CBT-SH	206	8.56 (2.45)	154	9.68 (2.38)	148	9.93 (2.36)								
**FFMQ-15 Describe**														
MBCT-SH	204	8.12 (2.7)	155	9.87 (2.57)	146	9.82 (2.61)	0.37 (−0.11 to 0.85)	−0.02 (−0.5 to 0.47)	0.14	−0.01	.13	.95	.23	.81
CBT-SH	206	8.45 (2.59)	154	9.58 (2.54)	148	9.91 (2.66)								
**FFMQ-15 Aware**														
MBCT-SH	204	8.42 (2.17)	155	9.10 (2.15)	146	9.27 (2.24)	−0.11 (−0.56 to 0.33)	−0.22 (−0.68 to 0.23)	−0.05	−0.10	.63	.34	.55	.29
CBT-SH	206	8.19 (2.2)	154	9.06 (2.22)	148	9.33 (2.35)								
**FFMQ-15 Nonjudge**														
MBCT-SH	204	8.42 (2.17)	155	10.50 (2.78)	146	11.10 (2.65)	0.71 (0.15 to 1.28)	0.66 (0.08 to 1.23)	0.27	0.25	.01	.03	.03	.02
CBT-SH	205	8.21 (2.74)	154	9.89 (2.74)	148	10.30 (2.87)								
**FFMQ-15 Nonreacting**														
MBCT-SH	203	7.96 (2.73)	155	9.41 (2.37)	146	9.59 (2.31)	0.21 (−0.29 to 0.71)	0.20 (−0.31 to 0.71)	0.08	0.08	.42	.44	.76	.74
CBT-SH	205	8.02 (2.31)	154	9.27 (2.63)	148	9.47 (2.30)								
**FFMQ-15 total**														
MBCT-SH	203	32.70 (6.09)	155	38.90 (6.96)	146	39.8 (6.85)	1.22 (−0.20 to 2.64)	0.62 (−0.83 to 2.06)	0.20	0.10	.09	.40	.25	.41
CBT-SH	205	32.90 (6.06)	154	37.80 (7.01)	148	39.00 (7.40)								

Abbreviations: CBT-SH, cognitive behavioral therapy self-help; FFMQ-15, Five Facet Mindfulness Questionnaire; GAD-7, Generalized Anxiety Disorder; MBCT-SH, mindfulness-based cognitive therapy self-help; PHQ-9, Patient Health Questionnaire; SWEMWS, Short Warwick-Edinburgh Mental Well-Being Scale; WSAS, Work and Social Adjustment Scale.

^a^ *P* values reported are the treatment group × time interaction contrasts of marginal linear predictions for observed data.

^b^ *P* values reported are the treatment group × time interaction contrasts of marginal linear predictions for imputed data.

In terms of secondary outcomes following ITT analysis, effects on Generalized Anxiety Disorder 7-item scale at 16 weeks showed significant superiority in favor of MBCT-SH as did effects on the nonjudge subscale of the Five Facet Mindfulness Questionnaire at both 16 and 42 weeks. Effects on other secondary outcomes were nonsignificant, including PHQ-9 score at 42 weeks. See the eResults and eTables 1 to 7 in Supplement 3 for details of additional analyses of secondary outcomes and subgroup analyses.

There were 3 serious adverse events (1.5%) in the MBCT-SH arm and 0 in the CBT-SH arm. All serious adverse events were deemed unrelated to the intervention by the independent clinical reviewer. Lasting negative effects of the intervention were reported in the CBT-SH and MBCT-SH groups by 6 of 147 participants (4.1%) and 8 of 146 participants (5.5%), respectively (eTable 9 in Supplement 3). In the CBT-SH arm, 2 of 6 attributed these effects to face-to-face meetings and feeling pressure to progress through the workbook, and 1 of 8 in MBCT-SH arm reported not liking the workbook and exercises. The remaining 11 did not give reasons.

Mean costs per group over the 42-week follow-up are reported in Table 3. Mean intervention costs were similar in the 2 arms, £174 ($209) in the MBCT-SH arm and £168 ($202) in the CBT-SH arm. Other health and social care costs were significantly lower in the MBCT-SH arm (mean [SD] cost per participant, £769 [£1102] [$923]) compared with the CBT-SH arm (mean [SD] cost per participant, £1403 [£2759] [$1684]; adjusted mean difference, −£530 [$636]; 95% CI, −£917 to −£144; *P* = .007). This was due to greater use in the CBT-SH arm of individual psychological therapies received outside of the trial, outpatient contacts for mental health, general practitioner contacts, and psychotropic medication. This resulted in significantly lower total costs in the MBCT-SH group (mean [SD] cost, £944 [£1102] [$1133]) compared with the CBT-SH group (mean [SD] cost, £1571 [£2754] [$1886]; adjusted mean difference, −£526 [$631]; 95% CI, −£908 to −£14; *P* = .007). There were no significant differences in QALYs (adjusted mean difference, 0.01; 95% CI, −0.01 to 0.03; *P* = .48).

**Table 3.** Mean Costs per Participant Between Baseline and 42-Week Follow-up

Measure	Cost, mean (SD)		Unadjusted		Adjusted^a^	
	MBCT-SH (n = 145)	CBT-SH (n = 147)	Mean difference (95% CI)	*P* value	Mean difference (95% CI)	*P* value
Costs, £						
Intervention	174 (76)	168 (79)	7 (−11 to 24)	.47	4 (−13 to 22)	.61
Health and social care	769 (1102)	1403 (2759)	−634 (−1119 to −148)	.01	−530 (−917 to −144)	.007
Hospital services	297 (774)	487 (1809)	NA	NA	NA	NA
Community services	168 (404)	229 (380)	NA	NA	NA	NA
Psychological and talking therapies	277 (631)	658 (1531)	NA	NA	NA	NA
Medication	27 (45)	28 (37)	NA	NA	NA	NA
Total	944 (1102)	1571 (2754)	−627 (−1109 to −145)	.01	−526 (−908 to −144)	.007
Outcomes						
QALYs	0.65 (0.13)	0.65 (0.12)	0 (−0.03 to 0.03)	.82	0.01 (−0.01 to 0.03)	.48

Abbreviations: NA, not applicable; QALYs, quality-adjusted life years.

^a^ Adjusted by baseline variable of interest plus baseline utility, baseline PHQ-9, site, and follow-up time.

**Figure 2.**
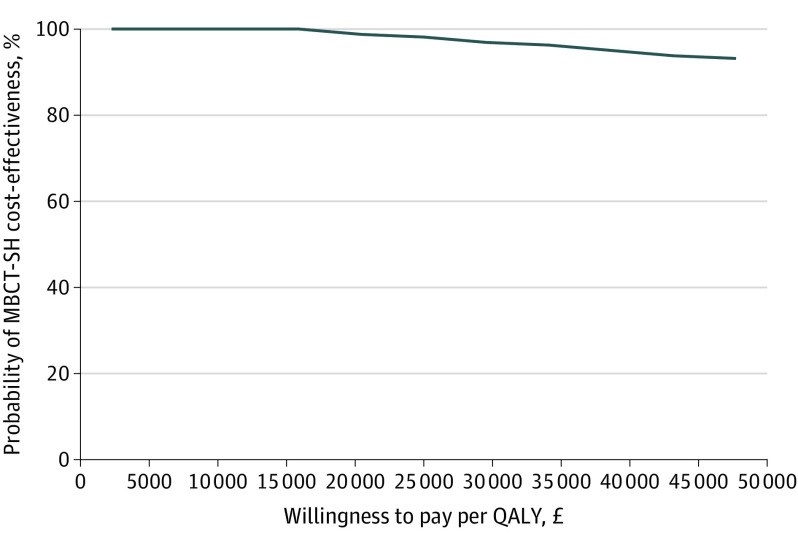
Cost-Effectiveness Acceptability Curve for Mindfulness-Based Cognitive Therapy Self-help (MBCT-SH) vs Cognitive Behavioral Therapy Self-help at 42 Weeks Postrandomization QALY indicates quality-adjusted life-year.

Cost-effectiveness analysis found MBCT-SH dominated CBT-SH—it generated better outcomes for lower cost. Figure 2 shows the cost-effectiveness acceptability curve, which suggests that the probability of MBCT-SH being cost-effective compared with CBT-SH is above 95% at the National Institute for Health and Care Excellence willingness-to-pay thresholds of £20 000 to £30 000 ($24 000 to $36 000) per QALY. These results were supported in sensitivity analyses exploring the impact of missing data and the impact of outliers. Full economic results can be found in eAppendix 1 in Supplement 3.

## Discussion

The primary hypothesis was supported: practitioner-supported MBCT-SH was superior to practitioner-supported CBT-SH in reducing depressive symptom severity at postintervention follow-up. In addition, PWP-supported MBCT-SH was found to be cost-effective compared with PWP-supported CBT-SH due to significantly lower total costs alongside similar QALY outcomes.

No serious adverse events attributable to the interventions were reported, and lasting negative effects were uncommon and similar in each arm, in line with previous psychological intervention research.^22^ This supports the hypothesis that practitioner-supported MBCT-SH is safe compared with practitioner-supported CBT-SH.

This study found that practitioner-supported MBCT-SH is not only more clinically effective than CBT-SH but also more cost-effective. On average, the CBT-SH intervention cost health services £526 ($631) more per participant than the MBCT-SH intervention over the 42-week follow-up. A substantial proportion of this additional cost (approximately 50%) was accounted for by additional face-to-face individual psychological therapy accessed by CBT-SH participants outside of the study intervention. If practitioner-supported MBCT-SH were offered as an alternative, it might be expected that some of the cost savings would fall on health services, potentially supporting increased treatment capacity.

Findings corroborate and extend findings from previous RCTs of MBCT-SH.^14,15^ While these previous trials found that MBCT-SH is effective in reducing the severity of depressive symptoms, they cannot answer the critical question of whether practitioner-supported MBCT-SH is a suitable alterative to the currently recommended practitioner-supported CBT-SH, either in terms of clinical effectiveness or cost-effectiveness. Moreover, while emerging evidence suggests that specialist MBCT and specialist CBT may be comparably effective for depression,^12,37^ our findings of superiority of MBCT-SH over CBH-SH suggest this may not be the case for practitioner-supported self-help versions of these therapies. Although dropout is higher for CBT-SH than CBT for depression,^9^ we did not find hypothesized between-group differences in intervention completion rates (PWP session attendance; eTable 8 in Supplement 3), and treatment completion did not mediate between-group differences on the primary outcome (eDiscussion in Supplement 3). Future research should therefore identify factors that contribute to the relative effectiveness of MBCT-SH over CBT-SH for depression. Exploring MBCT-SH engagement and predictors of engagement is an important avenue for future research. MBCT-SH, relative to CBT-SH, appears to have specific effects on acceptance (given effects on the nonjudge mindfulness subscale), and future quantitative and qualitative research should explore more fully the specific effects of MBCT-SH on depression-related outcomes.

In relation to secondary outcomes, we also found superior effects of supported MBCT-SH over CBT-SH on anxiety symptom severity at postintervention. At 42-week follow-up, between-group effects on depressive and anxiety symptom severity remained in the hypothesized direction but were nonsignificant. This could in part by explained by the greater postintervention psychological therapy accessed by CBT-SH participants. Lack of effects at postintervention and follow-up on all but the nonjudge mindfulness subscale was not expected. This potentially suggests that MBCT-SH relative to CBT-SH is effective at improving the specific mindfulness capacity to be accepting of unpleasant thoughts and emotions but not other aspects of the mindfulness construct. Existing measures of mindfulness may need reevaluating to improve their ability to discriminate the specific effects of MBCT.^38^

This trial recruited across multiple services representing a broad range of geographic, rural and urban, and sociodemographic characteristics, suggesting that findings can be generalized to other psychological therapy services. The research team included researchers from a predominantly CBT background, an MBCT background, and from both traditions, reducing allegiance effects. Assessments were conducted blind to treatment allocation, participants were not informed about the hypothesized direction of effects, and data analysis was conducted with treatment arm masked, all reducing risk of bias. This pragmatic trial was conducted in the real-world public mental health setting, training practitioners within services to deliver MBCT-SH. This points to feasibility of implementation of practitioner-supported MBCT-SH for depression.

Our findings suggest that offering practitioner-supported MBCT-SH as an intervention for mild to moderate depression would improve outcomes and save money compared with practitioner-supported CBT-SH. Therefore, practitioner-supported MBCT-SH should be routinely offered as an intervention for mild to moderate depression alongside practitioner-supported CBT-SH. This would increase patient choice, as currently only CBT-SH is typically recommended in treatment guidelines for depression.^6^ In this study, PWPs were trained and supervised to support MBCT-SH. For findings to translate into practice, it is important that implementation adheres to this training and supervision process.

Future research should aim to corroborate and extend findings, for example by including more frequent assessments to allow trajectory and mechanisms of change to be more fully explored. Implementing research findings in routine clinical practice is notoriously challenging. Future research should examine factors enabling successful implementation of practitioner-supported MBCT-SH to expedite the pathway to patient benefit and to ensure fidelity to the approach. Future research should include evaluating the relative effectiveness and acceptability of different formats of MBCT-SH for depression, including book-based (as in the current study) and online^15^ versions.

### Limitations

This study has limitations. Confidence in findings is limited by study dropout. However, this concern is partially mitigated by similar rates of study dropout across arms (eResults and eTables 1 and 2 in Supplement 3), use of ITT analysis as the primary analysis and multiple imputation methods used as a sensitivity check. Moreover, higher rates of study dropout are typical for RCTs of self-help psychological interventions for depression. For example, a recent meta-analysis of RCTs evaluating smartphone apps for depressive symptoms reported a mean study dropout rate of 47.8%, with strategies such as using paid assessments not improving dropout.^39^ While future research should aim to use methods to minimize study dropout, these higher rates should be anticipated and taken into account when determining sample size.

## Conclusions

In conclusion, this study found that a novel intervention, practitioner-supported MBCT-SH, was clinically superior in targeting depressive symptom severity at postintervention and cost-effective compared with the criterion standard of practitioner-supported CBT-SH for adults experiencing mild to moderate depression. This has important implications for the more than 100 000 people currently offered CBT-SH for depression in the IAPT program each year^8^ and in publicly funded services elsewhere. If study findings are translated into routine practice, this would see many more people recovering from depression while costing health services less money.

## Notes

Supplement 1. Trial Protocol

Supplement 2. Statistical Analysis Plan

Supplement 3.
eMethods.

eResults.

eDiscussion.
**eFigure.** PHQ-9 Scores by Trial Arm and Timepoint **eTable 1.** Descriptive Summary for Demographic Variables Comparing PHQ-9 T1 Data Completers With T1 Data Noncompleters at 16 Weeks **eTable 2.** Descriptive Summary of Baseline Clinical Measures Comparing PHQ-9 T1 Data Completers With T1 Data Noncompleters at 16 Weeks **eTable 3.** Descriptive Summary of Clinical Outcomes, Effect Estimates, and Effect Sizes for Observed-Cases Per-Protocol Analysis **eTable 4.** Between-Group Effect Estimates and Effect Sizes for Modified Intention-to-Treat Analysis With Observed Cases and Multiple Imputation **eTable 5.** Between-Group Effect Estimates and Effect Sizes for Per-Protocol Analysis With Observed Cases and Multiple Imputation **eTable 6.** Between-Group PHQ-9 Effect Estimates and Effect Sizes by Sex, Ethnicity, and Baseline Medication Use Using Observed Cases and Multiple Imputation **eTable 7.** Proportion of Participants in the Nonclinical Range (PHQ-9 score of 0-9) for Depressive Symptoms at 16 and 42 Weeks **eTable 8.** PWP Session Adherence **eTable 9.** Lasting Negative Effects by Study Arm **eAppendix 1.** Further Details of Health Economic Evaluation **eAppendix 2.** The Role of Experts by Experience
eReferences.

Supplement 4. Data Sharing Statement
