# Supplementary material for: Clinical Effectiveness and Cost-Effectiveness of Supported Mindfulness-Based Cognitive Therapy Self-help Compared With Supported Cognitive Behavioral Therapy Self-help for Adults Experiencing Depression: The Low-Intensity Guided Help Through Mindfulness (LIGHTMind) Randomized Clinical Trial
Source: JAMA Psychiatry. 2023 Mar 22;80(5):415–24. doi: 10.1001/jamapsychiatry.2023.0222 (PMC10034662; doi:10.1001/jamapsychiatry.2023.0222)
Supplement: Supplement 1. — Trial Protocol [file jamapsychiatry-e230222-s001.pdf]

LightMIND 2

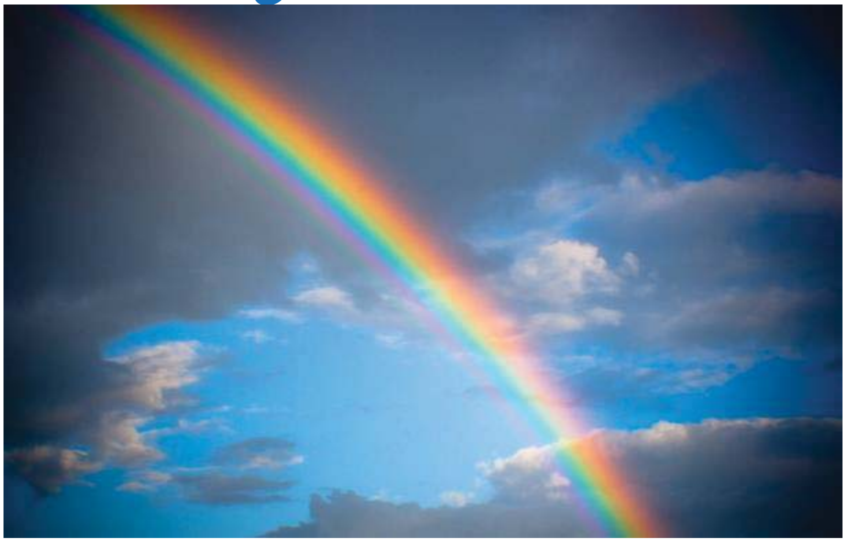

Low-Intensity Guided Help Through MINDfulness

A randomised controlled trial comparing supported Mindfulness-Based Cognitive Therapy self-help to supported Cognitive Behaviour Therapy self-help for adults experiencing depression

RESEARCH PROTOCOL
14 February 2019
Version 7

Version History	Date	Summary of Protocol Amendment
1	15 March 2017	Clarification of assessor blinding
2	8 June 2017	
3	17 January 2018	Clarification of safety reporting and eligibility screening
4	08 February 2018	GP practices to offer study and provide screening
5	02 May 2018	Clarification of change interview timings, and audio recordings.
6	22 November 2018	Clarification of adherence ratings procedures for audio recordings, definition of treatment completer, number of participants in Change Interview.

7	February 2019	Clarification of the collection of screening data from screen-failed participants and collection of population demographic data from the IAPT services.

**Study Title:**	Low-Intensity Guided Help Through MINDfulness: A Randomised Controlled Trial Comparing Supported Mindfulness-Based Cognitive Therapy Self-Help to Supported Cognitive Behaviour Therapy Self-Help for Adults Experiencing Depression	
**Study Acronym:**	LIGHTMind 2	
**Chief Investigator**:	Dr Clara Strauss	*clara.strauss@nhs.net*
**Research Team**	Michael Barkham	*m.barkham@sheffield.ac.uk*
**(alphabetical order)**:	Sarah Byford	*s.byford@kcl.ac.uk*
	Kate Cavanagh	*kate.cavanagh@sussex.ac.uk*
	Rebecca Crane	*r.crane@bangor.ac.uk*
	Anna-Marie Jones	a.*jones4@brighton.ac.uk*
	Fergal Jones	*fergal.jones@sussexpartnership.nhs.uk*
	Laura Lea	*laura.lea@sussexpartnership.nhs.uk*
	Glenys Parry	*g.d.parry@sheffield.ac.uk*
	Claire Rosten	*c.e.rosten@brighton.ac.uk*
	Richard de Visser	r.*de-visser@sussex.ac.uk*

**Trial Manager**	Amy Arbon	*amy.cm.arbon@bsuh.nhs.uk*
**Study Sponsor**	Sussex Partnership NHS Foundation Trust<br>Swandean, Arundel Road, Worthing, West Sussex, BN13 3EP	
**Trial Registration**	The trial will be registered with ISRCTN before starting recruitment.	
**Funding**	This trial has been funded by NIHR through the Research for Patient Benefit Funding Stream.<br>Reference number PB-PG-0815-20056	
**Protocol guidelines**	This protocol has been written in line with the SPIRIT checklist (2013)	

Contents

1 Abstract and Lay Summary 5
2 Keywords 5
3 List of abbreviations 6
4 Background 7
4.1 An Alternative Approach 7
4.2 Research question and hypotheses 8
5 Patient and Public Involvement (PPI) 9
5.1 PPI to date 9
5.2 Current PPI 11
6 Methods/Design 12
6.1 Design 12
6.2 Participants 12
6.2.1 Inclusion/exclusion criteria 13
6.3 Intervention protocols 13
6.3.1 MBCT Self-Help 13
6.3.2 CBT Self-help 14
6.3.3 Intervention Fidelity and Adherence 14
6.4 Primary & Secondary Outcome Measures 14
6.4.1 Demographic Information 15
6.4.2 Diagnosis 15
6.4.3 Primary outcome measure 15
6.4.4 Secondary outcome measures 15
6.4.5 Intervention Evaluation Measures and Tools 16
6.4.6 Health economic measures 16
6.4.7 Qualitative evaluation 16
6.5 Recruitment and consent methods....................16
6.6 Assessment process....................18
6.7 Procedure....................18
6.8 Randomisation process & allocation concealment....................19
7 Data Management & Analysis 19
7.1 Summary of the Types of Data 19
7.2 Research Variables Form (RVF) 19
7.3 Sample size & Power calculations 210
7.4 Planned data analysis 221
7.5 Dummy results tables 233
7.6 Data collection, entering, coding and checking process 29
7.7 Missing data policy 29
7.8 Data custodian and data ownership 31
7.9 Data quality and Standards 31
7.10 Data security 31
7.11 Data sharing 32
8 Project management 32
8.1 Trial Management 32
8.2 Data Management 32
8.3 Financial Management 33

8.4 Trial Oversight 33
9 Ethical considerations 33
10 Data monitoring for harm 33
10.1 Investigator Assessment 35
11 Discussion of practical and operational issues 37
12 Project timetable 37
13 Projected outputs and Dissemination 38
14 Plans for Translation 39
14.1 Expected Output of Research/Impact 39
15 Gantt Chart 39
16 Amendments 421
17 Competing interests 421
18 Acknowledgements 421
19 References 472
20 Appendices 46

1 Abstract and Lay Summary

Depression has serious personal, family and economic consequences. It is estimated that depression will cost £12.15 billion to the economy each year in England by 2026. Improving Access to Psychological Therapies (IAPT) is the NHS talking therapies service in England for adults experiencing anxiety or depression. Approximately 1 million people are referred to IAPT every year, over half experiencing depression. Where symptoms of depression are mild/moderate people are typically offered Cognitive Behaviour Therapy (CBT) self-help supported by a psychological wellbeing practitioner (PWP).

The problem is that over half of people (58%) who complete treatment for depression in IAPT remain depressed despite receiving the NICE-recommended treatment. Furthermore, less than half (40%) of IAPT clients complete treatment. This study seeks to investigate an alternative to CBT self-help. We suggest that mindfulness-based self-help – which differs from CBT in focus, approach and practice – would be more effective with lower dropout. We conducted a study with 40 people comparing these two forms of self-help (CBT and Mindfulness). We found people using mindfulness-based self-help showed greater improvement in depression and twice as many people completed mindfulness-based self-help.

In this proposed study, 410 people experiencing mild-moderate depression will be randomly allocated to using a mindfulness-based self-help workbook or to a CBT-based self-help workbook. Each person will be asked to complete their workbook within 16 weeks and will have six PWP support sessions during this time. The primary outcome we will measure is depression symptom severity. Treatment completion will also be measured because evidence shows completing treatment is associated with better outcomes. Assessments will be made at the start of the study and then again after 16 weeks and then after a further 6 months and 40 participants will also be interviewed about their experiences after the 6 month follow up assessment period. Findings will provide evidence for policy makers and will help to inform decision making about mindfulness-based self-help in the NHS.

2 Keywords

Depression; mindfulness; cognitive therapy; self-help

3 List of abbreviations

ADSUS	Adult Service Use Schedule
CBT	Cognitive Behavioural Therapy
CBT-SH	Cognitive Behavioural Therapy Self Help
CI	Chief Investigator
CIS-R	Clinical Interview Schedule
CONSORT	Consolidated Standards of Reporting Trials
CRF	Case Report Form
CRG	Clinical Research Group
CRN	Clinical Research Network
FFMQ-15	Five-Facet Mindfulness Questionnaire 15
GAD-7	Generalised Anxiety Disorder 7
IAPT	Improving Access to Psychological Therapies
ICER	Incremental Cost-Effectiveness Ratios
LEAP	Lived Experience Advisory Panel
MBCT-SH	Mindfulness Based Cognitive Therapy Self Help
MBI	Mindfulness Based Intervention
MHRN	Mental Health Research Network
PHQ-9	Patient Health Questionnaire 9
PPI	Patient and Public Involvement
PSSRU	Personal Social Services Research Unit
PWP	Psychological Wellbeing Practitioner
RA	Research Assistant
RCT	Randomised Controlled Trial
SWEMWS	Short Warwick-Edinburgh Mental Well-being Scale
TSC	Trial Steering Committee
WSAS	Work and Social Adjustment Scale

4 Background

Around 15% of adults in England experience clinically significant depression or anxiety in any week[1]. Improving Access to Psychological Therapies (IAPT) is an NHS initiative launched in 2006 that aims to improve access to psychological therapies for people experiencing anxiety and depression with around 1 million people now referred to IAPT each year[2].

The majority of people (55%) referred to IAPT experience some form of depression[3]. Depression is typically recurrent - following one episode of major depression 50% will relapse and after two episodes 80% relapse[4]. In addition to the impact on individuals and their families, depression is estimated to cost the economy in England £12.15 billion a year by 2026[5]. In order to meet the needs of people experiencing depression IAPT offer stepped-care[6] – supported self-help at Step 2 followed by, where needed, face-to-face therapy at Step 3. In line with National Institute of Health and Care Excellence (NICE)[7], at Step 2 people are provided with cognitive behaviour therapy self-help (CBT-SH) materials supported by a Psychological Wellbeing Practitioner (PWP).

However, IAPT have shown disappointing treatment outcomes for CBT self-help - only around 40%[3] of people completing treatment for depression meet criteria for remission. In addition, this figure is for initial remission and does not consider sustained recovery[8]. Partial remission from depression is associated with greater risk of relapse[9]. A related problem in IAPT is high rates of treatment drop-out - only around 40% of IAPT referrals complete a course of treatment[2] - completing treatment is important because it is associated with better outcomes[10]. Moreover, costs for treatment non-completers surpass that of treatment completers in IAPT[11]. Yet, there is poor understanding of reasons for non-completion[12] and this evidence gap needs addressing. Improving remission rates for depression and increasing treatment completion require urgent attention.

4.1 *An Alternative Approach*

Mindfulness is the capacity to intentionally pay attention, non-judgementally, to current experience. Mindfulness-based interventions (MBIs) teach the application of mindfulness in everyday life and work by reducing rumination and worry[13], well-established mechanisms of depression[14]. MBIs differ from CBT in important ways: (1) CBT includes evaluating the accuracy of difficult thoughts, MBIs encourage a self-compassionate, non-judgemental and accepting attitude towards experience, including unpleasant thoughts; (2) regular meditation practice (verbally guided attention towards present-moment experiences) is integral to MBIs but is not included in CBT – it is suggested that it is crucial to experience thoughts in the moment as transient mental events[15]; and (3) participant experience is different in terms of content and goals.

Mindfulness-based cognitive therapy (MBCT) is a Step 3 group therapy for depression recommended by NICE[16] that includes elements of CBT for depression. Meta-analyses show MBCT reduces the relative risk of relapse for people with a history of multiple episodes of depression by almost half (43%)[17] and mindfulness-based group interventions, in comparison to control conditions, lead to significant reductions in depression severity for people currently depressed[18]. Thus, MBCT groups are a good candidate for not only attaining initial symptom remission but also at achieving sustained recovery and preventing relapse. However, this evidence applies to Step 3 face-to-face interventions and we cannot assume this potential will generalise to a Step 2 self-help intervention.

MBCT self-help (MBCT-SH) in IAPT has the potential to reduce the cost of delivery and to widen access to people unable or unwilling to attend a group[19]. The potential of MBCT-SH as a clinically- and cost-effective treatment for depression in IAPT is the focus of this study and is the next step in our research programme.

To date we have completed the first two studies in our research programme.

In **Study 1** we evaluated MBCT-SH in a randomised controlled trial (RCT) with 80 students. We found MBCT-SH, in comparison to wait-list, led to significant improvements in depressive symptom severity[20].

Study 2 was a pilot RCT with 40 IAPT clients experiencing sub-threshold, mild or moderate depression (PHQ-9[21] 5-19 inclusive). We compared MBCT-SH[22] to CBT-SH[23] alongside four PWP support sessions. There were between-group differences in favour of MBCT-SH on improvements in depressive symptoms (Cohen's d=0.26) and the 95% confidence interval of this difference contained the minimum clinically important difference (MCID) of 2.59[24] (mean=1.25; 95% CI -2.46 to 4.96). Moreover, 61% (11/18) of MBCT-SH study completers showed reliable improvement[25] compared to only 38% (5/13) in the CBT-SH arm. Importantly, in terms of treatment drop-out, twice as many people dropped-out of treatment in the CBT-SH arm (10/20) as in the MBCT-SH arm (5/20). Feedback from the PPI consultation panel was that MBCT-SH may be easier to engage with than CBT-SH. The trial proved feasible: recruitment and retention were good – of the 55 participants referred, 72% (40) were randomised, recruitment was completed within the expected timeframe and study retention was 78%.

Following our successful pilot work we now plan to conduct a definitive RCT to compare MBCT-SH with CBT-SH for people experiencing mild to moderate depression. This is the purpose of the current study.

4.2 Research question and hypotheses

Our primary hypothesis in this current study, **Study 3**, is that supported MBCT-SH, in comparison to supported CBT-SH, will lead to greater reductions in depressive symptom severity (PHQ-9[12]) from baseline to post-intervention.

Secondary hypotheses are:

(2) MBCT-SH in comparison to CBT-SH will lead to greater reduction in depressive symptom severity from baseline to six-months follow-up.

(3) A greater proportion of MBCT-SH participants will be in the non-clinical range for depressive symptoms than CBT-SH participants at post-intervention (i.e. remission) and six-months follow-up (i.e. recovery).

(4) MBCT-SH in comparison to CBT-SH will lead to greater improvements in mindfulness, generalised anxiety, work and social adjustment and wellbeing from baseline to post-intervention and from baseline to six-months follow-up.

(5) Treatment completion rates will be higher for MBCT-SH than CBT-SH.

(6) Depressive symptom severity outcomes will be mediated by treatment completion.

(7) MBCT-SH will be cost-effective in comparison to CBT-SH at follow-up.

A qualitative component is employed to close the evidence gap concerning reasons for treatment non-completion at Step 2 by identifying facilitators and barriers to treatment completion in both arms.

5 Patient and Public Involvement (PPI)

5.1 PPI to date

Our pilot RCT provided the foundation for the proposed study. Laura Lea (LL) was involved in the design of the pilot RCT from the outset. During the pilot RCT LL chaired the Lived Experience Advisory Panel (LEAP) that contributed to the development of participant materials and recruitment and retention strategies. Two members of the LEAP team from the pilot RCT suggested greater attention should be given to the qualitative element of this proposed study. A significant qualitative component is now included in this submission.

For the proposed study we successfully applied for a PPI grant from RDS South East in November 2015 which funded a consultation with four participants from the pilot RCT along with two members of the original LEAP.

As a result of these consultations the following changes to the proposed study were made:

- The qualitative element of the study has been expanded in order to explore participants' experiences of their allocated intervention beyond depressive symptom reduction.

- A measure of wellbeing has been added following feedback that outcomes beyond depressive symptom severity should be explored.

- Adjustments have been made to arranging assessment appointments so that they are at a time and place convenient to the participant.

- An additional person with lived experience has been appointed to the Trial Steering Committee (TSC).

- Training needs for people with lived experience who wish to take part in the LEAP or sit on the TSC have been identified (see below for details).

- A record of impact of PPI will be kept.

- Time and resources have been allocated and a journal identified (Mental Health Today) to enable members of the LEAP to disseminate findings beyond peer reviewed journals.

- The number of LEAP meetings have been increased from four to seven to support study monitoring and dissemination.

- A meeting with the LEAP panel will be arranged to present qualitative findings and to seek feedback prior to finalising the thematic map.

- A Deliberative Workshop with participants who took part in the qualitative interviews will be hosted in order to present the qualitative findings and to prioritise the importance of themes prior to dissemination.

5.2 *Current PPI*

In this study the Lived Experience Advisory Panel (LEAP) will consist of six people, including participants from our previous pilot RCT. It will be facilitated by LL who has extensive experience of facilitating consultations with members of the public involved in research. Training and support for the LEAP will be provided by LL and CS. CS will offer half a day to familiarise the LEAP with the study design, delivery and dissemination processes. LL will offer half a day on familiarisation with involvement in research. Members will be offered a role description, details of expectations of the role, a complaints process and expenses and payment for participation. Meetings will be a mix of face-to-face and virtual, including email correspondence, to acknowledge the availability and working preferences of LEAP members.

The LEAP will meet on seven occasions:

- Once prior to submission for NHS ethics approvals and once following feedback from the REC/HRA to feedback on any changes that may be required. The LEAP will contribute to the refinement of recruitment and retention materials that were originally developed in our pilot study. The LEAP will also advise on training

for research assistants to ensure inclusion of the changes advised in the PPI consultation.

- Twice during recruitment to comment on processes and progress and identify and advice on any issues relating to delivery of the project.
- Three times at the end of the study to comment on qualitative findings (one meeting) and on dissemination (two meetings) – see Dissemination section for details.

Two members of the wider and well-established PPI research group in Sussex Partnership who are independent of the current study (i.e. not LEAP members on the current or previous study) will be recruited to sit on the Trial Steering Committee. This will enable independent monitoring and oversight of the project from a lived experience perspective.

Qualitative interviews will be conducted using the Change Interview[26] in order to ascertain facilitators and barriers to treatment completion for each intervention, with questions added to the end of interview following advice from the PPI consultation panel. These questions enquire about participants' experiences of their allocated intervention beyond depressive symptom change.

6 Methods/Design

6.1 *Design*

This is a parallel groups, superiority pragmatic RCT with 1:1 allocation to MBCT-SH or CBT-SH with blinded assessments at all time points. Participants will be blind to the hypothesised direction of effects.

The study includes qualitative evaluation of participants' experiences of both self-help interventions with a focus on better understanding barriers and facilitators to engaging in Step 2 interventions in IAPT.

Four hundred and ten people meeting eligibility criteria for major depressive disorder or mixed anxiety and depression will be randomly allocated to receive MBCT-SH or CBT-SH, along with six sessions of support from a psychological wellbeing practitioner. Participants will complete measures at baseline, 16 weeks post-randomisation (post-intervention) and 42 weeks post randomisation (6-months follow-up). In addition, 40 participants will be interviewed about their experiences.

6.2 *Participants*

Participants will be recruited through four IAPT services: (1) Health in Mind in East Sussex, (2) Brighton and Hove Wellbeing Service, (3) Sussex Community Trust, and (4) Southwark IAPT service.

6.2.1 Inclusion/exclusion criteria

Inclusion criteria are that participants will:
(1) be aged 18 years or over;
(2) meet diagnostic criteria on the revised Clinical Interview Schedule (CIS-R)[27] for a primary diagnosis of a depressive episode, mixed anxiety and depression, or non-specified mild neurotic disorder at their eligibility screening assessment;
(3) score 10 or more on the PHQ-9[21] at their eligibility screening assessment (the cut-off for a major depressive episode); and
(4) have sufficient literacy skills to read and understand the self-help materials

Exclusion criteria are that if people:
(1) have severe symptoms of depression at their eligibility screening assessment (a score 20 or more on the PHQ-9);
(2) score of 4 on the CIS-R suicidality scale
(3) express a strong preference (5/5) for one intervention over the other on the Treatment Preference Question such that if randomised to the non-preferred intervention they would be likely to drop out of the intervention.

6.3 Intervention protocols

6.3.1 MBCT Self-Help

The MBCT-SH workbook 'The Mindful Way Workbook'[30], written for clinical populations, presents MBCT as a self-help package. MBCT-SH participants will be given the workbook and will be asked to guide themselves through the self-help course within a 16 week time period (the time period determined in our pilot). As is routine at Step 2, participants will be offered six PWP sessions to answer questions and provide encouragement.

PWPs currently train in CBT-SH. To match training between arms we will offer PWP training in MBCT-SH. The PWP training package was developed and successfully implemented in our pilot RCT. It has since been refined in consultation with PWPs in our pilot study and with co-applicant Dr Rebecca Crane, an international leader in MBCT therapist training.

The training involves PWPs: (1) attending an MBCT group as a participant and guiding themselves through the MBCT-SH workbook, or, completing the MBCT course using the workbook as a guide with weekly telephone support offered by Clara Strauss or Fergal Jones, and (2) attending a two-day experiential mindfulness inquiry skills workshop (facilitated by Dr Clara Strauss, Dr Fergal Jones and Ms Laura Lea). As is standard in delivering mindfulness interventions, PWPs will be encouraged to maintain their own personal mindfulness practice and this will be recorded using the PWP Mindfulness Practice Record.

Weekly MBCT-SH supervision for PWPs will be provided by Drs Strauss and Jones, accredited MBCT therapists. Drs Strauss and Jones will in turn receive monthly supervision from Dr Crane.

6.3.2 CBT Self-help

The CBT-SH workbook 'Overcoming Low Mood and Depression'[31] has evidence demonstrating its effectiveness in reducing depression symptom severity[32]. This workbook is routinely used at Step 2 in IAPT and was the comparator in our pilot study.

Matched to the MBCT-SH condition, participants allocated to CBT-SH will be given a copy of their workbook and will be encouraged to guide themselves through within 16 weeks alongside six PWP sessions to answer questions and provide encouragement.

Weekly CBT-SH supervision for PWPs will be provided by Drs Strauss and Jones, both of whom are CBT therapists. Drs Strauss and Jones will receive monthly supervision for the CBT-SH arm from co-applicant Dr Kate Cavanagh, an international leader in research evaluating supported CBT self-help interventions.

6.3.3 Intervention Fidelity and Adherence

The same PWPs will deliver both interventions in order to minimise therapist effects as some PWPs achieve substantially better outcomes than others. In order to minimise therapeutic drift and therapy contamination the PWP protocols are detailed and there will be weekly supervision of PWPs. Where possible, PWP sessions will be audio recorded with each PWP asked to submit at least one complete audio-recorded case from each arm of the trial which will be rated for adherence. Where possible, recordings will be listened to and rated for fidelity as the study progresses in order to ensure that any breaches to fidelity can be addressed in a timely manner and in order to provide supervision feedback to PWPs. Audio recordings will be stored securely on password protected NHS computers.

Participants will be prompted each week by the member of the research team to complete weekly diaries (online/paper versions), adapted from the diaries used in our pilot RCT, to record amount of workbook read and time spent engaged in intervention tasks

6.4 Primary & Secondary Outcome Measures

The research team in collaboration with the PPI consultation panel have carefully considered measures to maximise information obtained whilst minimising participant burden and ensuring good study retention.

Measures and tools are attached.

Measures and tools will be used at baseline (T0), 16 weeks post-randomisation (T1) and/or 42 weeks post-randomisation (T2) as indicated.

6.4.1 Demographic Information

A questionnaire will be completed to collect demographic information. We will also ask participating IAPT services to provide summary demographic data, in order to compare the demographic profile of the study participants to see if they are representative of the recruiting service. This data will be high level (e.g. percentage of women/men accessing the IAPT service, mean age of service users) and will not include any identifying information.

6.4.2 Diagnosis

The Clinical Interview Schedule (CIS-R)[27] will be conducted at the eligibility screening assessment to ascertain diagnostic status. The CIS-R is routinely used in primary care mental health and IAPT research[1] and has been validated for telephone completion[33].

6.4.3 Primary outcome measure

Depression symptom severity (PHQ-9)[21]. The PHQ-9 is a 9-item self-report measure of depression symptom severity used in all IAPT services. Items are rated on a four-point scale. Scores under 10 are considered sub-clinical, 10-14 mild, 15-19 moderate and 20+ severe (eligibility screening assessment, T0, T1, T2, plus at each PWP support session).

6.4.4 Secondary outcome measures

- *Generalised anxiety (GAD-7)*[34]. This is a 7-item measure of generalised anxiety used in IAPT. Items are rated on a 4-point scale and the measure has excellent psychometric properties[34] (T0, T1, T2, plus at each PWP support session).

- *Wellbeing (SWEMWS)*[35]*.* The short version of the Warwick Edinburgh Mental Wellbeing Scale consists of 7 questions rated on a 5-point scale designed to measure wellbeing. The scale has good psychometric properties and is used widely[36]. This measure was added following advice from the PPI consultation panel (T0, T1, T2).

- *Functioning (WSAS)*[37]*.* The Work and Social Adjustment Scale (WSAS) is a 5-item measure of daily occupational and social functioning that is used routinely in IAPT (T0, T1, T2, plus at each PWP support session).

- *Mindfulness (FFMQ-15)*[38]. Mindfulness will be measured using 15-item version of the Five-Facet Mindfulness Questionnaire. This has excellent psychometric properties and is sensitive to change following MBCT[38] (T0, T1, T2).

6.4.5 Intervention Evaluation Measures and Tools

- *Intervention expectation form*. This will be used to assess expectation of benefit and treatment credibility (T0)
- *Lasting effects questionnaire*. This will be used to ask participants about any lasting negative effects of their allocated intervention (T2)
- *PWP rating scale*. This will be used for participants to rate the quality/helpfulness of the support sessions between the participant and their PWP (T1)
- *Weekly diaries*. These record the extent to which participants are engaging with the self-help course each week during the self-help course (weekly between T0 and T1)
- *Engagement questionnaire (end of treatment)*. This records the extent to which participants engaged with the self-help course during the entire course of the intervention (T1).
- *Engagement questionnaire (follow-up)*. This records the extent to which participants continued to engage with the self-help course following the end of the intervention (T2)
- *Session attendance*. Number of PWP sessions attended (0-6) and duration of each support session.
- *Treatment completion*. This is defined as attending at least three PWP sessions.

6.4.6 Health economic measures

- *Service use*. A self-report version of the Adult Service Use Schedule (ADSUS), adapted from previous depression trials[39], will be used to collect data on resource used to estimate costs and will take the health and social services perspective preferred by NICE[40] (T0, T1, T2).
- *Health-related quality of life (EQ-5D-5L)*[41]*.* The EQ-5D is a five-dimension, generic, preference-based measure of health-related quality of life covering mobility, self-care, usual activities, pain/discomfort and anxiety/depression. We will use the recently developed five level version, to maximise sensitivity[42] (T0, T1, T2).

6.4.7 Qualitative evaluation

The aim is to ascertain facilitators and barriers to treatment completion for each intervention given the well-established problem with drop-out from IAPT.

Fourty telephone interviews will be conducted with twelve participants interviewed from each of the two groups; (1) MBCT-SH intervention completers, (2) (2) CBT-SH intervention completers,. In addition, 16 telephone interviews will be conducted with intervention non-completers, 8 from each intervention arm. This strategy will ensure that participants are interviewed in equal numbers from each intervention arm. The sample size is sufficient in relation to our aim given the relatively homogenous sample (i.e. clients with the same/similar diagnoses recruited from IAPT services), meaning that fewer than 10% of the study sample need to agree to an interview. Telephone interviews will reduce participant burden and participants will be paid for their interview, over and above payments to undertake quantitative assessments. Interviews will take place following the post-intervention assessment in order to accurately capture participants' experiences.

The Change Interview[26] will be used. This is a widely-used semi-structured interview designed to explore participants' experiences of psychological interventions and has been successfully used in previous studies by members our team. Questions about participants' experiences of effects of their allocated intervention on personally-relevant outcomes (i.e. not necessarily restricted to depressive symptoms) have been added at the end of the interview following advice from the PPI consultation panel.

The Lived Experience Consultant (LL) will conduct the telephone interviews. Ms Lea has lived experience of depression and has been a core member of the research team since its inception. She will be well supported in undertaking the interviews by the CI and by a costed lived experience supervisor.

6.5 ***Recruitment and consent methods***

Recruitment and retention is feasible as shown in our pilot RCT[23] of essentially the same design where we recruited 40 participants in one of the five IAPT services in six months with a 0.4 FTE research assistant (RA). In the 2013-14 financial year (the latest year with available figures), this IAPT service received 4680 referrals. Based on IAPT figures[3], around 55% of these (around 2500 people) would be presenting with depression or mixed anxiety and depression. We are seeking to recruit approximately 140 participants in this site over eight months, or 8% of those eligible. Our pilot study was based in an IAPT service serving a large, rural area where recruitment is likely to have been more challenging than in urban settings. Basing these estimates on our pilot findings may underestimate rather than overestimate recruitment in this definitive trial.

Particular effort will be given to retain participants, including intervention drop-outs. Evidence-based data collection strategies identified in the recent Cochrane review[28] will be employed, including paying participants for all assessments. In addition,

based on participant feedback from our pilot RCT, data will be collected online (with postal option) in order to minimise participant burden.

Adults experiencing depression or anxiety problems can refer themselves to their local IAPT service or be referred by their GP or another health professional. Participants will be recruited through IAPT services following initial assessment.

This study will also be advertised locally in local newspapers, on local radio, websites, social media and on leaflets and flyers in community settings (e.g. GP surgeries, libraries, community settings). GPs will also be asked to offer the study to suitable patients during appointments using the study leaflet. GPs, statutory and third sector mental health support organisations (e.g. local mental health support organisations, university counselling services) will be asked to send a cover letter and leaflet to people on their caseloads experiencing depression. The adverts will provide brief information about the study and will direct potential participants to the research team where they can find out more about the study and, if interested in taking part, complete the eligibility screening assessment (CIS-R and PHQ-9). If eligible, potential participants will receive an initial standard IAPT assessment and they will then be offered a baseline assessment by a study member of the research team.

If not meeting eligibility criteria for the study at the eligibility screening assessment, potential participants will be signposted to appropriate sources of support which will include usual care in the IAPT service where relevant.

The recruitment process is summarised as follows:

1. Initial Eligibility Screening Assessment. Following referral, potential participants will complete an initial eligibility screening assessment with a member of the research team. This includes completion of the CIS-R and PHQ-9. Potential participants (i.e. those scoring 10-19 inclusive on the PHQ-9 and meeting diagnostic criteria for major depression, mixed anxiety and depression or non-specified mild neurotic disorder on the CIS-R) will be offered the study. Potential participants not meeting eligibility criteria will not be offered the study and will be signposted to appropriate sources of support including usual care in the IAPT service where relevant. The data from these screen-failed participants will be anonymised and recorded for information purposes to help guide the success of the study.

2. Participant Information Sheet (PIS). If eligible and interested in the study, potential participants will be given a copy of the Participant Information Sheet. The PIS will be available online and a copy will also be sent to potential participants.

3. Consent. Potential participants will speak with a member of the research team (or with a CRN-funded researcher) to answer questions about the study and to complete the consent form (if appropriate). As assessment and intervention is typically offered by telephone at Step 2 in IAPT, participants will be offered the

choice of having the consent meeting by phone or in person. The consent form will be presented online for participants to complete during the meeting (with hard copy option available). Potential participants not wanting to take part in the study will be signposted to appropriate sources of supporting including usual care in the IAPT service where relevant.

6.6 ***Assessment process***

Assessments will be conducted at baseline (T0), 16 weeks post-randomisation (T1) and 42 weeks post-randomisation (T2). A member of the research team involved with post-intervention and follow-up assessments will be blind to group allocation. In addition participants will be asked to complete weekly diaries to record intervention usage and routine IAPT measures (see below) will be recorded at the six PWP support sessions.

6.7 ***Procedure***

Once the participant has consented to participate in the trial, the participant will complete the full set of baseline measures with a RA present in person or by phone. Measures will be completed online or on paper, depending on participant preference. Participants who do not meet eligibility criteria at the baseline assessment will be referred back to the person who conducted their initial assessment for usual care to be offered by the service.

At the end of the baseline assessment, eligible participants will be randomised to either the MBCT-SH or CBT-SH arm. Participants will then be given their allocated self-help workbook.

Participants will then guide themselves through their allocated intervention over the 8 week course with six PWP support sessions. A maximum of 16 weeks is given for the intervention period to allow participants to complete their allocated 8-week course to take account of breaks for holidays, sickness etc. Each PWP support sessions will typically be offered by phone (as is usual in IAPT) but may also be offered face-to-face or by email. This mirrors the usual way in which interventions are offered at Step 2 in IAPT – i.e. offering a self-help workbook alongside a limited number of PWP support sessions.

Participants will complete measures online (with a postal option) at 16 weeks post-randomisation (post-intervention) and 42 weeks post-randomisation (6-month follow-up).

40 participants will be invited to take part in the qualitative Change Interview after their post-intervention quantitative assessment is completed. Participants will be interviewed on a first come, first served basis with 12 participants interviewed in each group: (1) MBCT-SH intervention completers, (2) CBT-SH intervention completers. Study completers are defined as those who have attended at least three PWP sessions. We also plan to interview 8 non-completers from each arm.

Payments by £20 gift vouchers will be made to participants for completing each assessment, including the qualitative interview.

6.8 Randomisation process & allocation concealment

Randomisation will be stratified by centre and PHQ-9 score (mild or moderate) using random block length.

Eligible participants will be randomly allocated using the Sealed Envelope[29] online service. The team statistician will use Sealed Envelope to set up and test the randomisation procedure incorporating stratification by site and PHQ-9 severity category (mild or moderate) using random block length and 1:1 allocation. The statistician will not have any further involvement in the randomisation process. The RA will randomise participants by completing the online form with participant's details. This will immediately show whether the participant is assigned to the MBCT-SH or CBT-SH arm and participants will be given their self-help workbook. Participants will not be told the hypotheses in relation to the arm they have been randomised to.

7 Data Management & Analysis

7.1 Summary of the Types of Data

Qualitative data will be generated from:

- 40 telephone interviews; these will be audio recorded and stored as MP3 files and transcribed into word documents,
- Where possible, PWP sessions will be audio recorded and stored as MP3 files.

Quantitative data will be generated from:

- The completion of questionnaires at baseline, 16 weeks and 42 weeks,
- Sessional attendance data - engagement in workbook practices/activities

7.2 Research Variables Form (RVF)

Type of data	Variable name	Outcomes/units	Source/Any Instructions
Diagnostic	CIS-R	Diagnosis	Used for inclusion/exclusion criteria
Quantitative	PHQ-9<br>GAD-7<br>WSAS,<br>FFMQ-15<br><br>SWEMWS<br>EQ-5D-5L	Total<br>Total<br>Total<br>Total and subscale scores<br>Total<br>Subscale scores converted into	NB the SWEMWBS has a conversion tool at http://www2.warwick.ac.uk/fac/med/research/platform/wemwbs/researchers/guidance/swemwbs_raw_score_to_metric_score_conversion_table.pdf

	ADSUS<br>Therapy Expectation Form	utility values<br>Total<br>Total	Therapy expectation used at baseline only
Quantitative	Demographics	DoB,<br>Gender ,<br>Education,<br>Employment status,<br>Marital status,<br>No. of children,<br>First language,<br>Ethnicity,<br>Previous therapy	
Quantitative	Session attendance, sessional PHQ-9, GAD-7 and WSAS data.		These measures are collected by PWPs at each support session in line with standard IAPT practice.<br><br>Number sessions 0-6
Quantitative	Intervention engagement diaries and questionnaires		Weekly diaries during intervention period plus post-intervention and follow-up questionnaires
Qualitative	Change Interview		
Quantitative / Qualitative	Lasting Effect Questionnaire	Descriptive	Follow-up only
Quantitative	PWP Rating Scale		At post-intervention only

7.3 ***Sample size & Power calculations***

The sample size was based on detecting a between-group effect size of 0.36 based on the difference between the reported between-group effect of CBT-SH (0.42)[43] and the reported between-group effect of MBCT-SH (0.78)[44]. Recruiting 205 patients into each arm would provide 90% power to detect a between-group difference of 0.36 with a 5% alpha and a two-sided t-test whilst allowing for 20% attrition at post-intervention (as found in the pilot RCT); therefore a total sample size of 410 will be required.

In our pilot RCT of the same design we recruited 40 participants in one of the five sites in six months with a 40% FTE RA. This equates to recruiting 466 participants in the eight month recruitment window of the proposed study. This means that it is

feasible to recruit to the 410 participant target whilst allowing for an up to 10% shortfall in expected recruitment rates.

7.4 Planned data analysis

Quantitative analyses will be based on the intention-to-treat approach, where participants are analysed as per their randomisation allocation regardless of treatment received, and in addition per protocol analysis will be conducted for those participants receiving an adequate dose of their allocated intervention (defined as completing at least 50% of their allocated intervention). Participant flow through the study will be reported in line with the Consolidated Standards of Reporting Trials (CONSORT) 2010 statement[45]. A descriptive summary of all variables will be provided by group and time point. Unstandardised effect sizes for the primary outcome and secondary outcomes will be estimated using linear mixed models with treatment group (MBCT-SH vs CBT-SH), time (16 and 42 weeks) and a treatment group x time interaction entered as fixed factors; site, baseline PHQ-9 and baseline value of the outcome will be entered as covariates. Individual participants will be included in the analysis as random effects. Contrasts will be used as appropriate to estimate effects at different time points. A non-significant group x time interaction will imply common treatment effects at each time point. Standardised (Cohen's *d*) effect sizes for each outcome will be calculated by dividing the between-group unstandardized effect by the baseline pooled standard deviation. 95% confidence intervals will be calculated for all estimates. Missing data will be assessed and dealt with using appropriate methodology e.g. multiple imputation and a sensitivity analysis will be carried out. Group differences in dichotomous outcomes at the different time points will be analysed in a similar way but using multilevel logistic regression models and baseline PHQ-9 scores.

Economic evaluation will take the NHS/personal social services perspective preferred by NICE, including use of all hospital, community health and social services. Data on PWP contacts in the MBCT-SH and CBT-SH arms will be collected from PWP records. Data on indirect time, including preparation and supervision, will be collected directly from the PWPs. Data on use of other services will be collected using the Adult Service Use Schedule (AD-SUS), as described above.

The cost of the two interventions will be directly calculated from salaries using a micro-costing[46]. National UK unit costs will be applied to medication, hospital contacts and community health and social services, taken from various sources including the Personal Social Services Research Unit (PSSRU) Unit Costs of Health and Social Care, NHS Reference Costs and the British National Formulary.

Differences in mean costs will be analysed using standard parametric t-tests with the validity of results confirmed using bias-corrected, nonparametric bootstrapping (repeat re-sampling)[47]. Despite the skewed nature of cost data, this approach is recommended to enable inferences to be made about the arithmetic mean[48]. Cost-effectiveness will be assessed at six-months follow-up through the calculation of incremental cost-effectiveness ratios (ICER) and will be explored in terms of quality

adjusted life years calculated using the EQ-5D-5L measure of health-related quality of life. Uncertainty around the cost and effectiveness estimates will be represented by cost-effectiveness acceptability curves[49].

Qualitative data will be analysed by Ms Lea, Dr Rosten and Dr Strauss using Thematic Analysis[50] in order to identify facilitators and barriers to treatment completion for each intervention. Dr de Visser will oversee and supervise this aspect of the study.

7.5 Dummy results tables

Table 1 Descriptive summary for demographic variables

	Treatment<br>N		Control<br>N		Total<br>N	
Gender:						
Male	n	%	n	%	n	%
Female	n	%	n	%	n	%
Identify as another term	n	%	n	%	n	%
Whether Transgender:						
Yes	n	%	n	%	n	%
No	n	%	n	%	n	%
Age (years) Median /range						
Ethnicity :						
White British	n	%	n	%	n	%
BME	n	%	n	%	n	%
Sexual Orientation						
Heterosexual	n	%	n	%	n	%
Gay	n	%	n	%	n	%
Bisexual	n	%	n	%	n	%
Lesbian	n	%	n	%	n	%
Identify as another term	n	%	n	%	n	%
Marital Status:						

Single	n	%	n	%	n	%
Married/Civil Partnership	n	%	n	%	n	%
Living with Partner/Cohabiting/Long Term relationship	n	%	n	%	n	%
Divorced/Separated	n	%	n	%	n	%
Widowed	n	%	n	%	n	%
Employment Status:						
Unemployed	n	%	n	%	n	%
Employed	n	%	n	%	n	%
Home maker/Carer	n	%	n	%	n	%
Student	n	%	n	%	n	%
Retired	n	%	n	%	n	%
Other	n	%	n	%	n	%
Education Level:						
None	n	%	n	%	n	%
GCSE or equivalent	n	%	n	%	n	%
A’ Level or equivalent	n	%	n	%	n	%
Undergraduate or equivalent	n	%	n	%	n	%
Post graduate or equivalent	n	%	n	%	n	%
Symptom Severity: CIS-R						
Mild	n	%	n	%	n	%
Moderate	n	%	n	%	n	%

Severere	n	%	n	%	n	%
Therapy Expectation:						
Expectation of benefit	Mean (sd)		Mean (sd)		Mean (sd)	
Credibility	Mean (sd)		Mean (sd)		Mean (sd)	

Table 2 Descriptive Summary of Baseline outcomes

MBCT Self-help participants	*N*	*Mean*	*Std. Deviation*	*Median*	*Min-Max*	*IQR*
PHQ-9						
SWEMWS						
GAD-7						
FFMQ-15						
WSAS						

Table 3a Descriptive summary of adherence (treatment completion) rates

Treatment	Adherence Yes = 1	Adherence No = 0	*Total*		*Observed proportion adhering*	*Anticipated proportion of successes*	*Odds Ratio*
MBCT	a	b	m		a/m	p1	
CBT	c	d	n		b/n	p2	
TOTAL	r	s	N				

Table 3b Descriptive Session Attendance

Number of sessions attended	Counts	Proportion
1	n	%
2	n	%
3	n	%
4	n	%
	mean	

Table 3c Summary of PWP ratings

Sub Score:	N	Mean (SD)	range
Relationship	xx	xx	xx
Goal	xx	xx	xx
Approach	xx	xx	xx
Overall Score	xx	xx	xx

Table 4 Counts of Valid Cases and Missing Values at each time point for the key variables, by group and overall: N

Variable	Baseline		Post Group		Follow-up	
	Valid cases	Missing cases (%)	Valid cases	Missing cases (%)	Valid cases	Missing Cases (%)
PHQ-9						
SWEMWS						
FFMQ-SF						
GAD-7						
WSAS						

Table 5 & 6 Descriptive summary of post-changes: differences; comparison of change scores in the treatment and control group from Baseline to post scores/Baseline to follow-up

Outcome	What change means improve-ment +/-	N	Treatment		Control		Pooled SD	Difference (95% CI)	Effect size (d)
			Mean change	SD	Mean change	SD			
PHQ-9	-								
SWEMWS	+								
FFMQ-SF	+								
GAD-7	-								
WSAS	-								

Table 7 & 8 Effect size results from the Linear Model unadjusted and adjusted for missingness

Outcome	Unadjusted / adjusted				
	Estima te	SE	95% CI	P	Cohen’s d
PHQ-9					
SWEMWS					
FFMQ-SF					
GAD-7					
WSAS					

Table 9 Summary of adverse events

Adverse event	Treatment	Control
Event 1	N	
Event2…	N	
Total	N adverse events	N_adverse events

7.6 *Data collection, entering, coding and checking process*

- Anna-Marie Jones will be the data manger and will oversee all data management.
- The project Research Assistant will be responsible for all data collection and this will be overseen by the data manager. Any training required for the RA so that they can use the data collection tools will be carried out by the PI & the data manager as required. The RA will have a 'Research Assistant Data Management Plan'.
- The RA will develop the electronic version of the data collection booklet using Qualtrics and this will be overseen by the data manager. At the point of collection, if a wifi signal is available and the participant is willing, data will be collected via an electronic tablet. The electronic version will be tested out among the team before it goes live.
- The RA will develop the paper version of the data collection booklet (DCB) this will be overseen by the data manager. Any data collected on paper will be entered into Qualtrics by the RA when they return back to their base (Sussex Education Centre). Any mistakes made on the form should be cleanly crossed out and rewritten. The person making the amendment should initial and date the correction. The error should not be overwritten. Correction fluid must not be used.
- The RA will code the data and produce a code book for the data. This will be checked by the data manager.
- Baseline checks: The first 5 data booklets entered from paper on to the electronic system will be checked by the data manager; out of the next 20 a random sample of 5 will be double entered by an RA independent to the study. The double entry will be checked by the data manager. SPFT data monitoring checks will then be carried out when N= 120, at N=220 at N=320 and when all the data is entered.
- Follow-up data will be collected using Qualtrics, participants will be asked to enter the data online. They will be emailed a link to the questionnaire. Paper copies will be sent in self-addressed envelope if specifically requested.
- Follow-up data checks will be carried out at N=5, N=20, at N=120 and N=320 and when the data is all entered

7.7 *Missing data policy*

We aim to minimise missing data at the point of collection. The Qualtrics software used to collect data will automatically flag any unanswered questions, giving participants to chance to answer these. If a participant would prefer not to answer a question they can leave it unanswered for a second time and the software will proceed onto the next page.

At the point of coding the following missing data values will be used.
666 = Dropped out

888 = Not applicable
999 = system missing/missing from questionnaire
1/1/999 = missing date

At the point of analysis: Data will be summarised to look at patterns of missingness. Missing data will replaced using multiple imputation as appropriate.

7.8 Data custodian and data ownership

Name of data custodian & owner: Dr Clara Strauss, Sussex Partnership NHS Foundation Trust, clara.strauss@nhs.net

7.9 Data quality and Standards

The research team adhere to the good practice and standards principles which are set out in the Sussex Partnership Policy for Data Protection, Security and Confidentiality 2013. This policy reflects the recommendations from current legislation, including The Caldicott Report (1997), the British Standard (ISO IEC 27002) for Information Security, the Data Protection Act, 1998 and the Sussex Partnership Foundation Trust Research Policy 2012.

All research will be carried out under the above standards and will be reviewed by an NHS Ethics Committee and given approval by the R&D Department under the NHS Research Governance Framework 2005.

All members of the research team and any other individuals from collaborating Trusts or Universities involved in collecting, inputting, processing, using and sharing data will have had Information Governance Training.

Data management will be a standard item on the agenda for both research team and steering group meetings.

7.10 Data security

Participant contact details will be stored on password protected, NHS computers that cannot be accessed by anyone outside of the research team. Consent forms will be stored in locked filing cabinets on NHS premises. Assessment booklets will be pseudoanonymised using a unique subject identifier. The subject identification log linking the unique identifier with the participant’s identifable information will be kept securely by the principal investigator at each research site. The study SPSS file will not contain identifiable information, the unique study identifiers will be used.

Electronic files will be stored on password protected NHS computers. Only non-identifiable information will be included on electronic files aside from an Excel spreadsheet which will link participant names and contact details with their non-identifiable code.

Members of the research team will have access to the participant's personal data collected for the purposes of the study once they have received consent from the participant.

Monitors or auditors from regulatory authorities or the sponsor organisation may have access to the participant's personal data during the study for purposes relating to their taking part in the study and consent will be sought for this from the participant.

Data generated from the study will be analysed by members of the research team. Quantitative data will be analysed using SPSS on password protected NHS computers or on password protected university computers. Qualitative data will be fully anonymised and stored as Word documents on password protected NHS computers or on password protected university computers.

The research data generated from the study will be stored for at least 5 years from the end of the study. The Investigator Site File will be archived in accordance with each local research sites archiving policies. The Trial Master File will be archived in accordance with the Brighton & Sussex Clinical Trial Unit archiving procedures. The sponsor will confirm when archiving procedures can commence and when the study documents can be destroyed.

7.11 Data sharing

Consent will be sought from each participant to allow anonymised sharing of research data for future research in this area.

8 Project management

Dr Strauss has led a number of RCTs of psychological interventions in the NHS as CI and site lead.

8.1 Trial Management

Dr Strauss will chair research team meetings where adherence to the research protocol will be checked and problems identified and addressed.

The Trial Manager, provided by the Brighton and Sussex Clinical Trial Unit, will help to manage the study, meeting weekly with Dr Strauss.

Dr Strauss will offer weekly supervision to the RAs.

8.2 Data Management

Ms Jones will oversee data collection/management and the randomisation process.

8.3 *Financial Management*

Ms Taffy Bakasa (Lead Governance Officer, Sussex Partnership) will manage study finances.

8.4 *Trial Oversight*

A Trial Steering Committee (TSC) and Data Monitoring and Ethics Committee (DMEC) will be established according to MRC guidelines[51].

The TSC will be chaired by Professor Willem Kuyken (Director of Oxford Mindfulness Centre, University of Oxford) with two independent experts (including Professor Jörg Huber, Academic Site Lead RDS Sussex) and two independent PPI members.

The DMEC will be chaired by Dr Mark Kelson (Research Fellow in Statistics, South East Wales Trials Unit) with two independent experts (including Dr Catherine Crane, Senior Post-doctoral Research Psychologist, Department of Psychiatry, University of Oxford) and an independent PPI member.

9 Ethical considerations

The proposed study is essentially a larger version of our pilot RCT[23] which was awarded NHS ethics approval by the NRES Committee South East Coast-Surrey (ref: 13-LO-1769) and R&D approval was subsequently awarded by Sussex Partnership. The ethical and governance issues for the proposed study are not different to those of the pilot RCT.

10 Data monitoring for harm

ICH GCP requires that both investigators and sponsors follow specific procedures when notifying and reporting adverse events/reactions in research studies. These procedures are described in this section of the protocol.

Table 1: Definitions for adverse events and reactions

Term	Definition
**Adverse Event (AE)**	Any untoward medical occurrence in a patient treated on a study protocol, which does not necessarily have a causal relationship with a study intervention. An AE can therefore be any unfavourable and unintended sign, symptom or disease temporally associated with the use of a study intervention, whether or not related to that study treatment.
**Adverse Reaction (AR)**	All untoward and unintended responses related to a study intervention. A causal relationship between a study intervention and an adverse event is at least a reasonable possibility, i.e. the relationship cannot be ruled out as

	there is evidence or arguments to suggest a causal relationship.
**Unexpected Adverse Reaction (UAR)**	An adverse reaction, the nature or severity of which is not consistent with the information about the trial intervention.
**Serious Adverse Event (SAE) or Serious Adverse Reaction (SAR) or Suspected Unexpected Serious Adverse Reaction (SUSAR)**	Respectively any adverse event, adverse reaction or unexpected adverse reaction that:<br>• results in death<br>• is life-threatening*<br>• requires inpatient hospitalisation or prolongation of existing hospitalisation**<br>• results in persistent or significant disability or incapacity<br>• consists of a congenital anomaly or birth defect

For the LIGHTMind 2 study, we have amended the definitions of Serious slightly to make it more appropriate to the type of study, as follows:

- Results in death;
- Is life-threatening (including self-harm requiring hospitalization);
- Requires inpatient hospitalisation, inc. A&E visit or prolongation of existing hospitalisation;
- Results in persistent or significant disability or incapacity

Life-threatening (*), in the definition of 'serious', refers to an event in which the patient was at risk of death at the time of the event; it does not refer to an event which hypothetically might have caused death if it were more severe.

Hospitalisation (**) is defined as an inpatient admission, regardless of length of stay, even if the hospitalisation is a precautionary measure for continued observation. Hospitalisations for a pre-existing condition (including elective procedures that have not worsened) do not constitute an SAE.

Clinical judgement should be exercised in deciding whether an AE/AR is serious in other situations. Important AE/ARs that are not immediately life-threatening or do not result in death or hospitalisation but may jeopardise the subject or may require intervention to prevent one of the other outcomes listed in the definition above, should also be considered serious.

10.1 *Investigator Assessment*

(a) Seriousness
When an AE/AR occurs, a local investigator responsible for the care of the patient or delegated the duty of assessing AEs must first assess whether the event is serious using the definition given in Table 1.

If the event is classified as serious then this must be reported to the Brighton & Sussex Clinical Trial Unit immediately via email at safety@bsuh.nhs,uk, on the SAE reporting form, at least within 24 hours of being made aware of the event.

(b) Causality
The local investigator must also assess the causality of all serious events in relation to the trial intervention using the definitions in Table 2. There are 5 categories: unrelated, unlikely, possible, probable and definitely related. If the causality assessment is unrelated or unlikely to be related the event is classified as an SAE or AE. If the causality is assessed as either possible, probable or definitely related then the event is classified as a SAR or AR.

Table 2. Definitions of causality for adverse events

Relationship	Description	Event Type
**Unrelated**	There is no evidence of any causal relationship	SAE or AE
**Unlikely**	There is little evidence to suggest there is a causal relationship (e.g. the event did not occur within a reasonable time after administration of the trial intervention). There is another reasonable explanation for the event (e.g. the patient's clinical condition, other concomitant treatment).	SAE or AE
**Possible**	There is some evidence to suggest a causal relationship (e.g. because the event occurs within a reasonable time after the trial intervention). However, the influence of other factors may have contributed to the event (e.g. the patient's clinical condition, other concomitant treatments).	SAR or AR
**Probable**	There is evidence to suggest a causal relationship and the influence of other factors is unlikely.	SAR or AR
**Definitely**	There is clear evidence to suggest a causal relationship and other possible contributing factors can be ruled out.	SAR or AR

(c) Expectedness

The expectedness of the SAE will also be accessed by the local delegated investigator an Independent Medical Monitor for the trial. The definition of an unexpected adverse reaction (UAR) is given in Table 1. If an SAE is assessed as being unexpected it becomes a SUSAR and must be reported by the sponsor to the Research Ethics Committee immediately.

(d) Recording and Reporting of Adverse Events/Adverse Reactions for this Trial

Table 3 Summary of Recording/Reporting:

Type of Event	Action Required
Adverse Event	None
Serious Adverse Event	Report within 24 hours to safety@bsuh.nhs.uk
Adverse Reaction	None
Serious Adverse Reaction	Report within 24 hours to safety@bsuh.nhs.uk

Adverse reactions are not required to be recorded or reported for this study. SARs should be recorded and reported if identified at any time throughout the trial.

Notification Procedure for SAEs and SARs:

1. The SAE form must be completed by the local Investigator (as named on the signature list and delegation of responsibilities log who is responsible for the patient's care), with the causality and expectedness of the event clearly documented. In the absence of the responsible investigator the form should be completed and signed by a member of the site trial team. The responsible investigator should subsequently check the SAE form, make changes as appropriate, sign and then send to the Brighton & Sussex CTU as soon as possible. The initial report shall be followed by detailed, written reports as appropriate.

2. Send the SAE form by email to safety@bsuh.nhs.uk within one working day of the investigator's knowledge of the event. The SAE will then have the causality and expectedness assessed by an Independent Medical Monitor.

3. Follow-up: Patients must be followed-up until clinical recovery is complete, or until the event has stabilised. Follow-up should continue after completion of protocol treatment if necessary. The patient must be identified by trial number, date of birth and initials only. The patient's name should not be used on any correspondence.

4. The sponsor will notify the research ethics committee of SUSARs as per the conditions of the favourable opinion.

Please refer to the Serious Adverse Event Reporting protocol and flowchart for further detailed information.

11 Discussion of practical and operational issues

Participants receiving the MBCT-SH intervention will be receiving an intervention that has less evidence for effectiveness than those randomised to the CBT-SH arm (hence the need for this study). There is growing evidence that MBCT self-help is effective for depression symptoms[19] so the MBCT-SH intervention is not untested. It includes elements of CBT and CBT self-help has a strong evidence for effectiveness. Participants will understand that they are free to withdraw from the MBCT-SH intervention at any time and without giving a reason. At this point they will be free to access usual care in the IAPT service.

Risk of breach to confidentiality. Unique participant identifiers will be allocated to each participant and used to record their study data on the database. These codes will be non-identifiable, however, the Principal Investigator at each site will hold a Subject Identification Log that will link the unique subject identifiers to the patients details. This document will be kept securely on NHS premises in a locked filing cabinet or on a secure NHS computer with restricted access.

12 Project timetable

Assessment	Eligibility Screen	Baseline (T0)	Intervention period	Within 16 weeks of randomisation (T1)	6 Months post intervention (T2)
Baseline Screening	x				
Informed Consent		x			
Demographics		x			
Clinical Interview Schedule	x				
Treatment Preference Questionnaire		x			
Intervention Expectation Form		x			
PHQ-9	x	x	At each PWP call	x	x
GAD7		x	At each PWP call	x	x
Wellbeing (SWEMWS)		x		x	x
Functioning (WSAS)		x	At each PWP call	x	x
Mindfulness (FFMQ-15)		x		x	x
Service Use (ADSUS)		x		x	s
Health Related Quality of Life		x		x	x

(EQ-5D-5L)					
Mindfulness-Based Cognitive Therapy Self Help intervention			x	x	
Cognitive Behavioural Therapy Self Help intervention			x	x	
Completion of Intervention Usage Diary			Weekly during intervention (T0-T1)		
PWP Rating Scale				x	
Engagement Questionnaire End of Treatment				x	
Engagement Questionnaire Follow Up					x
Lasting Effects Questionnaire					x
Hypothesis Guess Question				x	
Qualitative Change Interview					x*

*Thirty two participants only

13 Projected outputs and Dissemination

Findings will be written up for submission for open-access publication in high-impact academic journals. Four peer-reviewed papers are planned:

(1) The trial protocol will be submitted for publication in *Trials* before recruitment.

(2) A paper reporting on main findings in relation to the primary and secondary hypotheses will be submitted. This paper will be submitted to the high-impact journal (e.g. *Psychological Medicine, British Journal of Psychiatry, British Medical Journal*).

(3) The aim of a third paper will inform clinicians and researchers about the novel MBCT-SH intervention, including full details of the intervention itself, its acceptability (i.e. rates of participant engagement) and PWP training package and outcomes (e.g. fidelity ratings). Dependant on outcomes, this paper may advocate rolling the MBCT-SH intervention out widely in IAPT (if the primary hypothesis is supported) or may suggest that roll out is inadvisable (if hypotheses are not supported). This paper will be submitted to the widely-read and highly-respected UK journal *Behaviour Research and Therapy*.

(4) A paper reporting findings from the qualitative data on barriers and facilitators to engagement in Step 2 interventions for depression will be written for submission to the journal *Behaviour Research and Therapy*. This paper will include

recommendations for enhancing engagement and is well-suited to this journal that has a wide readership by clinicians and researchers.

Findings will be disseminated to participants and service user organisations. LEAP members will participate in dissemination including use of social media to disseminate findings, producing leaflets for wide distribute and submitting a summary of findings to the non-academic journal *Mental Health Today*. This will enable the findings to be widely shared within the mental health community of interest.

Findings will be presented at service user events and at local, national and international conferences including at the annual conference of the *British Association of Behavioural and Cognitive Psychotherapies*.

14 Plans for Translation

14.1 *Expected Output of Research/Impact*

If hypotheses are supported findings from this proposed study have the potential for patient benefit by improving outcomes for those referred to IAPT experiencing mild to moderate depression.

We have commitment from the five IAPT sites recruiting to the study to implement the MBCT-SH intervention if hypotheses are supported. Furthermore, if hypotheses are supported the research team will work with the national IAPT team to explore feasible ways of implementing MBCT-SH as a core Step 2 intervention for mild to moderate depression across all IAPT services. This will include developing recommendations on practical strategies for implementation (e.g. rolling out the PWP training used in the proposed study). Findings from the qualitative evaluation of barriers and facilitators to engagement will translate into recommendations for maximising client engagement in the approach. Dr Strauss is currently involved in a similar initiative with the national IAPT team and Professors Parry and Barkham also have considerable experience of working with the national IAPT team on other projects and so we are well placed to translating findings into practice if relevant.

If hypotheses are not supported and effects are in favour of CBT-SH in comparison to MBCT-SH the research team would disseminate findings as above and would feedback findings to local and national IAPT teams and to the wider mindfulness research community.

15 Gantt Chart

LIGHTMind 2 Gantt Chart

Funding month →	Mar 18	Apr 18	May 18	Jun 18	Jul 18	Aug 18	Sep 18	Oct 18	Nov 18	Dec 18	Jan 19	Feb 19	Mar 19	Apr 19	May 19	Jun 19	Jul 19	Aug 19	Sep 19	Oct 19	Nov 19	Dec 19	Jan 20	Feb 20	Mar 20	Apr 20	May 20	Jun 20	Jul 20
RAs in post with CRC support (x4)	■	■	■	■	■	■	■	■	■	■																			
Recruitment	■	■	■	■	■	■	■	■	■	■	■	■	■	■	■	■	■												
Intervention period	■	■	■	■	■	■	■	■	■	■	■	■	■	■	■	■	■	■	■	■	■								
RA (follow-ups) in post											■	■	■	■	■	■													
Post-intervention assessment		■	■	■	■	■	■	■	■	■	■	■	■	■	■	■	■	■	■	■	■	■	■	■	■	■	■	■	
Qualitative interviews (x24)		■	■	■	■	■	■	■	■	■																			
6-month follow-up assessment								■	■	■	■	■	■	■	■	■	■	■	■	■	■	■	■	■	■	■	■	■	
Research team meetings	■	■	■	■	■	■	■	■	■	■	■	■	■	■	■	■	■	■	■	■	■	■	■	■	■	■	■	■	■
TSC meetings (dates tbc)				■																									
DMEC meetings (dates tbc)			■																										
LEAP meetings (dates tbc)	■																												
PPI Deliberative Workshop																						■							
Qualitative analysis			■	■	■	■	■	■	■	■	■	■	■	■	■	■	■	■	■	■	■	■	■	■					
Quantitative analysis																						■							■
Dissemination																						■							■

The timeline is derived from our pilot RCT and so we are confident that it is feasible to adopt this approach and that rates of recruitment and retention along with other milestones are achievable.

Months 1-3: *Approvals*

NHS research ethics and governance approvals process. Given this study is, by and large, a larger version of our previous pilot RCT we do not anticipate gaining these approvals will be problematic.

Months 4-11: *Recruitment and treatment allocation*

CRN-funded CRCs will recruit and consent participants. There will be one full time research assistant (RA) in each site and an additional half time RA working across sites. RAs will meet potential participants to conduct baseline assessments.

Immediately following baseline assessment, participants will be randomised to either arm using the Sealed Envelope[29] online system. Participants will start their allocated intervention within two weeks of randomisation.

Months 8-15: *Post-intervention assessments*

Post-intervention assessments (16 weeks post-baseline) will be completed online by participants.
After post-intervention assessments are completed qualitative interviews will be conducted.

Months 14-21: *Follow-up assessments*

Six-month follow-up assessments (42 weeks post-baseline) will be completed online by participants.
Health economic measures completed.

Months 9-23: *Qualitative data analysis*

Months 21-23: *Quantitative and health economic data analysis*

Months 24-28: *Dissemination*

Months 27-28: *Implementation planning*

16 Amendments

There are no amendments

17 Competing interests

All authors declare no completing interests.

18 Acknowledgements

We would like to thank the members of the LEAP group from the pilot study and from the current study who have helped to shape the study design and who have contributed to the development of recruitment materials. We would also like to thank the participants in our pilot trial without whom the current study would not have been possible.

18 References

1. McManus, S., Meltzer, H., Brugha, T. T., Bebbington, P. P. & Jenkins, R. *Adult psychiatric morbidity in England, 2007 Results of a household survey*. (NHS Information Centre for Health and Social Care, 2009).
2. Health and Social Care Information Centre (hscic). *Psychological Therapies, Annual Report on the use of IAPT services - England, 2013-14*. (2014).
3. Health and Social Care Information Centre (hscic). *Monthly Improving Access to Psychological Therapies (IAPT) Reports. Quarter 4 2014/15*. (2015).
4. Burcusa, S. L. & Iacono, W. G. Risk for recurrence in depression. *Clin. Psychol. Rev.* **27,** 959–85 (2007).
5. McCrone, P., Dhanasiri, S., Patel, A., Knapp, M. & Lawton-Smith, S. *Paying the Price: The cost of mental health care in England to 2026*. (2008).
6. Bower, P. & Gilbody, S. Stepped care in psychological therapies: access, effectiveness and efficiency. *Br. J. Psychiatry* **186,** (2005).
7. National Institue of Health and Care Excellence [NICE]. *Common Mental Health Disorders*. (2011).
8. DeRubeis, R. J., Siegle, G. J. & Hollon, S. D. Cognitive therapy versus medication for depression: treatment outcomes and neural mechanisms. *Nat. Rev. Neurosci.* **9,** 788–796 (2008).
9. Paykel, E. S. Partial remission, residual symptoms, and relapse in depression. *Dialogues Clin. Neurosci.* **10,** 431–437 (2008).
10. Cahill, J. *et al.* Outcomes of patients completing and not completing cognitive therapy for depression. *Br. J. Clin. Psychol.* **42,** 133–43 (2003).
11. Radhakrishnan, M. *et al.* Cost of improving Access to Psychological Therapies (IAPT) programme: an analysis of cost of session, treatment and recovery in selected Primary Care Trusts in the East of England region. *Behav. Res. Ther.* **51,** 37–45 (2013).
12. Waller, R. & Gilbody, S. Barriers to the uptake of computerized cognitive behavioural therapy: a systematic review of the quantitative and qualitative evidence. *Psychol. Med.* **39,** 705–12 (2009).
13. Gu, J., Strauss, C., Bond, R. & Cavanagh, K. How do Mindfulness-Based Cognitive Therapy and Mindfulness-Based Stress Reduction Improve Mental Health and Wellbeing? A Systematic Review and Meta-Analysis of Mediation Studies. *Clin. Psychol. Rev.* **37,** 1–12 (2015).
14. Nolen-Hoeksema, S., Wisco, B. E. & Lyubomirsky, S. Rethinking Rumination. *Perspect. Psychol. Sci.* **3,** 400–424 (2008).
15. Teasdale, J. D. Metacognition, mindfulness and the modification of mood disorders. *Clin. Psychol. Psychother.* **6,** 146–155 (1999).

16. National Institute of Health and Care Excellence [NICE]. *Depression: the treatment and management of depression in adults (update).* (2009).

17. Piet, J. & Hougaard, E. The effect of mindfulness-based cognitive therapy for prevention of relapse in recurrent major depressive disorder: a systematic review and meta-analysis. *Clin. Psychol. Rev.* **31,** 1032–40 (2011).

18. Strauss, C., Cavanagh, K., Oliver, A. & Pettman, D. Mindfulness-Based Interventions for People Diagnosed with a Current Episode of an Anxiety or Depressive Disorder: A Meta-Analysis of Randomised Controlled Trials. *PLoS One* **9,** e96110 (2014).

19. Cavanagh, K., Strauss, C., Forder, L. & Jones, F. Can mindfulness and acceptance be learnt by self-help?: A systematic review and meta-analysis of mindfulness and acceptance-based self-help interventions. *Clin. Psychol. Rev.* **34,** 118–129 (2014).

20. Lever-Taylor, B., Strauss, C., Cavanagh, K. & Jones, F. The effectiveness of self-help mindfulness-based cognitive therapy in a student sample: A randomised controlled trial. *Behav. Res. Ther.* **63,** 63–69 (2014).

21. Kroenke, K., Spitzer, R. L. & Williams, J. B. The PHQ-9: validity of a brief depression severity measure. *J. Gen. Intern. Med.* **16,** 606–13 (2001).

22. Williams, M. & Penman, D. *Mindfulness: A practical guide to finding peace in a frantic world [Paperback]*. (Piatkus, 2011).

23. Strauss, C. *et al.* LIGHTMind: Low intensity guided help through mindfulness. Findings from a pilot study. in *Sussex Partnership NHS Foundation Trust Annual R&D Conference* (2015).

24. Löwe, B., Unützer, J., Callahan, C. M., Perkins, A. J. & Kroenke, K. Monitoring Depression Treatment Outcomes with the Patient Health Questionnaire-9. *Med. Care* **42,** 1194–1201 (2004).

25. Jacobson, N. S. & Truax, P. Clinical significance: a statistical approach to defining meaningful change in psychotherapy research. *J. Consult. Clin. Psychol.* **59,** 12–9 (1991).

26. Elliott, R., Slatick, E. & Urman, M. in *Qualitative psychotherapy research: Methods and methodology* (ed. Frommer, J, Rennie, D. L.) 69–111 (Pabst Science, 2001).

27. Lewis, G., Pelosi, A. J., Araya, R. & Dunn, G. Measuring psychiatric disorder in the community: a standardized assessment for use by lay interviewers. *Psychol. Med.* **22,** 465 (2009).

28. Brueton, V. C. *et al.* Strategies to improve retention in randomised trials: a Cochrane systematic review and meta-analysis. *BMJ Open* **4,** e003821 (2014).

29. Sealed Envelope Ltd. Sealed Envelope: Randomisation and online databases for clinical trials. Available at: www.sealedenvelope.com. (Accessed: 23rd March 2016)

30. Teasdale, J. D., Williams, J. M. G. & Segal, Z. *The Mindful Way Workbook: An 8-Week Program to Free Yourself from Depression and Emotional Distress*. (Guildford Press, 2014).

31. Williams, C. *Overcoming Depression and Low Mood, 3rd Edition: A Five Areas Approach [Paperback]*. (CRC Press, 2012).

32. Williams, C. *et al.* Guided self-help cognitive behavioural therapy for depression in primary care: a randomised controlled trial. *PLoS One* **8,** e52735 (2013).

33. EVANS, M., KESSLER, D., LEWIS, G., PETERS, T. J. & SHARP, D. Assessing mental health in primary care research using standardized scales: can it be carried out over the telephone? *Psychol. Med.* **34,** S0033291703008055 (2004).

34. Spitzer, R. L., Kroenke, K., Williams, J. B. W. & Löwe, B. A brief measure for assessing generalized anxiety disorder: the GAD-7. *Arch. Intern. Med.* **166,** 1092–7 (2006).

35. NHS Health Scotland & University of Warwick & University of Edinburgh. *Short Warwick-Edinburgh Mental Well-Being Scale*. (2007).

36. Stewart-Brown, S. L. *et al.* The Warwick-Edinburgh Mental Well-being Scale: A Valid and reliable tool for measuring mental well-being in diverse populations and projects. *J. Epidemilogy Community Heal.* **11,** (2011).

37. MUNDT, J. C. The Work and Social Adjustment Scale: a simple measure of impairment in functioning. *Br. J. Psychiatry* **180,** 461–464 (2002).

38. Gu, J. *et al.* Examining the factor structure of the 39-item and 15-item versions of the five-facet mindfulness questionnaire before and after mindfulness-based cognitive therapy for people with recurrent depression. *Psychol. Assess.*

39. Kuyken, W. *et al.* Effectiveness and cost-effectiveness of mindfulness-based cognitive therapy compared with maintenance antidepressant treatment in the prevention of depressive relapse or recurrence (PREVENT): a randomised controlled trial. *Lancet* **386,** 63–73 (2015).

40. National Institute of Health and Care Excellence [NICE]. *Guide to the methods of technology appraisal*. (NICE, 2013).

41. Brooks, R. EuroQol: the current state of play. *Health Policy* **37,** 53–72 (1996).

42. Herdman, M. *et al.* Development and preliminary testing of the new five-level version of EQ-5D (EQ-5D-5L). *Qual. Life Res.* **20,** 1727–36 (2011).

43. Bower, P. *et al.* Influence of initial severity of depression on effectiveness of low intensity interventions : meta-analysis of individual patient data. **540,** 1–11 (2013).

44. Boggs, J. M. *et al.* Web-based intervention in mindfulness meditation for reducing residual depressive symptoms and relapse prophylaxis: a qualitative study. *J. Med. Internet Res.* **16,** e87 (2014).

45. Schulz, K. F., Altman, D. G. & Moher, D. CONSORT 2010 statement: updated guidelines for reporting parallel group randomised trials. *BMJ* **340,** c332 (2010).

46. Drummond, M. F., Sculpher, M. J., Claxton, K., Stoddart, G. L. & Torrance, G. W. *Methods for the economic evaluation of health care programmes*. (Oxford University Press, 2015).

47. Efron, B. & Tibshirani, R. J. *An introduction to the bootstrap*. (Chapman Hall, 1993).

48. Thompson, S. G. & Barber, J. A. How should cost data in pragmatic randomised trials be analysed? *Br. Med. J.* **320,** 1197–1200 (2000).

49. Fenwick, E., Claxton, K. & Sculpher, M. Representing uncertainty: The role of cost-effectiveness acceptability curves. *Health Econ.* **10,** 779–787 (2001).

50. Braun, V. & Clarke, V. Using thematic analysis in psychology. *Qual. Res. Psychol.* **3,** 77–101 (2006).

51. Medical Research Council. *MRC Guidelines for Good Clinical Practice in Clinical Trials*. (1998).

19 Appendices

The following measures and tools will be used and are attached. Time points are baseline (T0), 16 weeks post-randomisation (T1) and 42 weeks post-randomisation (T2).

1. **Treatment preference question**. Completed prior to consent (before T0)

<u>Participant Information</u>

2. **Demographic questionnaire.** (T0)

<u>Diagnostic Tools</u>

3. **CIS-R**. This will be conducted at baseline to ascertain diagnostic status in relation to inclusion criteria (major depression or mixed anxiety and depression) (eligibility screening assessment)

<u>Outcome/process measures</u>

4. **PHQ-9**. This is a 9-item self-report measure of depression symptom severity. This is the primary outcome measure (eligibility screening assessment, T0, T1, T2 + at each PWP support session)
5. **GAD-7**. This is a 7-item measure of generalised anxiety. (T0, T1, T2 + at each PWP support session)
6. **SWEMWS**. This is the short version of the Warwick Edinburgh Mental Wellbeing Scale and consists of 7 questions rated on a 5-point scale designed to measure wellbeing. (T0, T1, T2)
7. **WSAS**. The Work and Social Adjustment Scale (WSAS) is a 5-item measure of daily occupational and social functioning. (T0, T1, T2 + at each PWP support session)
8. **FFMQ-15**. Mindfulness will be measured using 15-item version of the Five-Facet Mindfulness Questionnaire. (T0, T1, T2)
9. **ADSUS**. A self-report version of the Adult Service Use Schedule will be used to collect data suitable for estimation of costs and taking the health and social services perspective. (T0, T1, T2)
10. **EQ-5D-5L**. The EQ-5D is a five-dimension, generic, preference-based measure of health-related quality of life covering mobility, self-care, usual activities, pain/discomfort and anxiety/depression. (T0, T1, T2)

<u>Self-help course evaluation tools</u>

11. **Intervention expectation form**. This will be used to assess expectation of benefit and treatment credibility (T0)
12. **Lasting effects questionnaire**. This will be used to ask participants about any lasting negative effects of their intervention (T2)
13. **PWP rating scale.** This will be used for participants to rate the quality/helpfulness of the support sessions between the participant and their PWP (T1)
14. **Weekly diary**. These record the extent to which participants are engaging with the self-help course each week during the self-help course (weekly between T0 and T1)

15. **Engagement questionnaire (end of treatment)**. This records the extent to which participants engaged with the self-help course during the entire course of the intervention (T1)
16. **Engagement questionnaire (follow-up)**. This records the extent to which participants continued to engage with the self-help course following the end of the intervention (T1)
17. **Change Interview**. This semi-structured interview will be used to assess participants' experiences of change during their allocated intervention (T2 following completion of quantitative measures)

Additional measures and tools

18. **PWP Session Record**. Number and duration of PWP sessions attended and action taken following final session (T0 to T1)
19. **Hypothesis Guess Question**. Participants will be asked to guess the purpose of the study (T2)
20. **End of Questionnaire Information**. Information on who to contact in case of distress following completion of questionnaires (T0, T1 and T2)
