# Supplementary material for: Clinical Effectiveness and Cost-Effectiveness of Supported Mindfulness-Based Cognitive Therapy Self-help Compared With Supported Cognitive Behavioral Therapy Self-help for Adults Experiencing Depression: The Low-Intensity Guided Help Through Mindfulness (LIGHTMind) Randomized Clinical Trial
Source: JAMA Psychiatry. 2023 Mar 22;80(5):415–24. doi: 10.1001/jamapsychiatry.2023.0222 (PMC10034662; doi:10.1001/jamapsychiatry.2023.0222)
Supplement: Supplement 2. — Statistical Analysis Plan [file jamapsychiatry-e230222-s002.pdf]

LightMIND 2

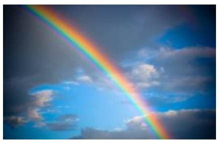

Low-Intensity Guided Help Through MINDfulness 2

A randomised controlled trial comparing supported Mindfulness-Based Cognitive Therapy self-help to supported Cognitive Behaviour Therapy self-help for adults experiencing depression

Statistical Analysis Plan

SAP Version:1.0

Status: Approved

Date: 02/08/2019

ISRCTN3495752

Approval signatures

Role	Name	Signature	Date
**Trial Statistician**	**Anna-Marie Jones**	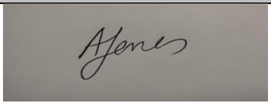	**18th Dec 2020**
**Principle Investigator**	**Clara Strauss**	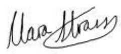	18 Dec 2020
**Trial Manager**	**Amy Arbon**	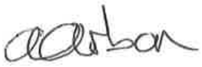	21 Dec 2020

1. Introduction

This document details the statistical analysis plan for the LIGHTMind2 trial.

2. Background and Rationale

Depression has serious personal, family and economic consequences. It is estimated that depression will cost £12.15 billion to the economy each year in England by 2026. Improving Access to Psychological Therapies (IAPT) is the NHS talking therapies service in England for adults experiencing anxiety or depression. Approximately 1 million people are referred to IAPT every year, over half experiencing depression. Where symptoms of depression are mild/moderate people are typically offered Cognitive Behaviour Therapy (CBT) self-help supported by a psychological wellbeing practitioner (PWP).

The problem is that over half of people (58%) who complete treatment for depression in IAPT remain depressed despite receiving the NICE-recommended treatment. Furthermore, less than half (40%) of IAPT clients complete treatment. This study seeks to investigate an alternative to CBT self-help. We suggest that mindfulness-based self-help – which differs from CBT in focus, approach and practice – would be more effective with lower dropout. We conducted a study with 40 people comparing these two forms of self-help (CBT and Mindfulness). We found people using mindfulness-based self-help showed greater improvement in depression and twice as many people completed mindfulness-based self-help.

In this proposed study, 410 people experiencing mild-moderate depression will be randomly allocated to using a mindfulness-based self-help workbook or to a CBT-based self-help workbook. Each person will be asked to complete their workbook within 16 weeks and will have six PWP support sessions during this time. The primary outcome we will measure is depression symptom severity. Treatment completion will also be measured because evidence shows completing treatment is associated with better outcomes. Assessments will be made at the start of the study and then again after 16 weeks and then after a further 6 months and 40 participants will also be interviewed about their experiences after the 6 month follow up assessment period. Findings will provide evidence for policy makers and will help to inform decision making about mindfulness-based self-help in the NHS

1. The study objectives

Primary Objective: to conduct a definitive RCT to compare MBCT-SH with CBT-SH for people experiencing mild to moderate depression.

Secondary Objectives:

- To evaluate the cost-effectiveness
- To identify facilitators and barriers to treatment completion for each intervention
- To assess the PWP's fidelity to the intervention protocol

Primary Hypothesis:

- supported MBCT-SH, in comparison to supported CBT-SH, will lead to greater reductions in depressive symptom severity (PHQ-9[12]) from baseline to post-intervention.

Secondary hypotheses are:

- MBCT-SH in comparison to CBT-SH will lead to greater reduction in depressive symptom severity from baseline to six-months follow-up.
- A greater proportion of MBCT-SH participants will be in the non-clinical range for depressive symptoms than CBT-SH participants at post-intervention (i.e. remission) and six-months follow-up (i.e. recovery).
- MBCT-SH in comparison to CBT-SH will lead to greater improvements in mindfulness, generalised anxiety, work and social adjustment and wellbeing from baseline to post-intervention and from baseline to six-months follow-up.
- Treatment completion rates will be higher for MBCT-SH than CBT-SH.
- Depressive symptom severity outcomes will be mediated by treatment completion.
- MBCT-SH will be cost-effective in comparison to CBT-SH at follow-up.

2. Study design

This is a parallel groups, superiority pragmatic Randomised Controlled Trial (RCT) with 1:1 allocation to MBCT-SH or CBT-SH with blinded assessments at all time points. Participants will be blind to the hypothesised direction of effects.

Four hundred and ten people meeting eligibility criteria for major depressive disorder or mixed anxiety and depression will be randomly allocated to receive MBCT-SH or CBT-SH, along with six sessions of support from a psychological wellbeing practitioner. Participants will complete measures at baseline (T0), 16 weeks post-randomisation (post-intervention; T1) and 42 weeks post randomisation (6-months follow-up; T2).

In addition, 40 participants will be interviewed about their experiences.

Participants will be recruited from 10 IAPT services:

(1) Brighton and Hove Wellbeing Service

(2) East Riding Emotional Wellbeing Service
(3) Health in Mind in East Sussex
(4) Health in Mind in North-East Essex
(5) italk in Hampshire
(6) Lewisham IAPT service
(7) South West Yorkshire Partnership NHS Foundation Trust
(8) Talking Change in Portsmouth
(9) Talking Therapies Southwark
(10) Time to Talk in West Sussex

3. A description of the study population, and the analysis populations, detailing inclusion and exclusion rules

The study population will be those described as having mild to moderate depression with the following inclusion and exclusion criteria:

Inclusion criteria are that participants will:

(1) be aged 18 years or over;
(2) meet diagnostic criteria on the revised Clinical Interview Schedule (CIS-R)[27] for a primary diagnosis of a depressive episode, mixed anxiety and depression, or non-specified mild neurotic disorder at their eligibility screening assessment;
(3) score 10 or more on the PHQ-9[21] at their eligibility screening assessment (the cut-off for a major depressive episode); and
(4) have sufficient literacy skills to read and understand the self-help materials

Exclusion criteria are that if people:

(1) have severe symptoms of depression at their eligibility screening assessment (a score 20 or more on the PHQ-9);
(2) score of 4 on the CIS-R suicidality scale
(3) express a strong preference (5/5) for one intervention over the other on the Treatment Preference Question such that if randomised to the non-preferred intervention they would be likely to drop out of the intervention.
(4) Interventions
(5) The MBCT-SH workbook 'The Mindful Way Workbook'[30],
(6) CBT-SH workbook 'Overcoming Low Mood and Depression'[31]

4. The primary and secondary outcome measures (and measurement time point)

Primary outcome measure

The primary outcome measure is *Depression symptom severity which is measure using the Patient Health Questionnaire (PHQ-9)*[21]. The PHQ-9 is a 9-item self-report measure of depression symptom severity use. Items are rated on a four-point scale. Scores under 10 are considered sub-clinical, 10-14 mild, 15-19 moderate and 20+ severe (eligibility screening assessment, T0, T1, T2, plus at each PWP support session).

Secondary outcome measures

- *Generalised anxiety (GAD-7)*[34]. This is a 7-item measure of generalised anxiety used in IAPT. Items are rated on a 4-point scale and the measure has excellent psychometric properties[34] (T0, T1, T2, plus at each PWP support session).
- *Wellbeing (SWEMWS)*[35]. The short version of the Warwick Edinburgh Mental Wellbeing Scale consists of 7 questions rated on a 5-point scale designed to measure wellbeing. The scale has good psychometric properties and is used widely[36]. This measure was added following advice from the PPI consultation panel (T0, T1, T2).
- *Functioning (WSAS)*[37]. The Work and Social Adjustment Scale (WSAS) is a 5-item measure of daily occupational and social functioning that is used routinely in IAPT (T0, T1, T2, plus at each PWP support session).
- *Mindfulness (FFMQ-15)*[38]. Mindfulness will be measured using 15-item version of the Five-Facet Mindfulness Questionnaire. This has excellent psychometric properties and is sensitive to change following MBCT[38] (T0, T1, T2).

Intervention Evaluation Measures and Tools

- *Intervention expectation form*. This will be used to assess expectation of benefit and treatment credibility (T0)
- *Lasting effects questionnaire*. This will be used to ask participants about any lasting negative effects of their allocated intervention (T2)
- *PWP rating scale*. This will be used for participants to rate the quality/helpfulness of the support sessions between the participant and their PWP (T1)
- *Weekly diaries*. These record the extent to which participants are engaging with the self-help course each week during the self-help course (weekly between T0 and T1)
- *Engagement questionnaire (end of treatment)*. These records the extent to which participants engaged with the self-help course during the entire course of the intervention (T1).
- *Engagement questionnaire (follow-up)*. This records the extent to which participants continued to engage with the self-help course following the end of the intervention (T2)
- *Session attendance*. Number of PWP sessions attended (0-6) and duration of each support session.
- *Treatment completion*. This is defined as attending at least three PWP sessions.

Demographics

- Gender
- Whether identify as Transgender
- Marital status
- Number of children <18yrs
- Number of children >=18yrs
- Highest Education level
- Occupational status
- Ethnicity
- Sexual Orientation
- Whether English is the first language
- Whether had CBT or mindfulness-based therapy in the past

5. Interventions

*MBCT Self-h*elp

The MBCT-SH workbook 'The Mindful Way Workbook'[30], written for clinical populations, presents MBCT as a self-help package. MBCT-SH participants will be given the workbook and will be asked to guide themselves through the self-help course within a 16 week time period (the time period determined in our pilot). As is routine at Step 2, participants will be offered six PWP sessions to answer questions and provide encouragement.

CBT Self-help

The CBT-SH workbook 'Overcoming Low Mood and Depression'[31] has evidence demonstrating its effectiveness in reducing depression symptom severity[32]. Participants allocated to CBT-SH are given a copy of their workbook and are encouraged to guide themselves through within 16 weeks alongside six PWP sessions to answer questions and provide encouragement.

6. The sample size justification

The sample size was based on detecting a between-group effect size of 0.36 based on the difference between the reported between-group effect of CBT-SH (0.42)[43] and the reported between-group effect of MBCT-SH (0.78)[44]. Recruiting 205 patients into each arm would provide 90% power to detect a between-group difference of 0.36 with a 5% alpha and a two-sided t-test whilst allowing for 20% attrition at post-intervention (as found in the pilot RCT); therefore a total sample size of 410 will be required.

In our pilot RCT of the same design we recruited 40 participants in one of the five sites in six months with a 40% FTE RA. This equates to recruiting 466 participants in the eight month recruitment window of the proposed study. This means that it is feasible to recruit to the 410 participant target whilst allowing for an up to 10% shortfall in expected recruitment rates.

7. Blinding

Post and follow-up questionnaires are completed by participants online on their own with a standardised email sent with the link to the online questionnaire. This means that post & follow-up assessments are blind as a researcher isn't present when they are completed and the email used send instructions to complete the questionnaires is the same for all participants. Should a participant request support with completing post questionnaire from an RA by phone, the RA would be blinded to the participant's allocation (in practice this is likely to be a rare request).

8. Allocation and Concealment

Randomisation is stratified by centre and PHQ-9 score (mild or moderate) using random block length.

Eligible participants will be randomly allocated using the Sealed Envelope[29] online service. The team statistician will use Sealed Envelope to set up and test the randomisation procedure incorporating stratification by site and PHQ-9 severity category (mild or moderate) using random block length and 1:1 allocation. The statistician will not have any further involvement in the randomisation process. The RA will randomise participants by completing the online form with participant's details. This will immediately show whether the participant is assigned to the MBCT-SH or CBT-SH arm and participants will be given their self-help workbook. An allocation email is automatically sent to the trial manger. Participants will not be told the hypotheses in relation to the arm they have been randomised to.

9. Data Collection and Data Management

Data is collected via Qualtrics™ the online survey facility. The Research Assistants set up separate surveys for the each of the 3 time points. At the time of data collection the RA sends a link to the participant asking them to complete it. Following non-response, text/email reminders are sent up to a maximum of 3 occasions.

Data is downloaded from Qualtrics into excel and then imported into STATA for analysis by the statistician.

Paper versions of the questionnaire are entered into Qualtrics by the RA and then a data quality check is carried out at N=5, N=10 and N=20. The process is led by Statistician and carried out by an RA not involved in data collection for that site.

At the point of coding the following missing data values will be used.
666 = Dropped out
888 = Not applicable
999 = system missing/missing from questionnaire
1/1/999 = missing date

A log of data quality checks will be recorded detailing the process and who was involved. Any data updated as a result of the data quality check will also be logged in a data monitoring log.

At the end of the data collection process the dataset will be cleaned then a final dataset will be presented for data analysis. At this time no further changes can be made to the data and the data will be locked.

Participant flow through the study will be reported in line with the Consolidated Standards of Reporting Trials (CONSORT) 2010 statement[45].

10. The statistical methodology:

Quantitative analyses will be based on:

intention-to-treat approach, where participants are analysed as per their randomisation allocation regardless of treatment received, and in addition

per protocol analysis will be conducted for those participants receiving an adequate dose of their allocated intervention (defined as completing at least 50% of their allocated intervention).

A descriptive summary of all measures will be provided by group (MBCT-SH & CBT-SH) and time point (T0, T1, T2) as appropriate.

Comparisons between independent groups for intervention evaluations measures will be carried out using independent t-tests and Chi-square testing for continuous and categorical data, respectively.

Unstandardised effect sizes for the primary outcome and secondary outcomes will be estimated using linear mixed models with treatment group (MBCT-SH vs CBT-SH), time (16 and 42 weeks) and a treatment group by time interaction entered as fixed factors; site, baseline PHQ-9 and baseline value of the outcome will be entered as covariates. Individual participants will be included in the analysis as random effects. Contrasts will be used as appropriate to estimate effects at different time points. A non-significant group by time interaction will imply common treatment effects at each time point.

Standardised (Cohen's *d*) effect sizes for each outcome will be calculated by dividing the between-group unstandardized effect by the baseline pooled standard deviation.

95% confidence intervals will be calculated for all unstandardised estimates.

Group differences in dichotomous outcomes at the different time points will be analysed in a similar way but using multilevel logistic regression models and baseline PHQ-9 scores.

Baseline balance will be presented in the descriptive table broken down by study arm. No adjustment will be made for differences between co-variates at baseline.

Outliers will be removed if and only if they look erroneous. Scores at the extreme will not be removed if they are deemed to be true.

The suitability of the assumption of approximate normality will be explored by plotting the residuals from this model. If normality is violated then transformations and non-parametric testing will be employed.

A sensitivity analysis will be carried out by carrying out the final analysis on the primary outcome, with and without any individual cases what were involved in violations of the protocol.

Additional exploratory analysis

In the event that there is no evidence to support the primary hypothesis of MBCT-SH superiority over CBT-SH on our primary outcome, an additional analysis will be carried out to explore non-inferiority of MBCT-SH compared to CBT-SH. The analysis will be based on detecting a between-group non-inferiority margin of 2 with a one-sided $\alpha = 0.025$. To operationalise this, a two-sided 95% confidence interval will be created around the effect size (MBCT-SH – CBT-SH) and we will conclude non-inferiority if the upper limit of the CI is wholly below 2 for both the PP and ITT analyses. The non-inferiority limit was set through consultation with service users and clinicians and looking at the literature (Cobra & PRaCTICED).

Study	NI Margin	Reference

Cobra study	1.9	https://www.thelancet.com/journals/lancet/article/PIIS0140-6736(16)31140-0/fulltext
PRaCTICED	2	https://trialsjournal.biomedcentral.com/track/pdf/10.1186/s13063-017-1834-6

Missing data

We aim to minimise missing data at the point of collection. The Qualtrics software used to collect data will automatically flag any unanswered questions, giving participants to chance to answer these. If a participant would prefer not to answer a question they can leave it unanswered for a second time and the software will proceed onto the next page.

At the point of analysis: Data will be summarised to look at patterns of missingness. Missing data will replaced using multiple imputation as appropriate.

Missing data will be assessed and if more than 5% of data is missing multiple imputation will be carried out followed by a sensitivity analysis. The sensitivity analysis will compare the results for a complete case analysis to the imputed data analysis. Multiple imputation will be carried out under the assumption the data is missing at random (MAR).

Planned interim analysis and stopping rules

No interim analysis has been planned. The trial will be stopped if deemed necessary by the Data Monitoring and Ethics Committee.

Multiple testing

There shall be no multiple testing.

11. A description of serious adverse event/adverse event recording and reporting

As per the Final Study Protocol.

12. Documented changes to the SAP after final sign off

Change to SAP	Reason	Signature	Date

13. Appendices:

13.1. [Add scoring]
13.2. Main shell tables for interim reports e.g. to TSC/DMC and for final reports (monographs, main study papers)
13.3. Data collection instruments (copies should be filed in the SMF)
13.4. A data dictionary
13.5. A full set of references

Table 1 Descriptive summary for demographic variables

	Treatment<br><br>N		Control<br><br>N		Total<br><br>N	
Gender:						
Male	n	%	n	%	n	%
Female	n	%	n	%	n	%
Identify as another term	n	%	n	%	n	%
Whether Transgender:						
Yes	n	%	n	%	n	%
No	n	%	n	%	n	%
Age (years) Median /range						
Ethnicity :						

White British	n	%	n	%	n	%
BME	n	%	n	%	n	%
Sexual Orientation						
Heterosexual	n	%	n	%	n	%
Gay	n	%	n	%	n	%
Bisexual	n	%	n	%	n	%
Lesbian	n	%	n	%	n	%
Identify as another term	n	%	n	%	n	%
Marital Status:						
Single	n	%	n	%	n	%
Married/Civil Partnership	n	%	n	%	n	%
Living with Partner/Cohabiting/Long Term relationship	n	%	n	%	n	%
Divorced/Separated	n	%	n	%	n	%
Widowed	n	%	n	%	n	%

Employment Status:						
Unemployed	n	%	n	%	n	%
Employed	n	%	n	%	n	%
Home maker/Carer	n	%	n	%	n	%
Student	n	%	n	%	n	%
Retired	n	%	n	%	n	%
Other	n	%	n	%	n	%
Education Level:						
None	n	%	n	%	n	%
GCSE or equivalent	n	%	n	%	n	%
A' Level or equivalent	n	%	n	%	n	%
Undergraduate or equivalent	n	%	n	%	n	%
Post graduate or equivalent	n	%	n	%	n	%

Symptom Severity: CIS-R						
Mild	n	%	n	%	n	%
Moderate	n	%	n	%	n	%
Severe	n	%	n	%	n	%
Therapy Expectation:						
Expectation of benefit	Mean (sd)		Mean (sd)		Mean (sd)	
Credibility	Mean (sd)		Mean (sd)		Mean (sd)	

Table 2 Descriptive Summary of Baseline outcomes MBCT-SH/CBT-SH/Total

	N	*Mean*	*Std. Deviation*	*Median*	*Min-Max*	*IQR*
PHQ-9						
SWEMWS						
GAD-7						

FFMQ-15						
WSAS						

Table 3a Descriptive summary of adherence (treatment completion) rates

Treatment	Adherence<br>Yes = 1	Adherence<br>No = 0	*Total*		*Observed proportion adhering*	*Anticipated proportion of successes*	*Odds Ratio*
MBCT	a	b	m		a/m	p1	
CBT	c	d	n		b/n	p2	
TOTAL	r	s	N				

Table 3b Descriptive Session Attendance

	MBCT-SH		CBT-SH	
Number of sessions attended	Counts	Proportion	Counts	Proportion
1	n	%	n	%
2	n	%	n	%
3	n	%	n	%

4	n	%	n	%
	mean		mean	

Table 3c Summary of PWP ratings

Sub Score:	MBCT-SH			CBT-SH		
	N	Mean (SD)	range	N	Mean (SD)	range
Relationship	xx	xx	xx	xx	xx	xx
Goal	xx	xx	xx	xx	xx	xx
Approach	xx	xx	xx	xx	xx	xx
Overall Score	xx	xx	xx	xx	xx	xx

Table 3c Summary Engagement – TBC by Study Arm

Sub Score:			

Table 4 Counts of Valid Cases and Missing Values at each time point for the key variables, by group and overall: N

Variable	Baseline		Post Group		Follow-up	
	Valid cases	Missing cases (%)	Valid cases	Missing cases (%)	Valid cases	Missing Cases (%)
PHQ-9						
SWEMWS						
FFMQ-SF						
GAD-7						
WSAS						

Table 5 & 6 Descriptive summary of post-changes: differences; comparison of change scores in the treatment and control group from Baseline to post scores/Baseline to follow-up (ITT/PP)

Outcome	What change means improve-ment +/-	N	Treatment		Control		Pooled SD	Difference (95% CI)	Effect size (d)
			Mean change	SD	Mean change	SD			
PHQ-9	-								

SWEMWS	+								
FFMQ-SF	+								
GAD-7	-								
WSAS	-								

Table 7 & 8 Effect size results from the Linear Model unadjusted and adjusted for missingness (ITT/PP)

Outcome	Unadjusted / adjusted				
	Estimate	SE	95% CI	P	Cohen's d
PHQ-9					
SWEMWS					
FFMQ-SF					
GAD-7					
WSAS					

Table 9 Summary of adverse events

Adverse event	Treatment	Control
Event 1	N	
Event2…	N	
Total	N adverse events	N_adverse events
