# Supplementary material for: Clinical Effectiveness and Cost-Effectiveness of Supported Mindfulness-Based Cognitive Therapy Self-help Compared With Supported Cognitive Behavioral Therapy Self-help for Adults Experiencing Depression: The Low-Intensity Guided Help Through Mindfulness (LIGHTMind) Randomized Clinical Trial
Source: JAMA Psychiatry. 2023 Mar 22;80(5):415–24. doi: 10.1001/jamapsychiatry.2023.0222 (PMC10034662; doi:10.1001/jamapsychiatry.2023.0222)
Supplement: Supplement 3. — eMethods. eResults. eDiscussion. eFigure. PHQ-9 Scores by Trial Arm and Timepoint eTable 1. Descriptive Summary for Demographic Variables Comparing PHQ-9 T1 Data Completers With T1 Data Noncompleters at 16 Weeks eTable 2. Descriptive Summary of Baseline Clinical Measures Comparing PHQ-9 T1 Data Completers With T1 Data Noncompleters at 16 Weeks eTable 3. Descriptive Summary of Clinical Outcomes, Effect Estimates, and Effect Sizes for Observed-Cases Per-Protocol Analysis eTable 4. Between-Group Effect Estimates and Effect Sizes for Modified Intention-to-Treat Analysis With Observed Cases and Multiple Imputation eTable 5. Between-Group Effect Estimates and Effect Sizes for Per-Protocol Analysis With Observed Cases and Multiple Imputation eTable 6. Between-Group PHQ-9 Effect Estimates and Effect Sizes by Sex, Ethnicity, and Baseline Medication Use Using Observed Cases and Multiple Imputation eTable 7. Proportion of Participants in the Nonclinical Range (PHQ-9 score of 0-9) for Depressive Symptoms at 16 and 42 Weeks eTable 8. PWP Session Adherence eTable 9. Lasting Negative Effects by Study Arm eAppendix 1. Further Details of Health Economic Evaluation eAppendix 2. The Role of Experts by Experience eReferences. [file jamapsychiatry-e230222-s003.pdf]

Supplemental Online Content

Strauss C, Bibby-Jones AM, Jones F, et al. Clinical effectiveness and cost-effectiveness of supported mindfulness-based cognitive therapy self-help compared with supported cognitive behavioral therapy self-help for adults experiencing depression: the Low-Intensity Guided Help Through Mindfulness (LIGHTMind) randomized clinical trial. *JAMA Psychiatry*. Published online March 22, 2023. doi:10.1001/jamapsychiatry.2023.0222

eMethods.
eResults.
eDiscussion.
eFigure. PHQ-9 Scores by Trial Arm and Timepoint
eTable 1. Descriptive Summary for Demographic Variables Comparing PHQ-9 T1 Data Completers With T1 Data Noncompleters at 16 Weeks
eTable 2. Descriptive Summary of Baseline Clinical Measures Comparing PHQ-9 T1 Data Completers With T1 Data Noncompleters at 16 Weeks
eTable 3. Descriptive Summary of Clinical Outcomes, Effect Estimates, and Effect Sizes for Observed-Cases Per-Protocol Analysis
eTable 4. Between-Group Effect Estimates and Effect Sizes for Modified Intention-to-Treat Analysis With Observed Cases and Multiple Imputation
eTable 5. Between-Group Effect Estimates and Effect Sizes for Per-Protocol Analysis With Observed Cases and Multiple Imputation
eTable 6. Between-Group PHQ-9 Effect Estimates and Effect Sizes by Sex, Ethnicity, and Baseline Medication Use Using Observed Cases and Multiple Imputation
eTable 7. Proportion of Participants in the Nonclinical Range (PHQ-9 score of 0-9) for Depressive Symptoms at 16 and 42 Weeks
eTable 8. PWP Session Adherence
eTable 9. Lasting Negative Effects by Study Arm
eAppendix 1. Further Details of Health Economic Evaluation
eAppendix 2. The Role of Experts by Experience
eReferences.

This supplementary material has been provided by the authors to give readers additional information about their work.

© 2023 Strauss C et al. *JAMA Psychiatry*.

eMethods.

Secondary Hypotheses

Pre-registered secondary hypotheses not specified in the main manuscript were that:

- Supported MBCT-SH, in comparison to CBT-SH, will lead to greater reduction in depressive symptom severity from baseline to 42-weeks post-randomisation (follow-up);
- A greater proportion of MBCT-SH participants will be in the non-clinical range for depressive symptoms than CBT-SH participants at post-intervention and follow-up;
- MBCT-SH, in comparison to CBT-SH, will lead to greater improvements in mindfulness, generalized anxiety, work and social adjustment and wellbeing from baseline to post-intervention and from baseline to follow-up;
- Treatment completion rates will be higher for MBCT-SH than CBT-SH;
- Depressive symptom severity outcomes will be mediated by treatment completion

Secondary Outcome Measures

Secondary outcome measures not detailed in the main manuscript were:

- Generalized anxiety (Generalized Anxiety Disorder scale [GAD-7])[1].
- Wellbeing (short version of the Warwick Edinburgh Mental Wellbeing Scale [SWEMWS])[2].
- Functioning (Work and Social Adjustment Scale [WSAS])[3].
- Mindfulness (15-item version of the Five Facet Questionnaire [FFMQ-15] minus the Observe subscale)[4].

The above measures were completed at T0, T1 and T2.

Treatment completion was defined as attending at least 50% of the 6 Psychological Wellbeing Practitioner (PWP) support sessions (i.e., at least 3 sessions).

Additional Procedural Details

IAPT Service Selection

Improving Access to Psychological Therapies (IAPT) is an England-wide initiative established in 2008 and funded through the National Health Service. These services provide evidence-based psychological therapies, free at the point of access, to adults experiencing common mental health problems. Psychological therapy is provided at two levels of intensity. Step Two interventions involve brief interventions and guided use of CBT self-help resources supported by Psychological Wellbeing Practitioners. Step Three interventions involve formal CBT and other evidence-interventions delivered by trained and accredited CBT/other psychological therapists. A stepped care approach is

© 2023 Strauss C et al. *JAMA Psychiatry*.

employed whereby people are offered the least intrusive intervention in the first instance to support their recovery. Following Step Two, people can be 'stepped up' to Step Three where necessary. This stepped approach allows IAPT services to have a broad reach. The current study was within Step Two of IAPT services.

Ten IAPT services took part in the study. These services were selected to ensure that the study had good geographical and demographic coverage and included a range of urban and rural communities. The services were in the English counties of Sussex, Hampshire, Inner London, Essex and Yorkshire. Sussex, Hampshire and Yorkshire services included people living in urban (large towns and cities), small town and rural communities. Essex services included people living in small towns and rural communities. The Inner London and Yorkshire services served communities that were particularly ethnically diverse.

<u>Additional Details on Intervention Adherence</u>

To support adherence, PWPs were trained in and provided with detailed session-by-session protocols for both arms of the study. As the emphasis in the study was on supporting people to guide themselves through their allocated workbook (MBCT-SH or CBT-SH), the detailed session protocols focused on exploring experiences of using the workbook since the last session and on addressing questions and challenges arising from the workbook. Each PWP session had the following format which was outlined in detail in the sessional protocol (please contact the corresponding author for further details of the protocols).

1. Welcome and reminder of session outline
2. Check understanding of the workbook chapter(s) covered since the last PWP session
3. Invite questions about the workbook chapter(s) covered since the last PWP session
4. Explore learning from a recent mindfulness practice (MBCT-SH arm) or from a recent CBT exercise/task (CBT-SH arm)
5. Identify workbook chapter(s) to cover before the next PWP session
6. Identify and address any anticipated challenges with intervention engagement

Fortnightly telephone supervision covered both arms of the study. In these supervision sessions, PWPs were expected to describe their recent sessions with each participant they were currently working with in line with the session-by-session protocol. They identified any challenges and these were discussed in relation to the session protocol (i.e. how the sessional protocol could be used to address challenges).

© 2023 Strauss C et al. *JAMA Psychiatry*.

eResults.

Findings on clinical outcomes not reported in the main manuscript are described below.

For depression-related secondary hypotheses, at T1, the ITT analysis showed that significantly more MBCT-SH (74%) than CBT-SH (63%) participants scored in the non-clinical range on the PHQ-9 (aOR=1·13; 95% CI 1·02-1·24; *p*=0·014) – see eTable 7. This difference was not maintained at T2 with 78% and 76% (aOR=1·02, 95% CI 0.94-1.12; *p*=0·588) in the non-clinical range for the MBCT-SH and CBT-SH arms, respectively. The observed-cases per protocol analyses on the PHQ-9 at T1 and T2 resulted in a statistically non-significant between-group difference in favor of MBCT-SH (see eTable 3).

For other secondary outcomes not reported in the main manuscript, in the ITT analysis detailed in Table 2, there were non-significant differences between trial arms at T1 or T2 on measures of work and social adjustment or wellbeing.

Sensitivity analyses of clinical outcomes showed consistency in all but one instance, when ITT findings were compared to those from the MICE analysis: the between-group difference in generalized anxiety at 16-weeks was significant for the ITT analysis but non-significant for the MICE analysis. See eTable 4 for details.

eTable 6 shows between-group PHQ-9 effect estimates and effect sizes by sex, ethnicity and baseline medication use conducted as unplanned subgroup analyses. The findings show variability by participant characteristic and time point, but all findings were in the hypothesized direction. Of interest are the larger (but not statistically significant) effect sizes for females (compared to males at both time points), Ethnic Minorities (compared to White British at 16 weeks), and those not using medication (compared to using medication at both time points). However, across the board there are large degrees of overlap in 95% confidence intervals between the comparative groups. In addition, this clinical trial was not powered to make comparisons within subgroups, so hypothesis tests were not carried out and caution should be taken when interpreting the findings.

Descriptive summaries of demographic and baseline clinical measures were compared between the whole sample (All) and those who completed the PHQ-9 primary outcome measure at 16 weeks (T1 Study Completers) with no differences found at the p<.05 level – see eTable 1 and eTable 2. These analyses were repeated within study arm and a similar pattern of findings was observed. Seven (3%) participants in the MBCT-SH arm and 15 (7%) in the CBT-

© 2023 Strauss C et al. *JAMA Psychiatry*.

SH arm did not attend any PWP sessions. The mean (SD) number of PWP sessions was 4·5 (2·0) in MBCT-SH and 4·3 (2·2) in CBT-SH. Overall, 77·0% of MBCT-SH participants and 73·3% CBT-SH completed treatment (i.e. attended 3+ PWP sessions), which gave a non-significant aOR=1·21 (95% CI 0·77-1·90). Treatment effects on depressive symptom severity were not mediated by treatment completion (attending/not attending 3+ PWP sessions) at either T1 or T2. Indirect group effects were 0·007 (95% CI -0·041 to 0·054; p=0·777) and -0·002 (95% CI -0·042 to 0·038; p=0·923), respectively.

© 2023 Strauss C et al. *JAMA Psychiatry*.

eDiscussion.

At T1, a greater proportion of MBCT-SH participants scored in the non-clinical range for depression compared to CBT-SH participants, and MBCT-SH participants also showed greater reduction in generalized anxiety symptom severity. Significant differences between arms on secondary clinical outcomes (generalized anxiety symptom severity and depressive symptom severity at T2, along with wellbeing and work and social adjustment at T1 and T2) were not found; and there were non-significant differences in intervention completion between arms. In terms of mindfulness skills, between-group differences were found for the non-judging aspect of mindfulness at both time points in favor of MBCT-SH. Total mindfulness was not significantly different between arms in the intention-to-treat analysis, although it was in the per protocol analysis at T1 in favor of MBCT-SH.

Contrary to hypotheses, completion rates (defined as attending 3 or more PWP sessions) were not different between arms and treatment completion did not mediate depression outcomes at 16-weeks. This suggests that the greater clinical- and cost-effectiveness of MBCT-SH in comparison to CBT-SH was not due to greater treatment completion in the MBCT-SH arm. In terms of other potential mechanisms of action, improvement in the non-judging aspect of mindfulness were significantly different between arms in favor of MBCT-SH at post-intervention and follow-up. Non-judging refers to the capacity to accept thoughts and feelings whatever they may be and the differences between arms on this subscale is consistent with the suggestion that the clinical effectiveness of MBCT-SH in comparison to CBT-SH may be in part due to MBCT-SH fostering greater non-judgement of difficult thoughts and feelings associated with depression.

Whilst the trial was not powered to test non-inferiority, the large sample size coupled with non-significant effects on secondary outcomes being consistently in favor of MBCT-SH fits with the suggestion that practitioner-supported MBCT-SH is not inferior to CBT-SH in targeting these secondary outcomes.

© 2023 Strauss C et al. *JAMA Psychiatry*.

eFigure. PHQ-9 Scores by Trial Arm and Timepoint

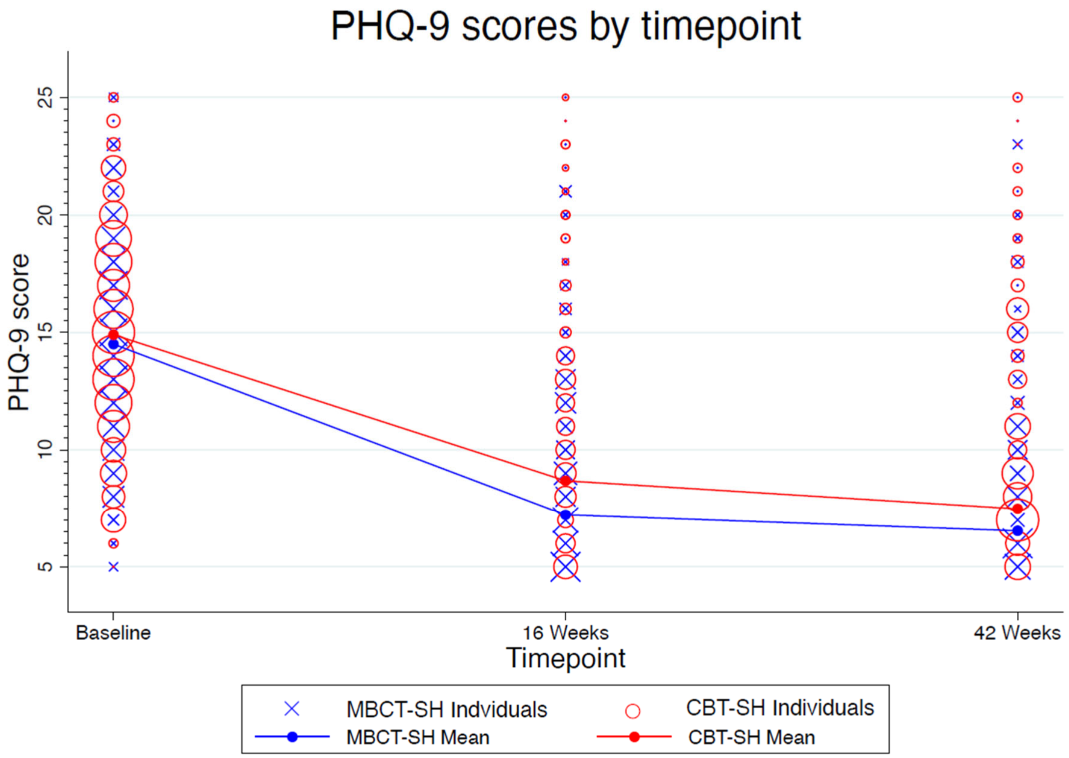

Note: Baseline means are calculated directly from the data, at 16 weeks and 42 weeks the marginal means are presented estimated from the fitted model

© 2023 Strauss C et al. *JAMA Psychiatry*.

eTable 1. Descriptive Summary for Demographic Variables Comparing PHQ-9 T1 Data Completers With T1 Data Noncompleters at 16 Weeks

Demographics	T1 Study Completers (n=309; 75%)	T1 Study Non-completers (n= 101; 25%)	All (n= 410; 100%)
**Sex******:**			
Female	201 (65%)	54 (53%)	255 (62%)
Male	108 (35%)	47 (47%)	155 (38%)
**Whether gender identity matches assignment at birth:**			
No	0 (0%)	0 (0%)	0 (0%)
Yes	309 (100%)	101 (100%)	410 (100%)
**Age** (years) Median [IQR]	32 [20]	34 [21]	32 [20]
**Ethnic Group:**			
Asian/Asian British	13 (4%)	4 (4%)	17 (4%)
Black/African/Caribbean/Black British	12 (4%)	3 (3%)	15 (4%)
Mixed	16 (5%)	4 (4%)	20 (5%)
Other	3 (1%)	2 (2%)	5 (1%)
White British/White Irish	263 (85%)	88 (87%)	351 (86%)
Prefer not to say	2 (1%)	0 (0%)	2 (<1%)
**Sexual Orientation*:**			
Bisexual	11 (4%)	7 (7%)	18 (4%)
Gay	12 (4%)	4 (4%)	16 (4%)
Heterosexual	269 (87%)	87 (86%)	356 (87%)
Lesbian	4 (1%)	1 (1%)	5 (1%)
Identify as another term	4 (1%)	1 (1%)	5 (1%)
Prefer not to say	9 (3%)	1 (1%)	10 (2%)
**Marital Status:**			
Single	127 (41%)	41 (41%)	168 (41%)
Married, cohabiting, or civil partnership	154 (50%)	54 (53%)	208 (51%)
Separated, divorced, or widowed	26 (8%)	5 (5%)	31 (8%)
Prefer Not to Say	2 (1%)	1 (1%)	3 (1%)
**Employment Status:**			
Employed	230 (74%)	78 (77%)	308 (75%)
Not looking for work - Unemployed, retired, carer, volunteer	31 (10%)	9 (9%)	40 (10%)
Student	29 (9%)	5 (5%)	34 (8%)
Unemployed & looking for work	19 (6%)	9 (9%)	28 (7%)

© 2023 Strauss C et al. *JAMA Psychiatry*.

**Highest Education Level****:			
No educational qualification	7 (2%)	1 (1%)	8 (2%)
GCSE or equivalent (qualifications taken at 16 years)	55 (18%)	27 (27%)	82 (20%)
A Levels or equivalent (qualifications taken at 18 years)	79 (26%)	37 (37%)	116 (28%)
University level education	165 (53%)	35 (35%)	200 (49%)
Prefer Not to Say	3 (1%)	1 (1%)	4 (1%)
**Symptom Severity: PHQ-9***			
Mild	154 (50%)	42 (42%)	196 (48%)
Moderate	155 (50%)	59 (58%)	214 (52%)

NB: Column percentages may not add up exactly to 100% due to rounding error.
IQR=Interquartile Range; PHQ=Patient Health Questionnaire; $\chi 2$ test or Fisher's exact tests used to compare completers and non-completers and * indicates p-values <0.15 and ** p-values <0.05

© 2023 Strauss C et al. *JAMA Psychiatry*.

eTable 2. Descriptive Summary of Baseline Clinical Measures Comparing PHQ-9 T1 Data Completers With T1 Data Noncompleters at 16 Weeks

	Completers (n=309)	Non-completers (n= 101)	All (n= 410)
	**Mean (SD) N**	**Mean (SD) N**	**Mean (SD) N**
**PHQ-9**	14·60 (4·08) 309	15·10 (4·21) 100	14·70 (4·06) 409
**GAD-7**	10·60 (4·08) 309	11·20 (4·5) 100	10·70 (4·19) 409
**SWEMWS**	10·50 (3·33) 309	10·60 (3·35) 100	10·50 (3·33) 409
**WSAS**	20·30 (6·73) 308	20·20 (6·65) 100	20·20 (6·70) 408
**FFMQ-15 Observe***	8·62 (2·75) 309	8·13 (2·36) 101	8·50 (2·66) 410
**FFMQ-15 Describe***	8·39 (2·71) 309	7·98 (2·42) 101	8·29 (2·65) 410
**FFMQ-15 Aware**	8·23 (2·09) 309	8·54 (2·43) 101	8·31 (2·18) 410
**FFMQ-15 Non-Judge**	8·24 (2·67) 309	8·15 (2·68) 100	8·22 (2·66) 409
**FFMQ-15 Non-Reacting**	7·89 (2·51) 308	8·29 (2·57) 100	7·99 (2·52) 408
**FFMQ15 Total**	32·70 (5·91) 308	32·90 (6·56) 100	32·80 (6·07) 408

Notes: *Indicates independent t-test results comparing completers and non-completers with p-values <0.1; PHQ-9= Patient Health Questionnaire; GAD-7= Generalized Anxiety Disorder; SWEMWS= Short Warwick-Edinburgh Mental Well-Being Scale; WSAS= Work and Social Adjustment Scale; FFMQ15= Five Facet Mindfulness Questionnaire

© 2023 Strauss C et al. *JAMA Psychiatry*.

eTable 3. Descriptive Summary of Clinical Outcomes, Effect Estimates, and Effect Sizes for Observed-Cases Per-Protocol Analysis

	Mean (SD) N	Mean (SD) N	Mean (SD) N	Between group difference (95% CI)		Cohen's d		P value*		P Value †	
	Baseline	16 Weeks	42 Weeks	16 Weeks	42 Weeks	16 Weeks	42 Weeks	16 Weeks	42 Weeks	16 Weeks	42 Weeks
**PHQ-9**				-1·10 (-2·25 to 0·05)	-1·01 (-2·18 to 0·16)	-0·28	-0·26	0·060	0·089	0·120	0·138
MBCT-SH	14·50 (3·84) 157	6·72 (4·77) 129	5·97 (4·72) 120								
CBT-SH	14·90 (3·92) 151	7·92 (5·11) 130	7·15 (5·13) 121								
**GAD-7**				-0·91 (-1·90 to 1 0·08)	-0·94 (-1·96 to 0·08)	-0·22	-0·23	0·072	0·070	0·164	0·149
MBCT-SH	10·10 (4·28) 157	5·38 (4·31) 129	4·72 (4·07) 120								
CBT-SH	11·40 (3·89) 151	6·60 (4·49) 130	6·07 (4·55) 121								
**SWEMWS**				0·98 (-0·12 to 2·07)	1·11 (-0·01 to 2·22)	0·30	0·34	0·080	0·053	0·177	0·079
MBCT-SH	10·30 (3·44) 157	16·30 (4·53) 129	16·9 (4·61) 120								
CBT-SH	10·90 (3·08) 151	15·60 (4·75) 130	15·9 (4·76) 121								
**WSAS**				-1·11 (-2·90 to 0·69)	-1·18 (-3·01 to 0·65)	-0·17	-0·18	0·226	0·207	0·357	0·247
MBCT-SH	21·30 (6·31) 157	11·30 (7·89) 129	10·5 (7·99) 120								
CBT-SH	19·90 (6·89) 150	11·90 (7·69) 130	11·6 (7·75) 120								
**FFMQ-15 Observe**				0·33 (-0·19 to 0·84)	0·49 (-0·04 to 1·02)	0·12	0·18	0·217	0·071	0·480	0·184
MBCT-SH	8·31 (2·94) 157	9·88 (2·41) 129	10·2 (2·19) 120								
CBT-SH	8·64 (2·46) 151	9·77 (2·44) 130	9·93 (2·45) 120								
**FFMQ-15 Describe**				0·45 (-0·08 to 0·97)	0·25 (-0·29 to 0·79)	0·17	0·09	0·094	0·367	0·156	0·381
MBCT-SH	8·10 (2·72) 157	10.00 (2·45) 129	9·97 (2·43) 120								

© 2023 Strauss C et al. *JAMA Psychiatry*.

CBT-SH	8·48 (2·64)<br>151	9·68 (2·49)<br>130	9·82 (2·77)<br>120								
**FFMQ-15 Aware**				-0·10 (-0·59 to 0·39)	-0·13 (-0·64 to 0·37)	-0·05	-0·06	0·692	0·601	0·626	0·546
MBCT-SH	8·22 (2·15)<br>157	9·13 (2·08)<br>129	9·26 (2·33)<br>120								
CBT-SH	7·95 (2·11)<br>151	9·13 (2·18)<br>130	9·23 (2·40)<br>120								
**FFMQ-15 Non-Judge**				0·84 (0·22 to 1·46)	0·84 (0·20 to 1·48)	0·33	0·33	0·008	0·010	0·016	0·008
MBCT-SH	8·13 (2·52)<br>157	10·7 (2·81)<br>129	11·3 (2·66)<br>120								
CBT-SH	8·13 (2·65)<br>151	9·92 (2·76)<br>130	10·3 (2·84)<br>120								
**FFMQ-15 Non-Reacting**				0·34 (-0·20 to 0·88)	0·37 (-0·18 to 0·93)	0·13	0·14	0·220	0·188	0·614	0·557
MBCT-SH	8·04 (2·69)<br>156	9·73 (2·22)<br>129	9·82 (2·22)<br>120								
CBT-SH	7·98 (2·48)<br>151	9·47 (2·59)<br>130	9·49 (2·37)<br>120								
**FFMQ-15 Total**				1·56 (0·02 to 3·11)	1·31 (-0·27 to 2·89)	0·25	0·21	0·047	0·105	0·174	0·234
MBCT-SH	32·50 (6·01)<br>156	39·60 (6·77)<br>129	40·40 (6·55)<br>120								
CBT-SH	32·50 (6·37)<br>151	38·20 (7·08)<br>130	38·90 (7·68)<br>120								

Notes: MBCT-SH=Mindfulness Based Cognitive Therapy-Self Help· CBT-SH=Cognitive Behavioural Therapy-Self Help· *p values reported are note: the treatment group-time interaction contrasts of marginal linear predictions for observed data· †p values reported are the treatment group-time interaction contrasts of marginal linear predictions for imputed data· PHQ-9= Patient Health Questionnaire; GAD-7= Generalized Anxiety Disorder; SWEMWS= Short Warwick-Edinburgh Mental Well-Being Scale; WSAS= Work and Social Adjustment Scale; FFMQ15= Five Facet Mindfulness Questionnaire·

© 2023 Strauss C et al. *JAMA Psychiatry*.

eTable 4. Between-Group Effect Estimates and Effect Sizes for Modified Intention-to-Treat Analysis With Observed Cases and Multiple Imputation

		Observed Cases Analysis							Multiple Imputation						
Clinical Outcome	Timepoint	Between group difference	SE	z	P>|z|	95% LCL	95% UCL	Cohen's d	Between group difference	SE	t	P>|t|	95% LCL	95% UCL	Cohen's d
PHQ-9	16 Weeks	-1·46	0·56	-2·6	0·009	-2·55	-0·36	-0·36	-1·30	0·55	-2·36	0·019	-2·39	-0·22	-0·32
	42 Weeks	-0·94	0·57	-1·66	0·097	-2·05	0·17	-0·23	-0·90	0·54	-1·66	0·097	-1·95	0·16	-0·22
GAD-7	16 Weeks	-0·95	0·48	-1·99	0·047	-1·88	-0·01	-0·23	-0·84	0·48	-1·76	0·080	-1·78	0·10	-0·20
	42 Weeks	-0·79	0·48	-1·65	0·100	-1·74	0·15	-0·19	-0·74	0·48	-1·54	0·126	-1·68	0·21	-0·18
SWEMBS	16 Weeks	0·97	0·52	1·87	0·061	-0·05	1·99	0·29	0·74	0·52	1·42	0·158	-0·29	1·77	0·22
	42 Weeks	0·70	0·53	1·33	0·182	-0·33	1·73	0·21	0·71	0·52	1·37	0·173	-0·31	1·72	0·21
WSAS	16 Weeks	-1·39	0·85	-1·63	0·103	-3·05	0·28	-0·21	-1·26	0·92	-1·36	0·175	-3·08	0·57	-0·19
	42 Weeks	-1·00	0·86	-1·16	0·247	-2·69	0·69	-0·15	-1·01	0·87	-1·16	0·247	-2·73	0·71	-0·15
FFMQ15	16 Weeks	0·27	0·24	1·12	0·263	-0·20	0·75	0·10	0·12	0·25	0·48	0·629	-0·37	0·61	0·05
Observe	42 Weeks	0·16	0·25	0·66	0·507	-0·32	0·65	0·06	0·11	0·23	0·47	0·642	-0·35	0·56	0·04
FFMQ15	16 Weeks	0·37	0·25	1·50	0·134	-0·11	0·85	0·14	0·30	0·25	1·20	0·231	-0·19	0·80	0·11
Describing	42 Weeks	-0·02	0·25	-0·07	0·945	-0·51	0·47	-0·01	0·06	0·24	0·25	0·806	-0·41	0·52	0·02
FFMQ15	16 Weeks	-0·11	0·23	-0·49	0·627	-0·56	0·33	-0·05	-0·13	0·22	-0·60	0·547	-0·55	0·29	-0·06
Awareness	42 Weeks	-0·22	0·23	-0·96	0·338	-0·68	0·23	-0·10	-0·24	0·22	-1·06	0·290	-0·67	0·20	-0·11
FFMQ15	16 Weeks	0·71	0·29	2·48	0·013	0·15	1·28	0·27	0·71	0·32	2·23	0·027	0·08	1·33	0·27
Non Judge	42 Weeks	0·66	0·29	2·24	0·025	0·08	1·23	0·25	0·73	0·32	2·29	0·023	0·10	1·35	0·27
FFMQ15	16 Weeks	0·21	0·26	0·81	0·417	-0·29	0·71	0·08	0·09	0·27	0·31	0·755	-0·45	0·62	0·03
Non React	42 Weeks	0·20	0·26	0·77	0·440	-0·31	0·71	0·08	0·09	0·26	0·33	0·739	-0·42	0·60	0·03
FFMQ15	16 Weeks	1·22	0·72	1·69	0·091	-0·20	2·64	0·20	0·90	0·78	1·16	0·248	-0·63	2·43	0·15
Total*	42 Weeks	0·62	0·74	0·84	0·402	-0·83	2·06	0·10	0·60	0·74	0·82	0·414	-0·85	2·06	0·10

© 2023 Strauss C et al. *JAMA Psychiatry*.

Notes: Between group difference estimates adjusted for baseline depression severity category on PHQ-9 & Site· PHQ= Patient Health Questionnaire; GAD= Generalized Anxiety Disorder; SWEMWS= Short Warwick-Edinburgh Mental Well-Being Scale; WSAS= Work and Social Adjustment Scale; FFMQ= Five Facet Mindfulness Questionnaire; *Total excludes the Observing item; SE = Standard Error; LCL = lower confidence limit; UCL = upper confidence limit

© 2023 Strauss C et al. *JAMA Psychiatry*.

eTable 5. Between-Group Effect Estimates and Effect Sizes for Per-Protocol Analysis With Observed Cases and Multiple Imputation

		Per-Protocol Analysis							Multiple Imputation						
**Clinical Outcome**	**Timepoint**	**Between group difference**	**SE**	**z**	**P>|z|**	**95% LCL**	**95% UCL**	**Cohen's d**	**Between group difference**	**SE**	**t**	**P>|t|**	**95% LCL**	**95% UCL**	**Cohen's d**
PHQ-9	16 Weeks	-1·1	0·59	-1·88	0·06	-2·25	0·04	-0·28	-0·91	0·58	-1·56	0·120	-2·06	0·24	-0·23
	42 Weeks	-1·01	0·60	-1·70	0·089	-2·18	0·16	-0·26	-0·88	0·60	-1·48	0·138	-2·05	0·29	-0·23
GAD-7	16 Weeks	-0·91	0·51	-1·8	0·072	-1·91	0·08	-0·22	-0·70	0·51	-1·39	0·164	-1·70	0·29	-0·17
	42 Weeks	-0·94	0·52	-1·81	0·07	-1·96	0·08	-0·23	-0·76	0·52	-1·45	0·149	-1·78	0·27	-0·18
SWEMBS	16 Weeks	0·98	0·56	1·75	0·08	-0·12	2·07	0·30	0·74	0·55	1·35	0·177	-0·34	1·83	0·23
	42 Weeks	1·11	0·57	1·94	0·053	-0·01	2·22	0·34	1·00	0·57	1·76	0·079	-0·12	2·11	0·30
WSAS	16 Weeks	-1·11	0·91	-1·21	0·226	-2·9	0·69	-0·17	-0·91	0·99	-0·92	0·357	-2·86	1·04	-0·14
	42 Weeks	-1·18	0·93	-1·26	0·207	-3·01	0·65	-0·18	-1·19	1·02	-1·16	0·247	-3·21	0·83	-0·18
FFMQ15	16 Weeks	0·33	0·26	1·24	0·217	-0·19	0·84	0·12	0·19	0·27	0·71	0·48	-0·34	0·71	0·07
Observe	42 Weeks	0·49	0·27	1·81	0·071	-0·04	1·02	0·18	0·35	0·26	1·33	0·184	-0·17	0·86	0·13
FFMQ15	16 Weeks	0·45	0·27	1·68	0·094	-0·08	0·97	0·17	0·38	0·27	1·42	0·156	-0·15	0·91	0·14
Describing	42 Weeks	0·25	0·27	0·9	0·367	-0·29	0·78	0·09	0·23	0·26	0·88	0·381	-0·29	0·75	0·09
FFMQ15	16 Weeks	-0·1	0·25	-0·4	0·692	-0·59	0·39	-0·05	-0·12	0·25	-0·48	0·626	-0·62	0·37	-0·06
Awareness	42 Weeks	-0·13	0·26	-0·52	0·601	-0·64	0·37	-0·06	-0·15	0·25	-0·61	0·546	-0·65	0·35	-0·07
FFMQ15	16 Weeks	0·84	0·32	2·66	0·008	0·22	1·46	0·33	0·79	0·33	2·42	0·016	0·15	1·43	0·31
Non Judge	42 Weeks	0·84	0·32	2·59	0·01	0·2	1·48	0·33	0·85	0·32	2·65	0·008	0·22	1·48	0·33
FFMQ15	16 Weeks	0·34	0·27	1·23	0·22	-0·2	0·87	0·13	0·15	0·30	0·51	0·614	-0·43	0·73	0·06
Non React	42 Weeks	0·37	0·28	1·32	0·188	-0·18	0·93	0·14	0·18	0·31	0·59	0·557	-0·43	0·79	0·07
FFMQ15	16 Weeks	1·56	0·79	1·99	0·047	0·02	3·11	0·25	1·15	0·85	1·36	0·174	-0·51	2·82	0·19
Total*	42 Weeks	1·31	0·81	1·62	0·105	-0·27	2·89	0·21	1·06	0·89	1·19	0·234	-0·69	2·81	0·17

© 2023 Strauss C et al. *JAMA Psychiatry*.

Notes: Between group difference estimates adjusted for baseline depression severity category on PHQ-9 & Site· PHQ= Patient Health Questionnaire; GAD= Generalized Anxiety Disorder; SWEMWS= Short Warwick-Edinburgh Mental Well-Being Scale; WSAS= Work and Social Adjustment Scale; FFMQ= Five Facet Mindfulness Questionnaire; *Total excludes the Observing item; SE = Standard Error; LCL = lower confidence limit; UCL = upper confidence limit

© 2023 Strauss C et al. *JAMA Psychiatry*.

eTable 6. Between-Group PHQ-9 Effect Estimates and Effect Sizes by Sex, Ethnicity, and Baseline Medication Use Using Observed Cases and Multiple Imputation

		PHQ-9 Observed Cases Analysis							PHQ-9 Multiple Imputation						
Participant characteristic	**Timepoint**	**Between group difference**	**SE**	**z**	**P>|z|**	**95% LCL**	**95% UCL**	**Cohen's d**	**Between group difference**	**SE**	**t**	**P>|t|**	**95% LCL**	**95% UCL**	**Cohen's d**
**Sex**															
Female	16 Weeks	-1·79	0·69	-	-	-3·15	-0·43	-0·44	-1·73	0·73	-	-	-3·16	-0·29	-0·43
Male	16 Weeks	-0·79	0·95	-	-	-2·65	1·08	-0·19	-0·64	1·01	-	-	-2·63	1·36	-0·16
Female	42 Weeks	-1·12	0·70	-	-	-2·50	0·26	-0·28	-1·29	0·67	-	-	-2·61	0·03	-0·32
Male	42 Weeks	-0·61	0·96	-	-	-2·49	1·28	-0·15	-0·52	0·98	-	-	-2·45	1·42	-0·13
**Ethnicity**															
Ethnic Minority	16 Weeks	-1·92	1·11	-	-	-4·10	0·26	-0·47	-1·86	1·12	-	-	-4·05	0·33	-0·46
White British	16 Weeks	-1·34	0·65	-	-	-2·61	-0·07	-0·33	-1·10	0·65	-	-	-2·38	0·17	-0·27
Ethnic Minority	42 Weeks	-0·99	1·13	-	-	-3·22	1·23	-0·24	-0·90	1·10	-	-	-3·06	1·26	-0·22
White British	42 Weeks	-0·93	0·65	-	-	-2·21	0·35	-0·23	-1·00	0·67	-	-	-2·32	0·32	-0·25
**Medication***															
No-Medication	16 Weeks	-1·47	0·85	-	-	-3·14	0·20	-0·36	-1·50	0·89	-	-	-3·27	0·26	-0·37
Yes-Medication	16 Weeks	-0·86	0·87	-	-	-2·55	0·84	-0·21	-1·14	0·95	-	-	-3·03	0·75	-0·28
No-Medication	42 Weeks	-1·58	0·82	-	-	-3·18	0·03	-0·39	-1·63	0·80	-	-	-3·22	-0·06	-0·40
Yes-Medication	42 Weeks	-0·09	0·85	-	-	-1·75	1·57	-0·02	-0·49	0·88	-	-	-2·23	1·24	-0·12

Note: Baseline Patient Health Questionnaire (PHQ-9) SD=4.06 for calculating Cohen's ds; test statistics and p-values omitted as this clinical trial was not powered to carry out subgroup analyses; *Medication had 29% missing data; SE = Standard Error; LCL = lower confidence limit; UCL = upper confidence limit

© 2023 Strauss C et al. *JAMA Psychiatry*.

eTable 7. Proportion of Participants in the Nonclinical Range (PHQ-9 score of 0-9) for Depressive Symptoms at 16 and 42 Weeks

	**Baseline**				**16 weeks**				**42 weeks**			
	CBT-SH		MBCT-SH		CBT-SH		MBCT-SH		CBT-SH		MBCT-SH	
PHQ-9 Categories	Count	%	Count	%	Count	%	Count	%	Count	%	Count	%
0-9 (non-clinical)	22	10·68	25	12·25	92	62·59	112	74·17	109	75·69	111	77·62
10-14 (moderate)	75	36·41	79	38·73	43	29·25	32	21·19	19	13·19	24	16·78
15-19 (moderately severe)	82	39·81	78	38·24	12	8·16	7	4·64	16	11·11	8	5·59
20+ (severe)	27	13·11	22	10·78	0	0	0	0	0	0	0	0
Total	206	100	204	89·22	147	100	151	100	144	100	143	100

NB: MBCT-SH=Mindfulness Based Cognitive Therapy-Self Help. CBT-SH=Cognitive Behavioural Therapy-Self Help· PHQ-9 = Patient Health Questionnaire

© 2023 Strauss C et al. *JAMA Psychiatry*.

eTable 8. PWP Session Adherence

PWP session adherence	MBCT-SH	CBT-SH
	**Number of participants (%)**	**Number of participants (%)**
Participants who did not initiate PWP sessions (0 sessions)	7 (3·4%)	15 (7·3%)
Participants who initiated PWP Session	197 (96·6%)	191 (92·7%)
Mean number of sessions attended	4·5	4·3
Median number of sessions attended	6	6
Standard Deviation Sessions attended	2·0	2·2
Completed 3 or more sessions (‘completers’)	157 (77·0%)	151 (73·3%)

NB: MBCT-SH=Mindfulness Based Cognitive Therapy-Self Help. CBT-SH=Cognitive Behavioural Therapy-Self Help· PWP=Psychological Wellbeing Practitioner

© 2023 Strauss C et al. *JAMA Psychiatry*.

eTable 9. Lasting Negative Effects by Study Arm

Whether lasting Bad Effect	CBT-SH Count	CBT-SH %	MBCT-SH Count	MBCT-SH %	Total Count	Total %
No (Scored 3,4,5)	141	96	138	95	279	95
Yes (Scored 1,2)	6	4	8	5	14	5
Total	147	100	146	100	293	100

NB: MBCT-SH=Mindfulness Based Cognitive Therapy-Self Help· CBT-SH=Cognitive Behavioural Therapy-Self Help· PWP=Psychological Wellbeing Practitioner

© 2023 Strauss C et al. *JAMA Psychiatry*.

eAppendix 1. Further Details of Health Economic Evaluation

Methods

Intervention costs

The cost of the two interventions (MBCT-SH and CBT-SH) were calculated using a micro-costing approach[5] based on the cost of training and the cost of PWPs, which included salary[6] and relevant on-costs, overheads and indirect time· The same PWP unit cost was applied to both the MBCT-SH and CBT-SH group since these were both implemented by PWPs· Data on the number and duration of PWP contacts in the MBCT-SH and CBT-SH arms were recorded using a proforma completed by PWPs· Data on indirect time, including preparation and supervision, were collected directly from the PWPs·

Training costs were calculated as outlined in Tables SM7 and SM8· Training was provided in group sessions· Eight training courses were provided to 47 PWPs in total with varying numbers of PWPs in each training course· It was estimated that the total training cost per depression related session was £0·47· This was only applied to MBCT-SH contacts as the training was specifically for MBCT-SH, as PWPs are already trained in CBT-SH·

The cost of providing MBCT-SH and CBT-SH included direct and indirect contact· Direct contact was costed as £34 per hour based on band 5 clinical psychology assistant practitioners[6]. Data on indirect PWP time indicated that direct to indirect time occurred at a ratio of 1:0·82 so, direct contact costs were multiplied by 1·82 to account for additional time· Therefore, the cost of implementation was £1·03 per minute (£34 per hour direct cost * 1·82 for indirect contact = £61·88 per hour)·

Other health and social care costs

Resource use data on all other health and social care services were collected using a version of the Adult Service Use Schedule (AD-SUS) adapted for the study and for online completion· Nationally applicable published unit costs were applied to service use data and taken from various sources including Unit Costs of Health and Social Care[6]. NHS reference costs[7], Prescription Cost Analysis[8] and other published literature[9]. Unit costs were inflated where necessary using published guidance[10]. All costs are reported in UK pounds sterling at 2017-2018 prices· Discounting was not relevant as the follow-up did not exceed 12-months· Unit costs of all other health and social care services are summarised in Table SM9·

© 2023 Strauss C et al. *JAMA Psychiatry*.

eTable 10: Psychological wellbeing practitioner training costs

Components	Details	Unit cost	Total cost per training course	Total cost of all training	Cost per therapist	Cost per depression related session	Source
**Trainer's time**	One band 8d psychologist for 6 hours per day for 2 days· One band 8b psychologist for 6 hours per day for 2 days· One band 5 nurse for 6 hours per day for 1 day· 8 training courses were provided to 47 therapists·	Band 8d: £106 per hour; Band 8b: £75 per hour; Band 5: £34 per hour	£2,376	£19,008	£404	**-**	Curtis & Burns, 2018
**Trainee's time**	One band 5 for 6 hours per day for 2 days·	Band 5: £34 per hour	-	£19,176	£408	**-**	Curtis & Burns, 2018
**Total training cost per therapist**		**-**	**-**	-	£812	**-**	
**Total training cost per depression related session***		**-**	**-**	**-**	**-**	**£0·47**	

*Based on 1,712·34 depression related session over 3 years: see below for assumptions

eTable 11: Psychological wellbeing practitioner training cost assumptions

Assumptions	Details	Source
**Working days per annum**	210 days	Curtis, L. & Burns, A. (2018)[6]
**Average patient contacts per day**	9 contacts	Nolan, G. (2009)[11]
**Percentage of caseload with depression***	30·2%	Health and Social Care Information Centre (2014)[12]. Based on entering treatment by provisional diagnosis.
**Training valid over years**	3 years	Ekers, D. et al. (2011)[13]
**Number of depression related sessions**	1,712·34 sessions	Calculated as 210x9x3x30·2

* Depression or mixed anxiety and depression

© 2023 Strauss C et al. *JAMA Psychiatry*.

eTable 12: Unit costs of health and social care services

Resource	Unit	Cost (£)	Source	Notes
**Hospital services**				
Overnight stay for mental health	Night	402	2	Non Elective Inpatients section - average of cost per bed day for relevant mental health inpatient codes
Overnight stay for physical health	Night	557	2	Non Elective Inpatients - average of all contacts cost per bed day
Hospital appointment for mental health	Appointment	246	2	Adult mental illness in outpatient attendances
Hospital appointment for physical health	Appointment	125	2	Average of all outpatient attendances
A&E	Attendance	160	2	Unit Costs of accident and emergency
Ambulance	Event	252	2	See and treat and convey in Ambulance tab
**Community services**				
GP at surgery	Contact	31	1	Per surgery consultation lasting 9·22 minutes, including direct care staff costs, without qualifications
GP at home	Contact	71	1	Based on £31 per 9·22 minute consultation, plus data on home visits duration of 9·22 minutes assuming similar time plus 12 minutes travel time[14].
GP on telephone	Contact	24	1	Based on £31 per 9·22 minute consultation, plus data on telephone duration of 7·1 minutes[14].
Practice nurse	Contact	9	1	Based on £36 per hour excluding qualifications assuming 15·5 min appointment[15].
NHS community mental health professional	Contact	160	2	Other Mental Health Specialist Teams, Adult and Elderly in Mental Health tab
**Psychological and talking therapies**				
Individual face-to-face psychological and talking therapies	Contact	203	4	Assume high intensity IAPT; Based on £177 at 2009/10 prices, inflated up to 2017/8 prices using the GDP price deflator using source 5
Group psychological and talking therapies	Contact	16	1	Cost per session per person attending a group of behavioural activation delivered by a non-specialist, excluding qualifications
Individual telephone psychological and talking therapies	Contact	114	4	Assume low intensity IAPT; Based on £99 at 2009/10 prices, inflated up to 2017/8 prices using the GDP price deflator using source 5
**Medication**				
Any medication	Item	8	3	Net Ingredient Cost per item, assuming each item last 1 month

© 2023 Strauss C et al. *JAMA Psychiatry*.

Data analysis

The economic evaluation was conducted covering the period from baseline to final follow-up (42 weeks post-randomisation) based on those with complete data needed for the economic evaluation· Costs and outcomes were compared and presented in terms of mean differences and 95% confidence intervals obtained by non-parametric bootstrap regression to account for the non-normal distribution commonly found in economic data[16]. Cost-effectiveness was assessed through the calculation of incremental cost-effectiveness ratios explored in terms of quality-adjusted life years (QALYs) calculated from the EQ-5D-5L and using the area under the curve approach[17]. As NICE recommend mapping EQ-5D-5L data to the EQ-5D-3L value set[18], this was conducted· Appropriate utility weights were attached to health states[19].

Uncertainty was explored using cost-effectiveness planes and cost-effectiveness acceptability curves based on the net-benefit approach[20,21]. These curves are an alternative to confidence intervals around ICERs and show the probability that one intervention is cost-effective compared to the other, for a range of values that a decision maker would be willing to pay for an additional unit of an outcome· All economic analyses included relevant baseline variables in line with the clinical analysis (site and PHQ-9) plus the baseline variable of interest, baseline utility and follow-up time, to provide a more relevant treatment-effect estimate[22].

A sensitivity analysis was carried out with missing total costs and outcomes imputed using multiple imputation by chained equations· In addition, due to concerns regarding large variations in cost, a decision was made post-hoc to conduct an additional sensitivity analysis which controlled for (removed) outliers and influential observations using a combination of a DFBETA and qualified box-plot outliers[23]. For this, an observation was deemed to be a box-plot outlier if the log cost was greater than the third quartile plus 1·5 times the interquartile range (ln(cost)>Q3+1·5*IQR), and an observation was deemed to be influential if the DFBETA was greater than +/- 0·15·

Results

Response rates

A total of 410 participants were randomised to MBCT-SH (n=204), and CBT-SH (n=206)· Seventy-one percent (292/410) of participants had intervention, AD-SUS and EQ-5D-5L data at T0, T1 and T2 plus baseline covariate data, allowing them to be included in the complete case economic analyses at the T2 analysis point· This was 71% (145/204) in the MBCT-SH group and 71% (147/206) in the CBT-SH group· Average follow-up time was 44·56 weeks in the group as a whole (3·00 SD, range 39·78 to 60·41), 44·25 weeks in the MBCT-SH group (39·78 to 55·96) and 44·86 weeks in the CBT-SH group (3·21 SD, range 39·87 to 60·41)·

Table SM10 compares the baseline characteristics of the full sample with those participants with all economic data for the economic analysis· The sample with all data necessary for inclusion in the complete-case economic analysis were very similar to the full sample in terms of sex, age, baseline utility, baseline PHQ-9 and baseline costs·

© 2023 Strauss C et al. *JAMA Psychiatry*.

eTable 13: Baseline characteristics of full sample and those with full economic data

Characteristic	Full sample (n=410)	Full data for economic analysis (n=292)
Sex, n (%)		
Male	155 (38%)	104 (35%)
Female	255 (62%)	189 (65%)
Age (years), mean (SD)	36 (13)	36 (13)
Baseline utility, mean (SD)	0·72 (0·15)	0·72 (0·15)
Baseline PHQ-9, mean (SD)	14·69 (4·06)	14·72 (3·92)
Baseline cost (£), mean (SD)	406 (736)	396 (776)

Resource use

Service use over the period from baseline to 42-week follow-up is presented in Table SM11· On average, the MBCT-SH group used fewer of all hospital services, particularly outpatient contacts for mental health (mean 2·0 vs· 4·1 contacts), had fewer GP contacts (mean 6·3 vs· 9·7 contacts), and fewer face-to-face non-intervention psychological talking therapy contacts (mean 6·0 vs· 8·4 contacts) than the CBT-SH group, although they had more group psychological talking therapy contacts (mean 5·7 vs· 3·7) and more CMHT contacts than the CBT-SH group (mean 5·6 vs· 4·5)· In addition, a lower proportion of the MBCT-SH group used psychotropic medication (45%) than the CBT-SH group (53%)·

In terms of use of MBCT-SH and CBT-SH, a similar proportion of the two group received intervention sessions (MBCT-SH 97%, CBT-SH 93%). The average number of sessions attended (MBCT-SH 4·5, CBT-SH 4·3) were also similar·

Costs

Total health and social care costs, intervention costs, and total costs for the complete case data are presented in the paper· Table SM12 shows total costs (health and social care plus intervention costs) based on the outlier sensitivity analysis and missing data analysis using imputed data which supported the complete case analysis·

© 2023 Strauss C et al. *JAMA Psychiatry*.

Outcomes

Table SM13 reports EQ-5D-5L based utility at baseline, 16-week and 42-week follow-up plus QALYs from baseline to 42-weeks· Utility values at baseline, 16-weeks and 42-weeks were similar in both groups· There were no significant differences in utility at each time point, or in QALYs from baseline to 42-week follow-up· This was supported by the outlier sensitivity analysis and missing data analysis using imputed data·

eTable 14: Resource use between baseline and 42-week follow-up

Resource	Unit	MBCT-SH (n=145)		CBT-SH (n=147)	
		Number (%) of users	Mean (S·D·)	Number (%) of users	Mean (S·D·)
Hospital					
Inpatient for mental health	Nights	0 (0%)	-	0 (0%)	-
Inpatient for physical health	Nights	6 (4%)	3·50 (3·73)	9 (6%)	4·89 (8·07)
Outpatient for mental health	Attendance	4 (3%)	2·00 (1·15)	10 (7%)	4·10 (3·96)
Outpatient for physical health	Attendance	53 (37%)	3·00 (2·60)	54 (37%)	3·65 (4·09)
Accident & Emergency	Contacts	21 (14%)	1·57 (1·25)	29 (20%)	1·97 (1·40)
Ambulance care or transport	Contacts	10 (7%)	1·70 (1·16)	7 (5%)	1·86 (1·07)
Community					
GP at surgery	Contacts	112 (77%)	3·18 (2·42)	112 (76%)	4·02 (4·81)
GP at home	Contacts	1 (1%)	1·00 (-)	3 (2%)	3·00 (2·00)
GP by phone	Contacts	60 (41%)	2·13 (1·33)	70 (48%)	2·67 (2·35)
Community nurse	Contacts	56 (39%)	2·36 (2·52)	58 (39%)	1·74 (1·02)
Community Mental Health Team (CMHT)	Contacts	10 (7%)	5·60 (7·49)	19 (13%)	4·47 (2·86)
Psychological and talking therapies*	Contacts				
Individual face-to-face	Contacts	24 (17%)	6·00 (4·00)	48 (33%)	8·42 (10·69)
Group therapy	Contacts	10 (7%)	5·70 (2·75)	11 (7%)	3·73 (3·55)
Individual telephone	Contacts	20 (14%)	4·40 (3·39)	28 (19%)	4·43 (4·45)
Psychotropic medication	Yes/No	65 (45%)	-	78 (53%)	-

*Excludes MBCT-SH and CBT-SH provided through the trial

© 2023 Strauss C et al. *JAMA Psychiatry*.

eTable 15: Total costs per participant between baseline and 42-week follow-up for main analysis and sensitivity analyses

Costs	MBCT-SH		CBT-SH			
	Valid n	Mean cost (SD) (£)	Valid n	Mean cost (SD) (£)	Unadjusted mean difference (95% CI, p-value) (£)	Adjusted mean difference (95% CI, p-value) # (£)
Complete case analysis						
Total health and social care costs	145	944 (1102)	147	1571 (2754)	-627 (-1109 to -145, 0·011)	-526 (-908 to -144, 0·007)
Sensitivity analysis with outliers removed						
Total health and social care costs	144	901 (981)	143	1229 (1328)	-328 (-597 to -60, 0·017)	-321 (-583 to -60, 0·016)
Sensitivity analysis with imputed data						
Total health and social care costs	204	955 (1113)	206	1527 (2626)	-571 (-1008 to -135, 0·010)	-525 (-938 to -111, 0·013)

Adjusted by baseline cost, baseline EQ-5D-5L utility, baseline PHQ-9, site and follow-up time·

eTable 16: Economic outcomes for main analysis and sensitivity analyses

Outcomes	MBCT-SH		CBT-SH			
	Valid n	Mean (SD)	Valid n	Mean (SD)	Unadjusted mean difference (95% CI, p-value)	Adjusted mean difference (95% CI, p-value) #
Complete case data						
EQ-5D-5L utility at baseline	145	0·72 (0·15)	147	0·71 (0·15)	0·01 (-0·03 to 0·04, 0·717)	0·00 (-0·03 to 0·03, 0·972)
EQ-5D-5L utility at 16-week follow-up*	129	0·78 (0·17)	131	0·76 (0·16)	0·01 (-0·03 to 0·05, 0·567)	0·00 (-0·03 to 0·04, 0·678)
EQ-5D-5L utility at 42-week follow-up	145	0·80 (0·16)	147	0·78 (0·17)	0·02 (-0·02 to 0·05, 0·382)	0·02 (-0·01 to 0·06, 0·243)
QALYs from baseline to 42-week follow-up	145	0·65 (0·13)	147	0·65 (0·12)	0·00 (-0·03 to 0·03, 0·820)	0·01 (-0·01 to 0·03, 0·484)
Sensitivity analysis with outliers removed						
QALYs from baseline to 42-week follow-up	144	0·65 (0·13)	143	0·66 (0·12)	-0·00 (-0·03 to 0·03, 0·834)	0·01 (-0·01 to 0·02, 0·617)
Sensitivity analysis with imputed data						
QALYs from baseline to 42-week follow-up	204	0·65 (0·12)	206	0·65 (0·13)	0·00 (-0·02 to 0·02, 0·960)	0·01 (-0·01 to 0·02, 0·238)

Adjusted by baseline utility, baseline PHQ-9, site and follow-up time (except baseline utility which is adjusted by baseline PHQ-9 and site only).
*Some people were missed at 16-week follow-up so QALYs estimated using baseline and 42-week EQ-5D-5L utility scores only

© 2023 Strauss C et al. *JAMA Psychiatry*.

Cost-effectiveness analysis

The cost-effectiveness plane based on the complete case data (eFigure 2) shows a greater proportion of scatter points lie to the right of the vertical axis (replications where MBCT-SH is more effective than CBT-SH) and below the horizontal axis (replications where the MBCT-SH group are less expensive than the CBT-SH group). Findings were similar for the outlier sensitivity analysis and missing data analysis using imputed data (eFigures 3-4)·

eFigure 2: Cost-effectiveness plane for MBCT-SH versus CBT-SH at 42-week follow-up based on complete cases

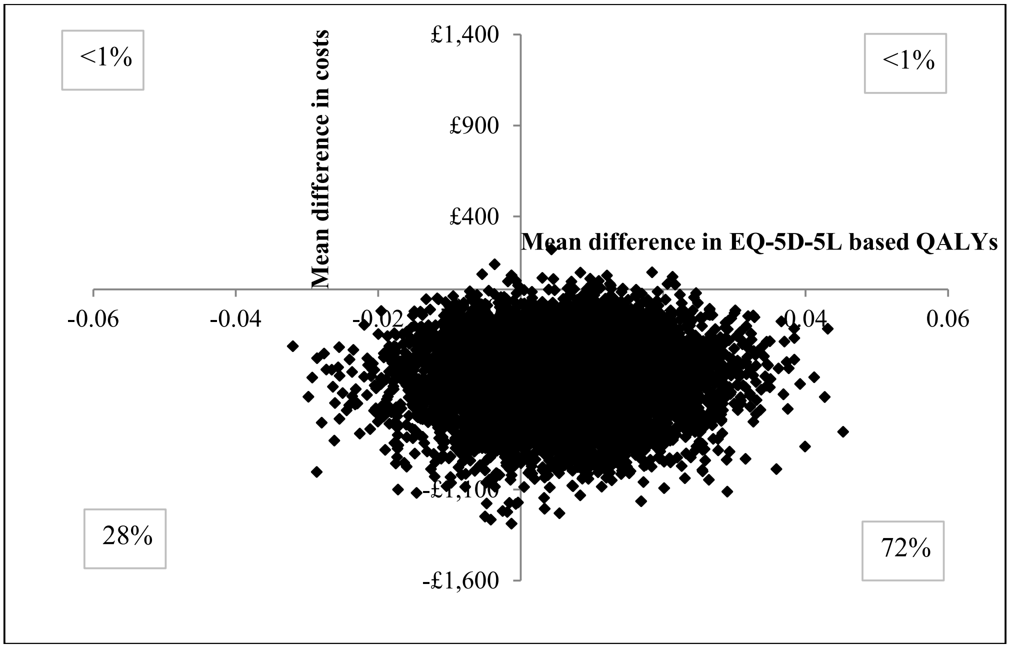

© 2023 Strauss C et al. *JAMA Psychiatry*.

eFigure 3: Cost-effectiveness plane for MBCT-SH versus CBT-SH at 42-week follow-up based on sensitivity analysis with outliers removed

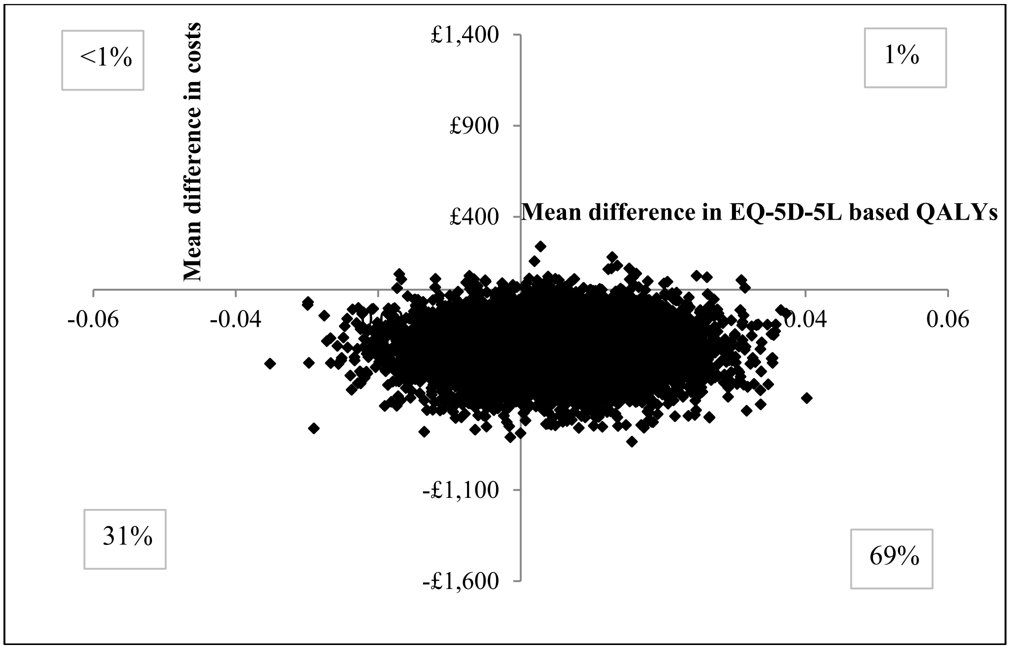

© 2023 Strauss C et al. *JAMA Psychiatry*.

eFigure 4: Cost-effectiveness plane for MBCT-SH versus CBT-SH at 42-week follow-up based on sensitivity analysis with imputed data

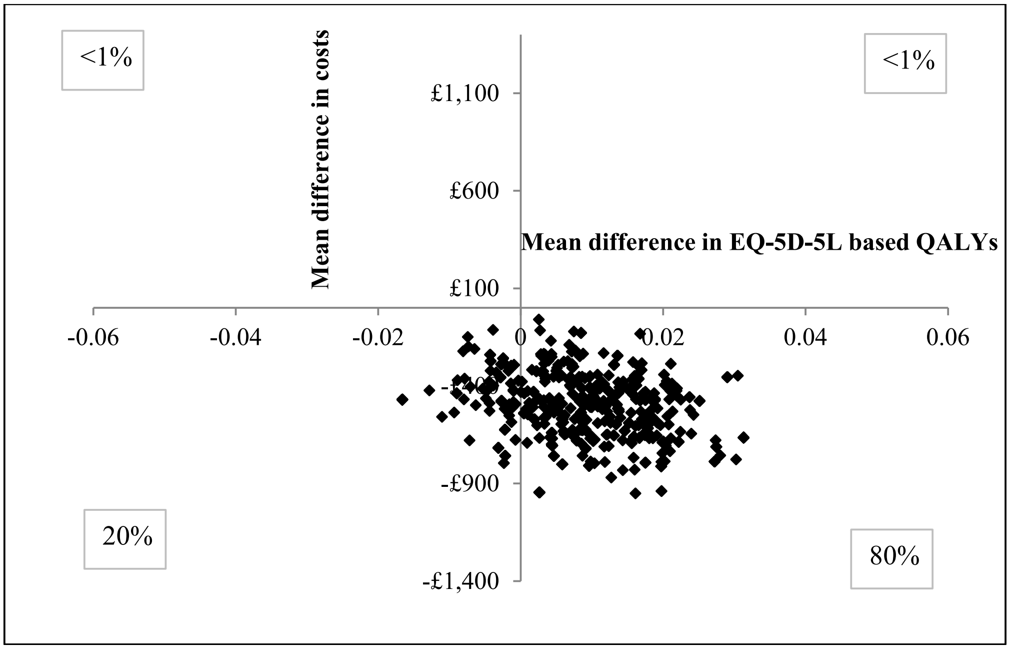

The cost-effective acceptability curve based on the complete case data (presented in the main paper) suggested that MBCT-SH had a probability of being cost effective of above 95% at the NICE willingness to pay level of £20,000 to £30,000 compared to CBT-SH· Findings were similar for the outlier sensitivity analysis and missing data analysis using imputed data (eFigures 5-6).

© 2023 Strauss C et al. *JAMA Psychiatry*.

eFigure 5: Cost-effectiveness acceptability curve for MBCT-SH versus CBT-SH at 42-week follow-up based on sensitivity analysis with outliers removed

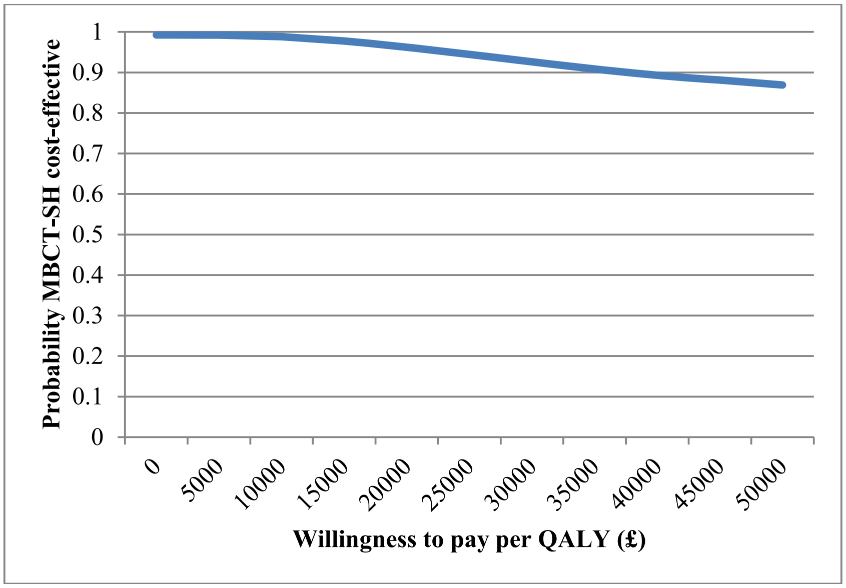

© 2023 Strauss C et al. *JAMA Psychiatry*.

eFigure 6: Cost-effectiveness acceptability curve for MBCT-SH versus CBT-SH at 42-week follow-up based on sensitivity analysis with imputed data

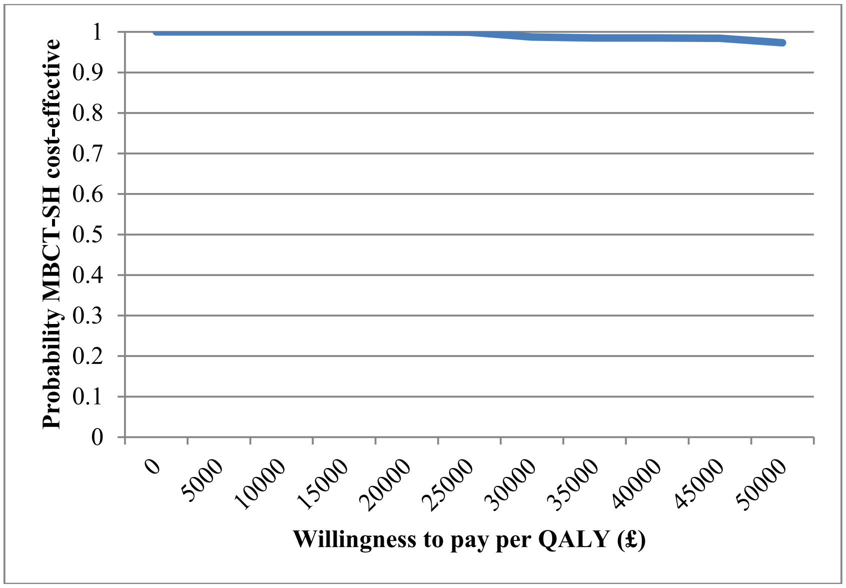

© 2023 Strauss C et al. *JAMA Psychiatry*.

eAppendix 2. The Role of Experts by Experience

<u>Recruitment of lived experience colleagues to the trial</u>

Sussex Partnership NHS Foundation Trust (UK) research department has a patient and public involvement team that supports its work. People with lived experience (EBEs) consult on the development, delivery, and dissemination of research projects. Through this a lived experience co-applicant was recruited who had experience of depression and the MBCT course. This co-applicant coordinated a group of people with lived experience who were involved in the LIGHTMind study.

LIGHTMind was preceded by a pilot study. Participants in this pilot study were asked if they would like to become members of the LIGHTMind Lived Experience Advisory Panel (LEAP), four agreed. The LEAP comprised of six people with lived experience and led by the lived experience co-applicant. Three members contributed to the design of the project prior to grant application. Additionally, two people with lived experience were recruited through to join the trial steering committe.

<u>Structure of involvement activities</u>

The lived experience co-applicant undertook the following activities for the study:

1. Prior to the trial regular meetings were held between the LEAP, members of the research team and the lived experience co-applicant in order to inform the writing of the grant application
2. Attendance by the lived experience co-applicant at the weekly operations meeting in order to bring a patient and public perspective on matters arising, and to identify where lived experience involvement was needed in decision making in the trial.
3. Attendance by the co-applicant at the team meeting.
4. Undertaking interviews with 24 participants to understand the experience of taking part in the trial, and the changes that they had experienced.
5. Involvement in the analysis of the 24 interviews.
6. Involvement in decisions about dissemination.

The LEAP members undertook the following activities:

1. A meeting with participants from the pilot study prior to the grant application for the current study to inform its development.
2. Email consultations with the lived experience co-applicant and some members of the LEAP prior to ethics submission to inform materials, particularly the Participant Information Sheet.
3. Seven meetings across the period of the trial – including three at dissemination to understand a patient and public perspective on current issues relating to the trial.
4. Regular emails between the lived experience co-applicant and the LEAP members, including giving updates on the trial and asking for opinions about recruitment activities.
5. Attendance by two lived experience consultants at the trial steering committee to offer a patient and public perspective.
6. Involvement in decisions about dissemination.

<u>Payments support and training</u>

All LEAP members:

- signed a confidentiality form and were paid according to the host NHS trust service user and carer involvement payments policy. The lived experience co-applicant held a temporary post in the trust for the period the trial was recruiting.

- were given training to help them understand the essentials of randomised control trials and the nature of the LIGHTMind study.

- were given training which helped them think about how their voice could be heard and in what ways they could contribute.

The lived experience co-applicant was given training and supervision by the Chief Investigator and the lead for Patient and Public Involvement in the research department in the host NHS trust. She supported LEAP members to be involved through regular communications and offering briefing and debriefing.

What difference did EBEs make to the LIGHTMind trial?

LEAP members were part of an iterative process of developing and supporting the trial. While the Chief Investigator had overall responsibility, no one person stands alone. The process of conducting the study has been a collaborative one where opportunities have been sought to hear from those who are patients, members of the public and have lived experience.

LEAP members were keen to understand the experiences of participants in the trial. Early on it was decided to include a qualitative study. It was decided that someone with lived experience would be best placed to ask others about how they had found being in the trial. Additionally, it was decided that having someone who had lived experience rather than research experience might understand the data differently. As a result, the lived experience co-applicant has undertaken the qualitative interviews and been involved in the analysis. This has brought a different approach to data analysis, where efforts have been made to understand what might be the components of change for participants, rather than simply looking at what has changed.

Having a user-friendly Participant Information Sheet is essential. LEAP members helped write and rewrite this sheet. This met with a degree of success. However, the information sheet still felt information heavy, and it is interesting to speculate how well participants are able to take in information, and how this could be improved in future studies.

Involvement with participants in the pilot led to an understanding of what was important for participants as they spoke to the Psychological Wellbeing Practitioner (PWP). This information was converted and used to train both PWPs and research assistants, the lived experience co-applicant helped deliver this material. A member of the lived experience advisory panel was also involved. This was valued as helping to sensitize both the PWP and research assistants to being attuned to the situation that participants found themselves in.

The Lived Experience Advisory Panel addressed a number of issues during the delivery of training. These included:

1. How to ensure the participants could make sense of the instructions for the beginning of the intervention. A short instruction leaflet was devised.
2. Development of recruitment materials to ensure simplicity and high visibility.
3. Grappling with and offering suggestions about how to manage the slow recruitment of participants. Including thinking about acceptability to patients of different approaches to recruitment in GP settings. Particularly the use of texting to recruit.
4. Discussion about research assistant approaches to offering support to potential participants in the online inclusion and exclusion computer package. This enabled a more positive experience for potential participants.
5. Support of the team in the decision to widen the trial to more host sites across the country.

What did we learn about involvement in a clinical trial?

It is our experience that effective involvement helps to ensure that participants' needs are put first. Effective involvement occurs when sufficient time is given to building relationships with patients and members of the public so that they understand the constraints imposed by the nature of a clinical trial. This includes training, actively communicating with lived experience colleagues on a regular basis and ensuring that recommendations from LEAP members are acted on, even if to explain why something cannot happen. Please see Lea et al (2020)[24] for further reflections of the role of lived experience involvement in the current study.

Conclusion

The LIGHTMind project was supported by the involvement of PPI colleagues, including a lived experience co-applicant. Involvement by PPI colleagues informed the design of the study. People with lived experience were involved in the delivery of training for the PWPs delivering the intervention and the research assistants. A lived experience co-applicant was involved in collection of data in the qualitative work and in its analysis. Effective involvement requires time, good communication and attention to power issues. This ensures that PPI colleagues can influence the project and prevents tokenism.

© 2023 Strauss C et al. *JAMA Psychiatry*.

eReferences.

1. Spitzer RL, Kroenke K, Williams JBW, Löwe B. A brief measure for assessing generalized anxiety disorder: the GAD-7. *Arch Intern Med*. 2006;166(10):1092-1097. doi:10.1001/archinte.166.10.1092
2. NHS Health Scotland & University of Warwick & University of Edinburgh. *Short Warwick-Edinburgh Mental Well-Being Scale*. Edinburgh; 2007.
3. Mundt JC. The Work and Social Adjustment Scale: a simple measure of impairment in functioning. *Br J Psychiatry*. 2002;180(5):461-464. doi:10.1192/bjp.180.5.461
4. Gu J, Strauss C, Crane C, et al. Examining the factor structure of the 39-item and 15-item versions of the five-facet mindfulness questionnaire before and after mindfulness-based cognitive therapy for people with recurrent depression. *Psychol Assess*. 2016;28(7):791-802. doi:10.1037/pas0000263
5. Boschen MJ, Drummond LM, Pillay A, Morton K. Predicting outcome of treatment for severe, treatment resistant OCD in inpatient and community settings. *J Behav Ther Exp Psychiatry*. 2010;41(2):90-95. doi:10.1016/j.jbtep.2009.10.006
6. Curtis L, Burns A. *Unit Costs of Health and Social Care*. Canterbury, UK; 2018.
7. NHS Improvement. *NHS Improvement 2017/18 Reference Costs*.; 2020. https://xn--improvementnhsuk-b1ad/resources/reference-costs/ [accessed: 27/03/2020].
8. NHS Digital. *Prescription Cost Analysis - England, 2018*.; 2019. https://xn--digitalnhsuk-wuad/data-and-information/publications/statistical/prescription-cost-analysis/2018 [accessed 27/03/2020].
9. Radhakrishnan M, Hammond G, Jones PB, Watson A, McMillan-Shields F, Lafortune L. Cost of improving Access to Psychological Therapies (IAPT) programme: an analysis of cost of session, treatment and recovery in selected Primary Care Trusts in the East of England region. *Behav Res Ther*. 2013;51(1):37-45. doi:10.1016/j.brat.2012.10.001
10. Turner HC, Lauer JA, Tran BX, Teerawattananon Y, Jit M. Adjusting for Inflation and Currency Changes Within Health Economic Studies. *Value Heal*. 2019;22(9):1026-1032. doi:10.1016/J.JVAL.2019.03.021
11. Nolan G. *Developing a Local Tariff for IAPT Services in NHS East of England*. Cambridge, UK; 2009.
12. Health and Social Care Information Centre (HSCIC). *Psychological Therapies, Annual Report on the Use of IAPT Services: England– 2013/14*.; 2014.
13. Ekers D, Godfrey C, Gilbody S, et al. Cost utility of behavioural activation delivered by the non-specialist. *Br J Psychiatry*. 2011;199(6):510-511. doi:10.1192/BJP.BP.110.090266
14. Curtis L, Burns A. *Unit Costs of Health and Social Care*. Canterbury, UK; 2015.
15. Curtis L, Burns A. *Unit Costs of Health and Social Care*. Canterbury, UK; 2012.
16. Thompson SG, Barber JA. How should cost data in pragmatic randomised trials be analysed? *BMJ*. 2000;320(7243):1197-1200. doi:10.1136/bmj.320.7243.1197
17. Manca A, Hawkins N, Sculpher MJ. Estimating mean QALYs in trial-based cost-effectiveness analysis: the importance of controlling for baseline utility. *Health Econ*. 2005;14(5):487-496. doi:10.1002/hec.944
18. NICE. *Position Statement on Use of the EQ-5D-5L Value Set for England (Updated October 2019)*.; 2019.
19. Van Hout B, Janssen MF, Feng YS, et al. Interim Scoring for the EQ-5D-5L: Mapping the EQ-5D-5L to EQ-5D-3L Value Sets. *Value Heal*. 2012;15(5):708-715. doi:10.1016/J.JVAL.2012.02.008
20. Briggs AH. A Bayesian approach to stochastic cost-effectiveness analysis. *Health Econ*. 1999;8(3):257-261. doi:10.1002/(SICI)1099-1050(199905)8:3<257::AID-HEC427>3.0.CO;2-E
21. Fenwick E, Byford S. A guide to cost-effectiveness acceptability curves. *Br J Psychiatry*. 2005;187(AUG.):106-108. doi:10.1192/bjp.187.2.106
22. Assmann SF, Pocock SJ, Enos LE, Kasten LE. Subgroup analysis and other (mis)uses of baseline data in clinical trials. *Lancet*. 2000;355(9209):1064-1069. doi:10.1016/S0140-6736(00)02039-0
23. Weichle T, Hynes DM, Durazo-Arvizu R, Tarlov E, Zhang Q. Impact of alternative approaches to assess outlying and influential observations on health care costs. *Springerplus*. 2013;2(1):1-11. doi:10.1186/2193-1801-2-614/FIGURES/3
24. Leeuwerik T, Cavanagh K, Forrester E, et al. Participant perspectives on the acceptability and effectiveness of mindfulness-based cognitive behaviour therapy approaches for obsessive compulsive disorder. *PLoS One*. 2020;15(10). doi:10.1371/journal.pone.0238845

© 2023 Strauss C et al. *JAMA Psychiatry*.
