# Supplementary material for: Clinical Effectiveness and Cost-Effectiveness of Supported Mindfulness-Based Cognitive Therapy Self-help Compared With Supported Cognitive Behavioral Therapy Self-help for Adults Experiencing Depression: The Low-Intensity Guided Help Through Mindfulness (LIGHTMind) Randomized Clinical Trial
Source: JAMA Psychiatry. 2023 Mar 22;80(5):415–24. doi: 10.1001/jamapsychiatry.2023.0222 (PMC10034662; doi:10.1001/jamapsychiatry.2023.0222)
Supplement: Supplement 4. — Data Sharing Statement [file jamapsychiatry-e230222-s004.pdf]

Data Sharing Statement

Strauss. Clinical Effectiveness and Cost-Effectiveness of Supported Mindfulness-Based Cognitive Therapy Self-help Compared With Supported Cognitive Behavioral Therapy Self-help for Adults Experiencing Depression. *JAMA Psychiatry*. Published March 22, 2023. doi:10.1001/jamapsychiatry.2023.0222

Data
Data available: Yes
Data types: Deidentified participant data
How to access data: Data will be made available on the University of Sussex repository (https://sussex.figshare.com/)
When available: beginning date: 22-03-2024

Supporting Documents
Document types: Informed consent form
How to access documents: A blank copy of the informed consent form will be made available on the University of Sussex repository (https://sussex.figshare.com/)
When available: With publication

Additional Information
Who can access the data: On acceptance of this manuscript, a password-protected disclosure-controlled version of the trial data will be uploaded to the University of Sussex repository. Access to this will be embargoed for 12 months from the date of publication to enable the research team to write planned papers.
Types of analyses: Data will be made available for any purpose.
Mechanisms of data availability: Researchers from other research teams will be expected to submit a project proposal to the study Chief Investigator (first author) to request access to trial data.
